# Response of Collembola and Acari communities to summer flooding in a grassland plant diversity experiment

**DOI:** 10.1371/journal.pone.0202862

**Published:** 2018-08-30

**Authors:** Odette González-Macé, Stefan Scheu

**Affiliations:** 1 University of Göttingen, J.F. Blumenbach Institute of Zoology and Anthropology, Göttingen, Germany; 2 University of Göttingen, Centre of Biodiversity and Sustainable Land Use, Göttingen, Germany; Nanjing Agricultural University, CHINA

## Abstract

Flooding frequency is predicted to increase during the next decades in Europe. Therefore, it is important to understand how short-term disturbance events affect soil biota providing essential ecosystem functions and uncover factors modulating their response such as plant community composition. Here we report on the response of soil microarthropod communities (Collembola and Acari) to a severe summer flood in 2013, which affected major parts of central Europe. Collembola and Acari density and Collembola and Oribatida richness were strongly affected by the flood, but they recovered within three months. Effects of plant community composition on soil microarthropods disappeared after the flood, presumably due to homogenization of the field, but the effects of plant community were in a stage of being reasserted three months after the flood. Widespread, surface living and generalistic microarthropod species recolonized the field quickly. Prostigmata and Oribatida were more resilient or recovered to flooding than Astigmata and Gamasida. Long-term impacts, however, remain unknown and deserve further investigation.

## Introduction

The increasing likelihood of extreme climate events with ongoing climate change is expected to have major impacts on biodiversity at local scales [1]. Extreme climate events will primarily consist of periods of heat, cold, drought and flooding with greater severity and less predictability than historical norms [2]. These events will act as disturbance and are likely to decrease biodiversity at local as well as regional scales.

Floods are projected to increase with global warming in the 21st century leading to rapid changes in soil conditions thereby detrimentally affecting soil microorganisms [3] by limiting soil gas diffusion and oxygen availability thus reducing soil nutrient availability, mineralization and decomposition of dead organic material [4]. As a consequence, anaerobic conditions develop quickly in flooded soils [5] resulting in marked changes in soil chemistry [6] including the accumulation of toxic substances [4]. All these changes are likely to significantly affect the composition of soil food webs. To explore the effects of flooding on soil food webs and, more specifically, soil microarthropods, we investigated a severe natural flooding event in a grassland plant diversity experiment [7]. The flooding, resulting from heavy summer precipitation, was accompanied by the input of sediments rich in nutrients and was associated by an unexpectedly fast recovery of soil microorganisms within three months [8]. Fungal biomass increased, reflecting elevated availability of dead plant biomass [9]. Further, flooding was associated with enhanced plant community productivity but decreased stability, particularly of plant communities of high diversity [10].

Until today understanding how short-term disturbance events affect soil biodiversity is limited [11,12] but is important as changes in soil biodiversity and community structure impact the functioning of soils [13,14]. A major component of soil animal communities are microarthropods reaching high density and diversity in any kind of soil, and playing a crucial role in driving belowground ecosystem processes such as decomposition and nutrient cycling [13,15]. Microarthropods, such as Acari and Collembola, are major animal groups interacting with soil microorganisms [16,17]. Microarthropod species are likely to be differentially affected by changes in environmental conditions such as inundation events depending on physiological adaptations and life history traits [18,19]. Although Collembola (Insecta) and Oribatida (Acari) are often grouped into the same trophic level and are considered to occupy similar niches in decomposition processes [20,21], the two groups differ in a variety of ecological traits including mobility, reproduction, level of predation pressure, and tolerance to abiotic conditions [18,22,23]. Parthenogenesis may facilitate quick population establishment after disturbances and is most widespread in Oribatida [24,25]. However, the general life-history traits of Oribatida have been considered typical of K-selected species [24], whereas Collembola species exhibit wider variation in life-history traits [26]. In particular, compared to Collembola, Oribatida species are less mobile, characterized by low reproductive rates and recolonize disturbed habitats slowly [27]. Collembola, in contrast, are more sensitive than Oribatida to abiotic microhabitat conditions and recolonize disturbed habitats more quickly[18,28]. Other Acari such as Astigmata have short developmental time and excellent dispersal ability [29]. They feed on fungi or bacteria, but may also consume plant tissue [29]. Gamasida are mostly free-living predators [30] but also parasites or symbionts [31]. Prostigmata are predators, herbivores and parasites [29].

In this study, we focus on the response of Acari and Collembola to flooding in grasslands of varying plant diversity. We expected the density and richness of Collembola and Acari communities to be reduced strongly by flooding with Collembola recovering faster than Acari due to higher reproductive potential and dispersal ability. We further expected that surface-living Collembola species with high dispersal ability will recover faster than species living deeper in soil [32]. Among Acari we expected Astigmata, Prostigmata and Gamasida to recover faster than Oribatida due to generally faster reproductive cycles. We further expected the immediate effects of flooding to be similar in both Collembola and Acari and to be independent of plant species diversity. However, we expected the recovery to be facilitated by high plant species diversity in particular in Collembola. Collembola density and diversity have been shown to benefit from plant diversity due to increased root and microbial biomass, and elevated quantity and quality of plant residues serving as food resources[33,34].

## Material and methods

### Experimental setup

The Jena Experiment is a semi-natural temperate grassland on the floodplain of the Saale River close to the city of Jena (50°55´ N, 11°35´ E; Thuringia, Germany). Mean annual air temperature is 9.9°C and mean annual precipitation is 610 mm (1980–2010). The study site, a Eutric Fluvisol, has been used as an arable field for over 40 years before the experiment was established with typical Central European hay meadow plants in 2002. The experiment comprises 80 5 x 6 m plots arranged in 4 blocks to control for changes in soil texture with distance from the river. A gradient of plant species richness (1, 2, 4, 8, 16 and 60) and plant functional group richness (1, 2, 3 and 4) was established (Table 1). Plant species are grouped according to the morphological, phenological and physiological traits into grasses (16 species), small herbs (12 species), tall herbs (20 species) and legumes (12 species). The established grassland is mown twice a year and weeded three times per year [35]. No permission was needed to take the samples from this site. The site is rented and managed by the project and for taking samples for analysing soil arthropods from arable systems no permission from legal bodies is needed. The field studies did not involve endangered or protected species.

**Table 1:** Design of the Jena experiment.

		Plant species richness						
		1	2	4	8	16	60	Total
Plant functional group richness	**1**	14	8	4	4	2	-	32
	**2**	-	8	4	4	4	-	20
	**3**	-	-	4	4	4	-	12
	**4**	-	-	4	4	4	4	16
	Total	14	16	16	16	14	4	80

Combinations of plant species richness and plant functional group richness and number of replicates per richness level. For more details on the experimental design see Roscher et al. (2004).

### Flooding

The June 2013 flood in the Upper Danube Basin was one of the largest floods in the past two centuries [7]. Rainfall in May 2013 in southeast Germany was exceptionally high. In Jena it amounted to approximatively 150 mm. High rainfall resulted in the flooding of the Saale River with the flood also covering the Jena Experiment field site and lasting for 25 days (30 May to 24 June). Flooding caused anaerobic soil conditions with redox potentials ranging from -121 to 193 mV in some plots [10]. Water coverage was measured daily for each plot from 31 May to 24 June and ascribed to 5 levels: 0, 25, 50, 75 and 100% (percentage of the plot covered by water). Flooding severity was measured using a flooding index calculated as the sum of daily percentages for the whole flooding period (24 days) [9]. After the flood in August 2013, dead material, target species, weeds and bare ground percentage of the plot was measured. In general, we found that 78% of the plots was covered by target species, 14% of weeds, 8% of dead material and 23% of bare ground. Monocultures had only 41% of target species and 50% of bare ground. In contrast, plots with 16 plant species had 88% of the plot covered by target species and 7% covered by bare ground. In October the vegetation was recovered totally.

### Soil biota

In November 2010, July 2013 (three weeks after the flood) and in October 2013 (three months after the flood), soil cores of 5 cm diameter and 5 cm depth were taken from each plot using a stainless steel corer (80 samples per date). Soil microarthropod species were extracted using a high-gradient extractor [36], increasing the temperature gradually from 25 to 55°C during 14 days. The animals were collected in mono-ethyleneglycol and transferred into 70% ethanol for preservation. Acari were sorted into Oribatida, Gamasida, Prostigmata and Astigmata. Oribatida were identified to species level using Weigmann [37] and Collembola were identified to species level using Hopkin [26] and Fjellberg [38,39]. For identification a light microscope (Axioplan; Zeiss, Germany) with up to ×1000 magnification was used. For full list of species including authorities see supplementary material (S1 and S2 Tables). Species richness (number of species; S3 and S4 Tables) and density (number of individuals per square meter; S5 and S6 Tables) for Collembola and Oribatida were calculated.

### Data analysis

To improve homogeneity of variances, data on abundance (individuals per soil core) and species richness were log_10_ (x+1) transformed prior to statistical analysis. The 60 plant species mixtures were excluded from the statistical analysis due to insufficient number of replicates (four replicates at the field site, each being differentially affected by the flood; [10]). Linear models (type I sum of squares) were used to analyze effects of block (categorical variable, 4 blocks), flooding index (continuous variable, from 1 to 23 days), dead organic material, target species, weeds and bare ground (continuous variable, percentage of the plot, only used in October 2013), plant functional group richness (continuous variable, from 1 to 4), plant species richness (continuous variable, from 1 to 16, log-transformed) and presence/absence of grasses, legumes, small herbs and tall herbs (categorical variables) on the density and richness of Collembola and Acari, the density of Astigmata, Gamasida, Oribatida and Prostigmata (suborders of Acari) and the density of most abundant families of Collembola (Entomobrydae, Isotomidae and Tullbergiidae) for the data of 2010 and 2013 (three months after the flood). Due to very low density three weeks after the flood these data were not analyzed statistically. The full model with the lowest Akaike Information Criterion (AIC) was selected as the best starting model [40,41]. This model was simplified in a stepwise manner by dropping non-significant variables. Although the experimental design was set up as orthogonal as possible, there is collinearity between functional group richness of plants and the presence/absence of individual functional groups [42], which we quantified using the inflation factor (VIF) from the car package [43]. The analysis suggested to exclude functional group richness if there are two or more functional groups in the model (VIF ~ 4). Therefore, functional group richness was added after model simplification and was only included in the final model if it improved the model significantly (principle of Occam’s Razor, p < 0.05). Generally, block and flooding index were fitted first followed by plant species richness; thereafter presence/absence of grasses, legumes, tall herbs and short herbs were fitted. F-values given in text and tables generally refer to those where the respective factor was fitted first [44]. Statistical analyses were performed using R 3.2.1 [45].

Data on Collembola species were analyzed using non-metric multidimensional scaling (NMDS with Bray-Curtis distance) reducing the number of dimensions to four. To identify the factors which drive Collembola community composition, the four dimensions were further analyzed by MANOVA. In addition, discriminant function analysis (DFA) was carried out on four NMDS axes with Statistica 13 (Statsoft, Inc., Tulsa, Oklahoma, USA). Plant species and plant functional group richness were used as variables of discrimination. Squared Mahalanobis distances between groups were calculated to identify differences between plant richness levels.

The community structure of Collembola and Oribatida was analyzed using principal component analysis (PCA) as implemented in CANOCO 5 (Microcomputer Power, Ithaca, NY; [46]) using the abundance of species which appeared more than in three samples. Moreover, we correlate the factors and the axes of each PCA using Pearson correlation.

## Results

### Collembola

**Fig 1:**
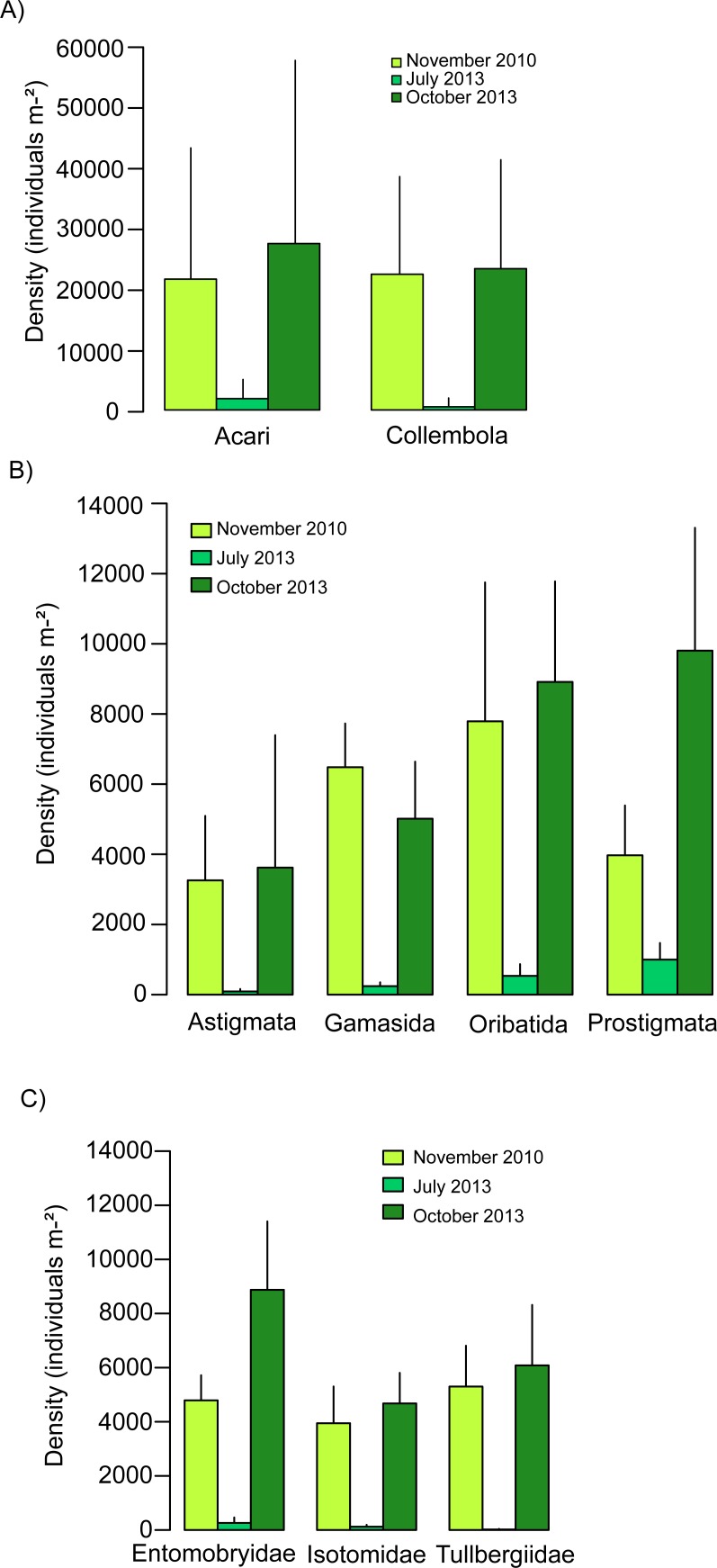
Density of Acari and Collembola. Density of (A) Acari and Collembola, (B) Acari suborders (Astigmata, Gamasida, Oribatida and Prostigmata) and (C) Collembola families (Entomobryidae, Isotomidae, Tullbergiidae) in November 2010 (before the flood, 80 samples), July 2013 (three weeks after the flood, 80 samples) and October 2013 (three months after the flood, 80 samples). Values are means ± SE.

In November 2010 Collembola density was 22,310 ± 15,975 individuals m-^2^, whereas three weeks after the flood in 2013 it was only 515±1347 individuals m-^2^(mean ± SE). In contrast, three months after the flood Collembola density (23,220 ± 17,826 individuals m-^2^; mean ± SE) was similar to the level in 2010 (Fig 1A and 1C). Collembola density in 2010 was not influenced by experimental treatments, but three months after the flood in 2013 it increased slightly with plant species richness (F_1,76_ = 2.97; Table 2). Moreover, dead organic material, target species, weeds and bare ground percentage of the plot was not significant in the density of October 2013.

**Table 2:** F-values of linear models of Acari and Collembola.

	Acari								Collembola							
	Acari density				Oribatida richness				Collembola density				Collembola richness			
Year	2010		2013		2010		2013		2010		2013		2010		2013	
	df	F	df	F	df	F	df	F	df	F	df	F	df	F	df	F
Block	3,76	**4.83**		-	1,76	**9.76**		-		-		-		-		-
Flooding index		-		-		-	1,76	*↓2*,*86*		-		-		-		-
Plant species richness		-		-	1,76	**↑6.30**		-		-	1,76	↑*2*.*97*	1,76	**↑***3*.*76*		-
Plant functional group richness		-		-	1,76	**↑5.18**		-		-		-	1,76	**↑***2*.*82*		-
Grasses	1,76	**↑6.19**	1,76	**↑5.27**	** ** **1,76**	**↑***2*.*94*	1,76	*↑3*,*04*		-		-		-		-
Small herbs		-	1,76	**↑4.06**	** ** ** **	-		-		-		-		-		-
Tall herbs		-		-		-		-		-		-		-	1,76	**↑4.54**

Table of F-values of linear models on the effect of block, flooding index, plant species richness, plant functional group richness, presence of grasses, small herbs and tall herbs on the density and richness of Acari and Collembola in November 2010 (before the flood) and October 2013 (three months after the flood). Significant effects are given in bold (P < 0.05) and marginally significant effects in italics (P<0.10). F-values represent those where the respective factor was fitted first (see Methods).

**Fig 2:**
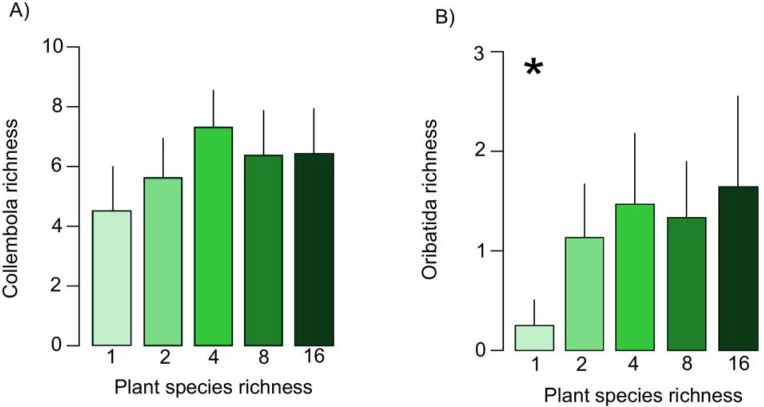
Effect of plant species richness on species richness of Collembola and Oribatida. Variations in species richness of (A) Collembola and (B) Oribatida with plant species richness (1, 2, 4, 8, 16 species with 14, 16, 16, 16 and 14 replicates, respectively) in November 2010. Values are means ± SE. For statistical analysis see Table 2.

In 2010 a total of 27 species of Collembola were recorded, while only 16 species were recorded three weeks after the flood. However, three months after the flood species number increased to 22 (S1 Table). In 2010 Collembola species richness increased marginally significant with plant species richness (F_1,76_ = 3.76; Fig 2A) and plant functional group richness (F_1,76_ = 2.82; Table 2). There was no significant effect of plant species and plant functional group richness on Collembola species richness three months after the flood in 2013, but increased significantly with the presence of tall herbs (Table 2). Dead organic material, target species, weeds and bare ground percentage of the plot was not significant in the species richness of October 2013.

**Fig 3:**
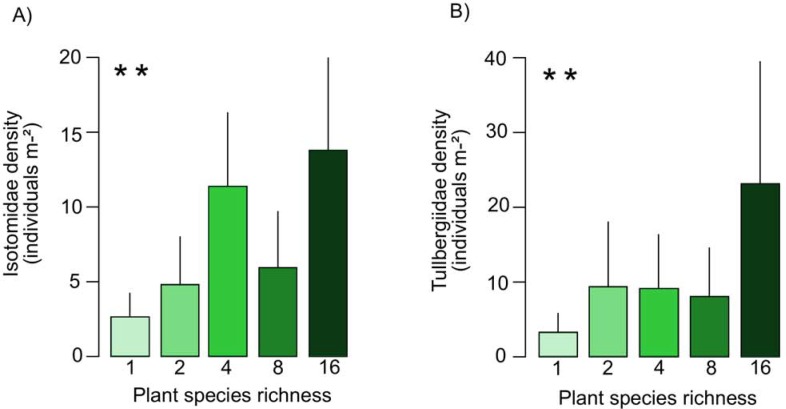
Effects of plant species richness on the density of Isotomidae and Tullbergiidae. Variations in density of (A) Isotomidae in November 2010 and (B) Tullbergiidae in October 2013 with plant species richness (1, 2, 4, 8, 16 with 14, 16, 16, 16 and 14 replicates, respectively). Values are means ± SE. For statistical analysis see Table 3.

In 2010 the density of the most abundant family of Collembola, Isotomidae, increased significantly with plant species richness (Fig 3A) and also in presence of grasses, but decreased in the presence of tall herbs. In contrast, the densities of Entomobryidae and Tullbergiidae were not significantly affected by experimental treatments in 2010 (Table 3). Three months after the flood in 2013 the density of Entomobryidae increased with flooding index (F_1,73_ = 3.88). Further, the density of Tullbergiidae increased significantly with plant species richness (Fig 3B) and decreased slightly with the presence of grasses (F_1,73_ = 3.28; Table 3). In contrast to 2010, Isotomidae were not significantly affected by experimental treatments in 2013.

**Table 3:** F-values of linear models on the density of Entomobryidae, Isotomidae and Tullbergiidae.

	Entomobryidae				Isotomidae				Tullbergiidae			
Year	2010		2013		2010		2013		2010		2013	
	df	F	df	F	df	F	df	F	df	F	df	F
Block		-		-		-		-		-	3,73	**3.11**
Flooding index		*-*	1,73	**↑***3*.*88*		**-**		-		-		-
Plant species richness		-		-	1,73	**↑7.18**		-		-	1,73	**↑9.57**
Grasses		-		-	1,73	**↑4.53**		-		-	1,73	*↓3*.*28*
Tall herbs		-		-	1,73	*↓***4.65**		-		-		-

Table of F-values of linear models on the effect of block, flooding index, plant species richness, presence of grasses and tall herbs on the density of Entomobryidae, Isotomidae and Tullbergiidae in November 2010 (before the flood) and October 2013 (three months after the flood). Significant effects (P < 0.05) are given in bold and marginally significant effects (P<0.10) in italics. F-values represent those where the respective factor was fitted first (see Methods).

In 2010, Collembola community composition changed significantly with plant species richness (F_1,76_ = 5.33, P < 0.01) and plant functional group richness (F_1,76_ = 4.35, P < 0.01). Collembola community (number of species and species composition) was similar at higher plant species richness but less variable in the one and two species treatments (Table 4A). Similarly, community composition of Collembola differed between plant functional group one and four as well as two and four (Table 4B).

**Table 4:** Squared Mahalanobis distances.

A					
Plant species richness	1	2	4	8	16
1	-	1.95*	1.72	2.45**	1.47
2		-	2.56**	1.59*	2.43**
4			-	0.68	0.88
8				-	0.92
16					-
B					
Plant functional group	1	2	3	4	
1	-	0.82	1.02	1.68**	
2		-	0.97	1.41*	
3			-	0.50	
4				-	

Squared Mahalanobis distances between group centroids and reliability of discrimination for Collembola species composition in November 2010 of (A) plant species richness (SR), (B) plant functional group richness (FG)

**P < 0.05

*P < 0.10.

**Fig 4:**
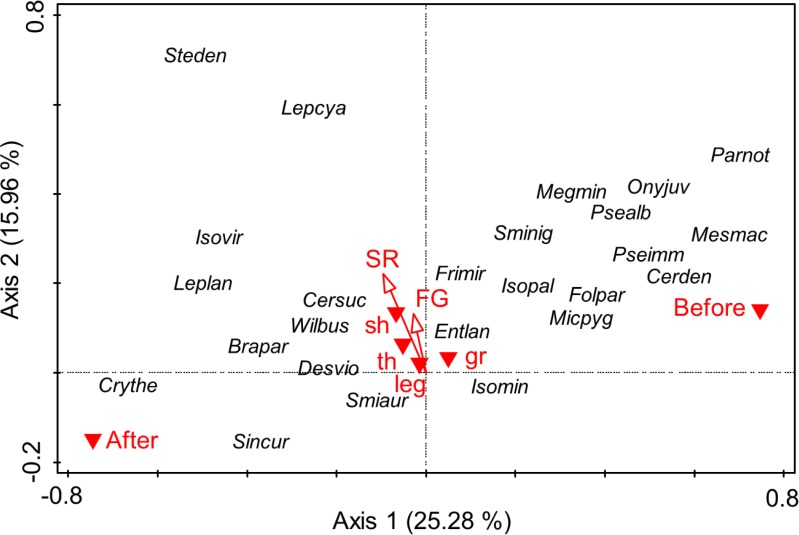
PCA of Collembola species. Principal components analysis (PCA) of Collembola species in plant communities in November 2010 (before the flood) and October 2013 (three months after flood). Arrows indicate variations with plant species (SR) and plant functional group richness (FG). Variations with the presence of grasses (gr), legumes (leg), small (sh) and tall herbs (th) and between November 2010 (before) and October 2013 (after) are indicated by red triangles. For full species names see S1 Table.

PCA separated Collembola communities mainly along the first axis representing 25.28% of the variability in species data, whereas the second axis represented 15.96% of the variability (Fig 4). Separation along the first axis mainly represents differences between Collembola communities in 2010 and three months after the flood in 2013 (r = - 0.75). The most abundant species before the flood compared to 2013 were *Parisotoma notabilis*, *Mesaphorura macrochaeta*, *Ceratophysella denticulata* and *Onychiurus jubilarius*. After the flood the most abundant species compared to 2010 were *Lepidocyrtus lanuginosus* and *Cryptopygus thermophilus*. The second axis represents differences between plant species richness (r = 0.22), plant functional group richness (r = 0.13) and presence/absence of small herbs (r = 0.16). In general, at higher plant species richness *Lepidocyrtus cyaneus* and *Stenaphorura denisi* were more abundant. Moreover, there were some species present at each of the sampling dates including *Lepidocyrtus lanuginosus*, *Lepidocyrtus cyaneus* and *Willowsia buski* (Entomobryidae) as well as *Isotoma viridis*, *Parisotoma notabilis* and *Isotomiella minor* (Isotomidae). Other species like *Hypogastrura manubrialis*, *Ceratophysella engadinensis* (Hypogastruridae), *Isotomurus fucicolus*, *Proisotoma minuta* (Isotomidae), *Paratullbergia macdougalli* (Tullbergiidae), *Protaphorura armata* (Onychiuridae) and *Sminthurus viridis* (Sminthuridae) were present only three weeks after the flood.

### Acari

In November 2010 Acari density was 21,500 ± 23,290 individuals m-^2^, but only 1,864 ± 3,059 individuals m-^2^ three weeks after the flood in 2013(mean ± SE). In contrast, similar to Collembola, three months after the flood in 2013 (27,350 ± 30,040 individuals m-^2^; mean ± SE) it was similar to the level in 2010 (Fig 1A). In 2010 and three months after the flood in 2013, Acari density increased significantly with the presence of grasses. Moreover, three months after the flood in 2013 it was significantly higher with the presence of small herbs (Table 2). The Acari density of October 2013 was not affected significantly by dead organic material, target species, weeds and bare ground percentage of the plot.

The density of each of the suborders of Acari (Oribatida, Gamasida, Astigmata, Prostigmata) decreased significantly three weeks after the flood in 2013. In contrast, three months after the flood in 2013 the density of each of the Acari suborders reached a similar level than in 2010, except of Prostigmata which exceeded the density in 2010 by more than a factor of two (Fig 1B).

A total of 12 species of Oribatida were recorded in 2010, but only 9 species were recorded three weeks after the flood in 2013. However, similar to Collembola, Oribatida species also recovered quickly with 12 species being present three months after the flood in 2013 (S2 Table). In 2010 Oribatida richness increased significantly with plant species richness (Fig 2B) and plant functional group richness. Further, in 2010 (F _1,76_ = 2.94) as well as three months after the flood in 2013 (F _1,76_ = 3.04) Oribatida richness increased with the presence of grasses, however, only slightly (Table 2). Three months after the flood in 2013 Oribatida richness was slightly reduced at higher flooding index (F _1,76_ = 2.86; Table 2). Furthermore, dead organic material, target species, weeds and bare ground percentage of the plot was not significant in Oribatida species richness of October 2013.

**Fig 5:**
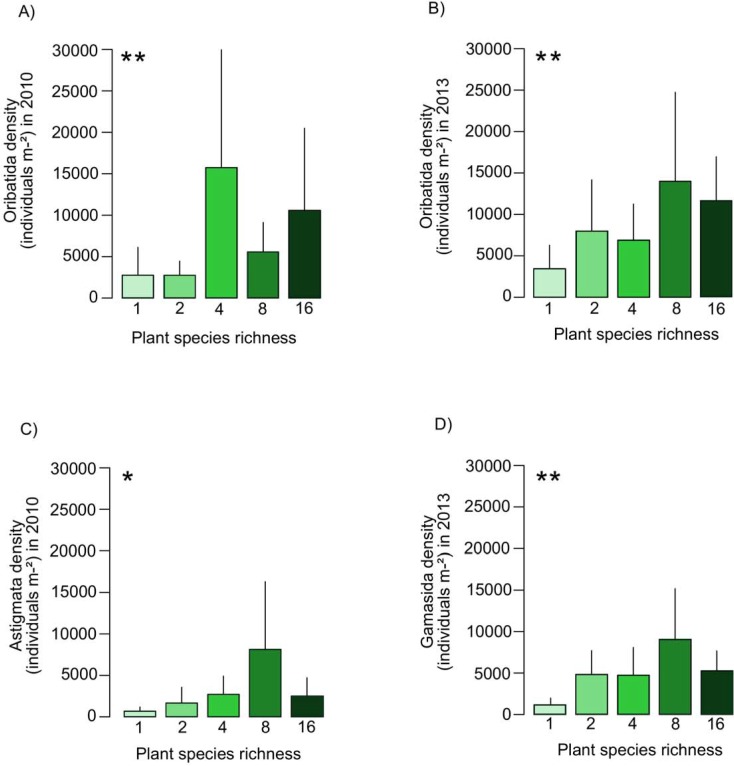
Effects of plant species richness on the density of Oribatida, Astigmata and Gamasida. Variation in density of (A) Oribatida in November 2010, (B) Oribatida in October 2013 (three months after the flood), (C) Astigmata in November 2010, and (D) Gamasida in October 2013 with plant species richness (1, 2, 4, 8, 16 with 14, 16, 16, 16 and 14 replicates, respectively). Values are means ± SE. For statistical analysis see Table 5.

Oribatida density increased significantly with plant species richness both in 2010 and three months after the flood in 2013 (Fig 5A and 5B). Moreover, in 2010 Oribatida density increased significantly with plant functional group richness and the presence of grasses (Table 5). In 2010 Gamasida density decreased slightly with the presence of tall herbs (F _1,70_ = 3.58), while in 2013 it increased significantly with plant species richness (Fig 5D) and plant functional group richness as well as the presence of legumes and slightly small herbs (F _1,70_ = 3.01; Table 5). Prostigmata density increased slightly with plant functional group richness (F _1,70_ = 3.75) and significantly in presence of grasses but only in 2010. In 2013, it was not significantly affected by experimental treatments (Table 5). Astigmata density was significantly higher at higher plant species richness (Fig 5C) and in presence of grasses in 2010 (F _1,70_ = 3.59) but not in 2013 (Table 5).

**Table 5:** F-values of linear models.

	Astigmata				Gamasida				Oribatida				Prostigmata			
Year	2010		2013		2010		2013		2010		2013		2010		2013	
	df	F	df	F	df	F	df	F	df	F	df	F	df	F	df	F
Block	-	-	-	-	3,70	**3.64**	-	-	3,70	**6.42**	-	-	3,70	**6.97**	-	-
Plant species richness	1,70	**↑6.24**	-	-	-	-	1,76	**↑13.18**	1,70	**↑8.77**	1,76	**↑10.46**	-	-	-	-
Plant functional group richness	-	-	-	-	-	-	1,76	**↑16.74**	1,70	**↑6.28**	-	-	1,70	**↑***3*.*75*	-	-
Grasses	1,70	***↑****3*.*59*	-	-	-	-	-	-	1,70	**↑4.49**	-	-	1,70	**↑5.17**	-	-
Legumes	-	-	-	-	-	-	1,76	**↑3.99**	-	-	-	-	-	-	-	-
Small herbs	-	-	-	-	-	-	1,76	**↑***3*.*01*	-	-	-	-	-	-	-	-
Tall herbs	-	-	-	-	1,70	*↓3*.*58*	-	-	-	-	-	-	-	-	-	-

Table of F-values of linear models on the effect of block, flooding index, plant species richness, plant functional group richness, presence of grasses, legumes, small herbs and tall herbs on the density of Astigmata, Gamasida, Oribatida and Prostigmata in November 2010 (before the flood) and October 2013 (three months after the flood). Significant effects are given in bold (P < 0.05) and marginally significant effects in italics (P<0.10). F-values represent those where the respective factor was fitted first (see Methods).

**Fig 6:**
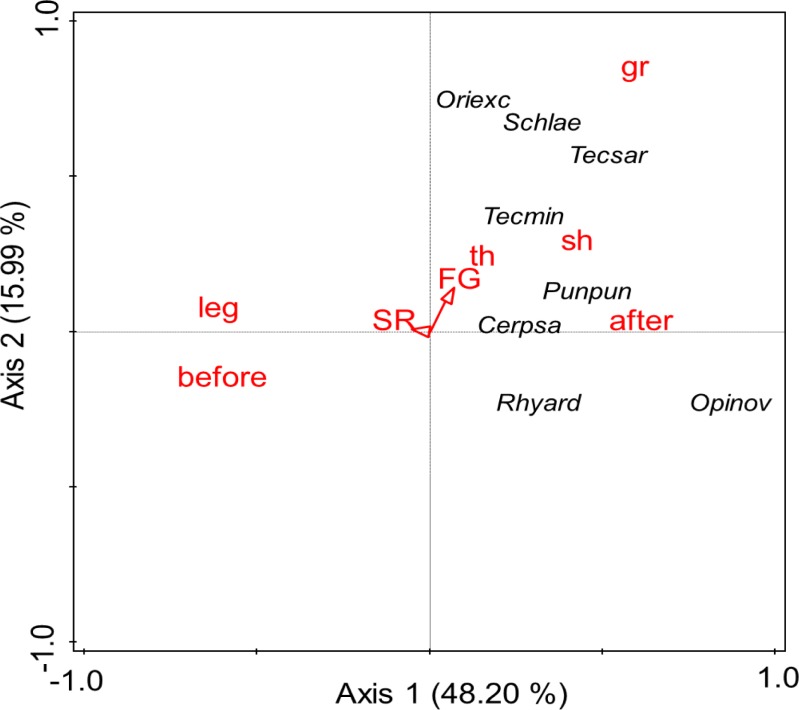
PCA of Oribatida species. Principal components analysis (PCA) of Oribatida species in plant communities in November 2010 (before the flood) and October 2013 (three months after flood). Arrows indicate variations with plant species (SR) and plant functional group richness (FG). Variations with the presence of grasses (gr), legumes (leg), small (sh) and tall herbs (th) and between November 2010 (before) and October 2013 (after) are indicated by red triangles. For full species names see S2 Table.

PCA separated Oribatida species along the first axis explaining 48.2% of the variability in species data and the second axis representing 15.99% of the variability in species data (Fig 6). Separation along the first axis mostly represents differences between Oribatida communities in 2010 and three months after the flood in 2013 (r = 0.11) and the presence of legumes (r = 0.13) and grasses (r = 0.15). The second axis mainly represents differences with plant functional group richness (r = 0.14) and presence of grasses (r = 0.19). Separation along the first axis were due to e.g., *Oppiella nova* being more abundant three months after the flood in 2013. Separation along the second axis was due to e.g., higher numbers of *Oribatula excavata* in plant communities with grasses. Moreover, we found some species present at each of the sampling including *Oppiella nova*, *Tectocepheus sarekensis*, *Oribatula excavata*, *Rhysotritia ardua* and *Punctoribates punctum* as well as species present only three weeks after the flood like *Zygoribatula frisiae* and *Schleloribates initialis*.

## Discussion

Collembola and Acari density as well as Collembola and Oribatida species richness were affected drastically by the flood but recovered quickly, returning within three months to levels recorded three years earlier (2010), despite of the seasonal changes of the community, since generally the Collembola and Acari abundance in autumn is higher than in summer ([33]; S1 Fig). This reinforces assumptions that microarthropods respond rapidly to environmental changes [47] and recover quickly [48]. In 2010 Collembola and Oribatida species richness increased with plant species and plant functional group richness, but these interrelationships were absent three months after the flood. This suggests that flooding resulted in homogenization of environmental conditions, eradicating effects of plant community composition established before the flood. However, three months after the flood Collembola species richness was increased in the presence of tall herbs, and Oribatida density and richness were consistently affected by grasses, indicating that effects of plant community composition were in a stage of being reasserted.

As hypothesized, Entomobryidae, as epedaphic species, may benefit from their dispersal ability thereby quickly recovering after the flood, presumably taking advantage of increased fungal biomass in the most severely flooded plots [9]. In contrast to Entomobryidae, the euedaphic and hemiedaphic Tullbergiidae and Isotomidae require habitable pore space [49] and this likely contributed to the delayed recolonization of the clogged flooded soils. They also are more reliant on the recovery of the plant community. Isotomidae and Tullbergiidae are sensitive to soil quality and root exudates [50], and are assumed to benefit from increased root biomass and associated exudates in more diverse plant communities [51]. In 2010 the density of Isotomidae increased in presence of grasses and tall herbs. Grasses increase root and microbial biomass, both likely contributing to increased food resource supply to Collembola [33,52,53].

Oribatid mites are decomposers and have been used as indicators of soil stability and fertility [54]. Generally, they have low metabolic rates, slow development and low fecundity. Consequently, they are vulnerable to disturbances[55] and recover slowly thereafter[56,57]. However, higher densities of Prostigmata and Oribatida than of Astigmata and Gamasida three weeks after the flood suggest that the former two taxa are more resistant or recovered faster to flooding than the latter. Detrimental effects of flooding may have been alleviated by dead plant biomass functioning as shelter for them [10]. Gamasida are important predators of nematodes, which were negatively affected by the flood [9]. Moreover, ant activity was not influenced by the flood and Gamasida are negatively affected by high ant activity [58,59]. Notably, three months after the flood, the density of Prostigmata was twice that in 2010. Prostigmata are dominant Acari predators with a crucial role in soil food webs important as biological control agents [31]. Some species are known to benefit from habitat disturbance [60]. Prostigmata, Gamasida and Astigmata are assumed to be r-strategists frequently occurring in disturbed habitats [61] and quickly colonizing new habitats due to high dispersal ability, high fecundity and fast development [24]. However, in contrast to Prostigmata, Gamasida and Astigmata did not take advantage of the flood. The majority of Gamasida are mobile predators feeding on Nematoda, Collembola, Enchytraeidae, larvae of Insecta and Acari [62]. Conform to their assumed sensitivity to environmental changes [63], their density increased with plant species and plant functional group richness as well as in presence of legumes three months after the flood in 2013. This likely resulted from increased availability of prey such as Collembola (Isotomidae) and nematodes both increasing significantly with plant diversity three months after the flood [9].

As ecosystems develop after disturbances, changes in soil are likely to be associated by corresponding changes in the soil community [64,65]. As indicated by the dramatic decline in the density and species richness of Collembola and Acari, flooding represented a strong disturbance for soil microarthropods. The community composition of Collembola markedly changed after the flood and this lasted for at least three months. On one hand some species were present at each of the sampling dates, including *Lepidocyrtus lanuginosus*, *Lepidocyrtus cyaneus and Willowsia buski* (Entomobryidae) as well as *Isotoma viridis*, *Parisotoma notabilis* and *Isotomiella minor* (Isotomidae) (see S1 Table). Each of these species had been recorded three weeks after the flood suggesting that they survived the flooding in some of the plots and then recovered quickly confirming their generalistic lifestyle [26]. On the other hand, *Hypogastrura manubrialis*, *Ceratophysella engadinensis* (Hypogastruridae), *Isotomurus fucicolus*, *Proisotoma minuta* (Isotomidae), *Paratullbergia macdougalli* (Tullbergiidae), *Protaphorura armata* (Onychiuridae) and *Sminthurus viridis* (Sminthuridae) were present three weeks after the flood (as single individuals or in few numbers only) but neither three months after the flood nor in 2010 before the flood (S1 Table) suggesting that a number of Collembola species were introduced by the flood. In contrast, *Zygoribatula frisiae* and *Schleloribates initialis* of Oribatida were only present three weeks after the flood. Furthermore, *Oppiella nova*, *Tectocepheus sarekensis*, *Oribatula excavata*, *Rhysotritia ardua* and *Punctoribates punctum* were present in all the dates and most of them quickly increased in density suggesting they are resistant against disturbances and respond in an generalistic way. In fact, these species are known as cosmopolitan generalistic species present in any kind of habitat [31].

## Conclusion

The present study demonstrated that soil microarthropod communities (Collembola and Acari) are affected heavily from summer flooding, but also that they are able to recover quickly. Recovery was based in large on ubiquitous and resistant species surviving the flood and able to form vigorous populations within short period of time (three months). Mobile surface living species were the quickest to recover and in part took advantage of resources made available due to flooding (dead plant material). Widespread microarthropod species with wide habitat niches recovered faster than those with more limited distribution and more narrow niches. Overall, Collembola were more affected by flooding and recovered faster than Acari. In contrast, recovery of community composition of Collembola after flooding was slower than that of Oribatida. These conclusions, however, were based on two sampling events following flooding and long-term studies are needed to uncover the resilience of microarthropod communities to extreme climate events such as flooding.

## Supporting information

S1 Fig Density of Collembola and Acari (individuals m^-2^) in different years (November 2010, July 2013, October 2013, July 2014 and October 2014). (EPS)

S1 Table List of Collembola species, their abbreviations used in Fig 4 and their total numbers at each of the three sampling dates. (PDF)

S2 Table List of Oribatida species, their abbreviations used in Fig 6 and their total numbers at each of the three sampling dates. (PDF)

S3 Table Dataset of Collembola species (individuals in soil cores of 5 cm diameter and 5 cm depth) of November 2010 (before the flood), July 2013 and October 2013 (after the flood) of every plot in the main experiment. (PDF)

S4 Table Dataset of Oribatida species (individuals in soil cores of 5 cm diameter and 5 cm depth) of November 2010 (before the flood), July 2013 and October 2013 (after the flood) of every plot in the main experiment. (PDF)

S5 Table Dataset of total Collembola, Entomobryidae, Isotomidae and Tullbergiidae abundance (individuals found in soil cores of 5 cm diameter and 5 cm depth) of November 2010 (before the flood), July 2013 and October 2013 (after the flood) of every plot in the main experiment. (PDF)

S6 Table Dataset of total Acari, Oribatida, Gamasina, Prostigmata and Astigmata abundance (individuals found in soil cores of 5 cm diameter and 5 cm depth) of November 2010 (before the flood), July 2013 and October 2013 (after the flood) of every plot in the main experiment. (PDF)

## References

[1] Garcia RA, Cabeza M, Rahbek C, Araújo MB. Multiple dimensions of climate change and their implications for biodiversity. Science (80-). 2014;344 10.1126/science.1247579 24786084

[2] IPCC (Intergovernamental Panel on Climate Change). Climate Change 2013: the Physical Science Basis [Internet]. Cambridge University Press, Cambridge, UK; 2013 Available: http://www.ipcc.ch/report/ar5/wg1/

[3] Williamson WM, Wardle DA. The soil microbial community response when plants are subjected to water stress and defoliation disturbance. Appl Soil Ecol. 2007;37: 139–149. 10.1016/j.apsoil.2007.05.003

[4] Schuur EA, Matson PA. Net primary productivity and nutrient cycling across a mesic to wet precipitation gradient in Hawaiian montane forest. Oecologia. 2001;128: 431–442. 10.1007/s004420100671 24549913

[5] Visser EJW, Voesenek LACJ. Acclimation to soil flooding-sensing and signal-transduction. Plant Soil. 2005;254: 197–214. 10.1007/s11104-004-1650-0

[6] Unger IM, Muzika RM, Motavalli PP. The effect of flooding and residue incorporation on soil chemistry, germination and seedling growth. Environ Exp Bot. Elsevier B.V.; 2010;69: 113–120. 10.1016/j.envexpbot.2010.03.005

[7] Blöschl G, Nester T, Komma J, Parajka J, Perdigão RAP. The June 2013 flood in the Upper Danube Basin, and comparisons with the 2002, 1954 and 1899 floods. Hydrol Earth Syst Sci. 2013;17: 5197–5212. 10.5194/hess-17-5197-2013

[8] González Macé O, Steinauer K, Jousset A, Eisenhauer N, Scheu S. Flood-induced changes in soil microbial functions as modified by plant diversity. PLoS One. 2016;11: 1–15. 10.1371/journal.pone.0166349 27870864 PMC5117659

[9] Wagner D, Eisenhauer N, Cesarz S. Plant species richness does not attenuate responses of soil microbial and nematode communities to a flood event. Soil Biol Biochem. Elsevier Ltd; 2015;89: 135–149. 10.1016/j.soilbio.2015.07.001

[10] Wright AJ, Ebeling A, de Kroon H, Roscher C, Weigelt A, Buchmann N, et al Flooding disturbances increase resource availability and productivity but reduce stability in diverse plant communities. Nat Commun. Nature Publishing Group; 2015;6: 6092 10.1038/ncomms7092 25600177

[11] Blankinship JC, Niklaus PA, Hungate BA. A meta-analysis of responses of soil biota to global change. Oecologia. 2011;165: 553–565. 10.1007/s00442-011-1909-0 21274573

[12] Kardol P, Reynolds WN, Norby RJ, Classen AT. Climate change effects on soil microarthropod abundance and community structure. Appl Soil Ecol. Elsevier B.V.; 2011;47: 37–44. 10.1016/j.apsoil.2010.11.001

[13] Wall DH, Bradford MA, St. John MG, Trofymow JA, Behan-Pelletier V, Bignell DE, et al Global decomposition experiment shows soil animal impacts on decomposition are climate-dependent. Glob Chang Biol. 2008;14: 2661–2677. 10.1111/j.1365-2486.2008.01672.x

[14] Bardgett RD, van der Putten WH. Belowground biodiversity and ecosystem functioning. Nature. Nature Publishing Group; 2014;515: 505–511. 10.1038/nature13855 25428498

[15] Filser J. The role of Collembola in carbon and nitrogen cycling in soil. Pedobiologia (Jena). 2002;46: 234–245. 10.1078/0031-4056-00130

[16] Scheu S, Ruess L, Bonkowski M. Interactions between microorganisms and soil micro- and mesofauna Microorganisms in Soils: Roles in Genesis and Functions. Springer Berlin; 2005 pp. 253–275.

[17] Swift MJ, Heal OW, Anderson JM. Decomposition in Terrestrial Ecosystems. Blackwell Science, Oxford, UK; 1979.

[18] Lindberg N, Bengtsson J. Population responses of oribatid mites and collembolans after drought. Appl Soil Ecol. 2005;28: 163–174. 10.1016/j.apsoil.2004.07.003

[19] Makkonen M, Berg MP, van Hal JR, Callaghan T V., Press MC, Aerts R. Traits explain the responses of a sub-arctic Collembola community to climate manipulation. Soil Biol Biochem. Elsevier Ltd; 2011;43: 377–384. 10.1016/j.soilbio.2010.11.004

[20] Kaneko N, McLean MA, Parkinson D. Grazing preference of Onychiurus subtenuis (Collembola) and Oppiella nova (Oribatei) for fungal species inoculated on pine needles. Pedobiologia (Jena). 1995;39: 538–546.

[21] Wallwork JA. Ecology of soil animals. McGraw-Hill, London; 1970.

[22] Maraun M, Salamon JA, Schneider K, Schaefer M, Scheu S. Oribatid mite and collembolan diversity, density and community structure in a moder beech forest (Fagus sylvatica): Effects of mechanical perturbations. Soil Biol Biochem. 2003;35: 1387–1394. 10.1016/S0038-0717(03)00218-9

[23] Siepel H. Life-history tactics of soil microarthropods. Biol Fertil Soils. 1994;18: 263–278. 10.1007/BF00570628

[24] Norton RA. Evolutionary Aspects of Oribatid Mite Life Histories and Consequences for the Origin of the Astigmata In: Houck MA, editor. Mites: Ecological and Evolutionary Analyses of Life-History Patterns. Boston, MA: Springer US; 1994 pp. 99–135. 10.1007/978-1-4615-2389-5_5

[25] Hoffmann AA, Reynolds KT, Nash MA, Weeks AR. A high incidence of parthenogenesis in agricultural pests. Proc R Soc B Biol Sci. 2008;275: 2473–2481. 10.1098/rspb.2008.0685 18647717 PMC2603198

[26] Hopkin SP. Biology of springtails: Collembola (Insecta). Oxford University Press; 2007.

[27] Maraun M, Scheu S. The structure of oribatid mite communities (Acari, Oribatida): patterns, mechanisms and implications for future research. Ecography (Cop). 2000;23: 374–383. 10.1139/x03-284

[28] Maraun M, Martens H, Migge S, Theenhaus A, Scheu S. Adding to “the enigma of soil animal diversity”: Fungal feeders and saprophagous soil invertebrates prefer similar food substrates. Eur J Soil Biol. 2003;39: 85–95. 10.1016/S1164-5563(03)00006-2

[29] Krantz GW, Walter DE. A manual of acarology: Third edition Press TTU, editor. Lubbock, Texas; 2009.

[30] Karg W. Raubmilben, Acari (Acarina), milben Parasitiformes (Anactinochaeta) Cohors Gamasina Leach. Die Tierwelt Deutschlands 59 Teil. Gustav Fischer Verlag: Jena; 1993.

[31] Walter DE, Proctor HC. Mites: ecology, evolution and behaviour. Wallinford,Oxon, UK: CAB International; 1999.

[32] Ponge JF, Dubs F, Gillet S, Sousa JP, Lavelle P. Decreased biodiversity in soil springtail communities: the importance of dispersal and landuse history in heterogeneous landscapes. Soil Biol Biochem. 2006;38: 1158–1161. 10.1016/j.soilbio.2005.09.004

[33] Sabais ACW, Scheu S, Eisenhauer N. Plant species richness drives the density and diversity of Collembola in temperate grassland. Acta Oecologica. 2011;37: 195–202. 10.1016/j.actao.2011.02.002

[34] Wissuwa J, Salamon JA, Frank T. Oribatida (Acari) in grassy arable fallows are more affected by soil properties than habitat age and plant species. Eur J Soil Biol. 2013;59: 8–14. 10.1016/j.ejsobi.2013.08.002 26109839 PMC4461176

[35] Roscher C, Schumacher J, Baade J, Wilcke W, Gleixner G, Weisser WW, et al The role of biodiversity for element cycling and trophic interactions : an experimental approach in a grassland community. Basic Appl Ecol. 2004;5: 107–121.

[36] Macfadyen A. Improved funnel-type extractors for soil arthropods. J Anim Ecol. 1961;30: 171–184.

[37] Weigmann G. Hornmilben (Oribatida) Die Tierwelt Deutschlands. Keltern: Goecke and Evers; 2006.

[38] Fjellberg A. The Collembola of Fennoscandia and Denmark. Part I: Poduromorpha Fauna Entomologica Scandinavica. Brill; 1998.

[39] Fjellberg A. The Collembola of Fennoscandia and Denmark. Part II: Entomobryomorpha and Symphypleona Fauna Entomologica Scandinavica. Brill; 2007.

[40] Faraway JJ. Linear models with R. CRC Press; 2014.

[41] Zuur A, Ieno EN, Smith GM. Analysing Ecological Data. Springer Science & Business Media; 2007.

[42] Roscher C, Schumacher J, Baade J. The role of biodiversity for element cycling and trophic interactions: an experimental approach in a grassland community. Basic Appl Ecol. 2004;5: 107–121. 10.1078/1439-1791-00216

[43] Fox J, Weisberg S. An R companion to applied regression. New York: Sage; 2010.

[44] Schmid B, Hector A, Huston M, Inchausti P, Nijs I, Leadley P, et al The design and analysis of biodiversity experiments. In: Press OU, editor. Biodiversity and ecosystem functioning: synthesis and perspectives. 2002 pp. 61–78.

[45] R Development Core Team. R: A language and environment for statistical computing. R Foundation for Statistical Computing, Vienna, Austria; 2012.

[46] Ter Braak CJF, Smilauer P. Canoco Reference Manual and User’s Guide: Software for Ordination (version 5.0). Ithaca, USA: Microcomputer Power; 2012.

[47] Chauvat M, Zaitsev AS, Wolters V. Successional changes of Collembola and soil microbiota during forest rotation. Oecologia. 2003;137: 269–276. 10.1007/s00442-003-1310-8 12898384

[48] Lindberg N, Bengtsson J. Recovery of forest soil fauna diversity and composition after repeated summer droughts. Oikos. 2006;114: 494–506. 10.1111/j.2006.0030–1299.14396.x

[49] Bardgett R. The Biology of Soil: a Community and Ecosystem Approach. Oxford, UK: Oxford University Press; 2005.

[50] Milcu A, Thebault E, Scheu S, Eisenhauer N. Plant diversity enhances the reliability of belowground processes. Soil Biol Biochem. Elsevier Ltd; 2010;42: 2102–2110. 10.1016/j.soilbio.2010.08.005

[51] Ravenek JM, Bessler H, Engels C, Scherer-Lorenzen M, Gessler A, Gockele A, et al Long-term study of root biomass in a biodiversity experiment reveals shifts in diversity effects over time. Oikos. 2014;123: 1528–1536. 10.1111/oik.01502

[52] Endlweber K, Scheu S. Interactions between mycorrhizal fungi and Collembola: Effects on root structure of competing plant species. Biol Fertil Soils. 2007;43: 741–749. 10.1007/s00374-006-0157-7

[53] Salamon JA, Schaefer M, Alphei J, Schmid B, Scheu S. Effects of plant diversity on Collembola in an experimental grassland ecosystem. Oikos. 2004;106: 51–60. 10.1111/j.0030-1299.2004.12905.x

[54] Socarrás A, Izquierdo I. Evaluation of agroecological systems through biological indicators of the soil quality: edaphic mesofauna. Pastos y Forrajes. 2014;37: 109–114.

[55] Lindberg N. Soil Fauna and Global Change. 2003.

[56] Behan-Pelletier VM. Oribatid mite biodiversity in agrosystems:role for bioindication. Agric Ecosyst Environ. 1999;74: 411–423.

[57] Scheu S, Schulz E. Secondary succession, soil formation and development of a diverse community of oribatids and saprophagous soil macro-invertebrates. Biodivers Conserv. 1996;5: 235–250. 10.1007/BF00055833

[58] Hertzog LR, Ebeling A, Meyer ST, Eisenhauer N, Fischer C, Hildebrandt A, et al High survival of Lasius Niger during summer flooding in a European grassland. PLoS One. 2016;11: 1–12. 10.1371/journal.pone.0152777 27851761 PMC5112897

[59] Dhooria MS. Fundamentals of Applied Acarology. Springer, Singapore; 2016 10.1007/978-981-10-1594-6

[60] Wallwork JA. Desert soil microarthropods in an “r”-selected system. Soil Biology as Related to Land Use Practices. Proceedings of the Seventh International Soil Zoology College ISSS, Washington, DC; 1980. pp. 759–769.

[61] Behan-Pelletier VM. Acari and Collembola biodiversity in Canadian agricultural soils. Can J Soil Sci. 2003;83: 279–288. 10.4141/S01-063

[62] Koehler HH. Predatory mites (Gamasina, Mesostigmata). Agric Ecosyst Environ. 1999;74: 395–410. 10.1016/S0167-8809(99)00045-6

[63] Ruf A, Beck L. The use of predatory soil mites in ecological soil classification and assessment concepts, with perspectives for oribatid mites. Ecotoxicol Environ Saf. 2005;62: 290–299. 10.1016/j.ecoenv.2005.03.029 15979713

[64] Kaufmann R, Fuchs M, Gosterxeier N. The Soil Fauna of an Alpine Glacier Foreland: Colonization and Succession. Arctic, Antarct Alp Res. 2002;34: 242–250.

[65] Bokhorst S, Berg MP, Wardle DA. Micro-arthropod community responses to ecosystem retrogression in boreal forest. Soil Biol Biochem. Elsevier Ltd; 2017;110: 79–86. 10.1016/j.soilbio.2017.03.009

