# Supplementary material for: Response of Collembola and Acari communities to summer flooding in a grassland plant diversity experiment
Source: PLoS One. 2018 Aug 30;13(8):e0202862. doi: 10.1371/journal.pone.0202862 (PMC6117009; doi:10.1371/journal.pone.0202862)
Supplement: S1 Table — (PDF) [file pone.0202862.s002.pdf]

S1 Table List of Collembola species

Family	Species	Abbrev.	November 2010	July 2013	October 2013
Brachystomellidae	*Brachystomella parvula* (Schaeffer, 1896)	Brapar	4	-	19
Entomobryidae	*Entomobrya lanuginosa* (Nicolet, 1842)	Entlan	5	-	-
	Entomobrya multifasciata (Tullberg, 1871)	Entmul	2	-	-
	Heteromurus nitidus (Templeton, 1835)	Hetnit	1	-	1
	Lepidocyrtus cyaneus (Tullberg, 1871)	Lepcya	579	27	1,216
	Lepidocyrtus lanuginosus (Gmelin, 1790)	Leplan	13	6	102
	Pseudosinella alba (Packard, 1873)	Psealb	73	-	5
	Pseudosinella immaculata (Lie-pettersen, 1897)	Pseimm	78	-	-
	Sinella curviseta (Brook, 1882)	Sincur	-	-	59
	Willowsia buski (Lubbock, 1870)	Wilbus	2	6	12
Hypogastruridae	*Ceratophysella denticulata* (Bagnall, 1941)	Cerden	295		22
	Ceratophysella engadinensis (Gisin, 1949)	Cereng	-	1	-
	Ceratophysella succinea (Gisin, 1949)	Cersuc	-	-	9
	Hypogastrura manubrialis (Tullberg, 1869)	Hypman	-	1	-
Isotomidae	*Cryptopygus thermophilus* (Axelson, 1900)	Crythe	-	-	364
	Desoria violacea (Tullberg, 1876)	Desvio	-	-	3
	Folsomia quadrioculata (Tullberg, 1871)	Folqua	-	2	1
	Folsomides parvulus (Stach, 1922)	Folpar	47	-	-
	Isotoma viridis (Bourlet, 1839)	Isovir	57	11	262
	Isotomiella minor (Schaeffer, 1896)	Isomin	49	1	77
	Isotomodes productus (Axelson, 1906)	Isopro	-	-	18
	Isotomurus fucicolus (Reuter, 1891)	Isofuc	-	1	-
	Isotomurus palustris (Müller, 1776)	Isopal	67	-	-
	Parisotoma notabilis (Schaeffer, 1896)	Parnot	400	3	10
	Proisotoma minuta (Tullberg, 1871)	Promin	-	1	-
Katiannidae	*Sminthurinus aureus* (Lubbock, 1862)	Smiaur	7	-	18
	Sminthurinus elegans (Fitch, 1862)	Smiele	1	-	-
	Sminthurinus niger (Lubbock, 1868)	Sminig	45	1	-
Neanuridae	*Friesea mirabilis* (Tullberg, 1871)	Frimir	10	-	1
	Micranurida pygmaea (Borner, 1901)	Micpyg	32	-	16
Neelidae	*Megalothorax minimus* (Willem, 1900)	Megmin	92	-	2
Onychiuridae	*Onychiurus jubilarius* (Gisin, 1957)	Onyjub	162	-	-
	Protaphorura armata (Tullberg, 1869)	Proarm	-	4	-
	Supraphorura furcifera (Börner, 1901)	Supfur	3	-	-
Sminthuridae	*Sminthurus viridis* (Linnaeus, 1758)	Smivir	-	1	-
Sminthurididae	*Sphaeridia pumilis* (Krausbauer, 1898)	Spapum	1	1	-
Tullbergiidae	*Mesaphorura macrochaeta* (Rusek, 1976)	Mesmac	498	-	13
	Metaphorura affinis (Börner, 1903)	Metaff	47	-	-
	Paratullbergia macdougalli (Bagnall, 1936)	Parmac	-	3	-
	Stenaphorurella denisi (Bagnall, 1935)	Steden	288	-	943
