# Supplementary material for: Response of Collembola and Acari communities to summer flooding in a grassland plant diversity experiment
Source: PLoS One. 2018 Aug 30;13(8):e0202862. doi: 10.1371/journal.pone.0202862 (PMC6117009; doi:10.1371/journal.pone.0202862)
Supplement: S2 Table — (PDF) [file pone.0202862.s003.pdf]

S2 Table List of Oribatida species

Family	Species	Abbrev.	November 2010	July 2013	October 2013
Ceratozetidae	*Ceratozetes psammophilus* (Horak, 2000)	Cerpsa	3	-	10
Euphthiracaridae	*Rhysotritia ardua ardua* (Koch, 1841)	Rhyard	39	1	15
Hypochthoniidae	*Hypochthonius rufulus* (Koch, 1835)	Hypruf	-	-	1
Liebstadiidae	*Liebstadia similis* (Michael, 1888)	Liesim	21	-	-
Nothridae	*Nothrus anauniensis* (Canestrini & Fanzago, 1876)	Notana	2	-	-
	Nothrus pratensis (Sellnick, 1928)	Notpra	6	-	-
Oppiidae	*Microppia minus* (Paoli, 1908)	Micmin	-	10	1
	Oppiella nova (Oudemans, 1902)	Opinov	165	1	329
Oribatulidae	*Oribatula excavata* (Berlese, 1916)	Oriexc	246	5	42
	Zygoribatula frisiae (Oudemans, 1900)	Zygfri	-	2	-
Phthiracaridae	*Steganacarus striculus* (Koch, 1836)	Stestr	-	-	1
Protoribatidae	*Protoribates capucinus* (Berlese, 1908)	Procap	-	-	1
Punctoribatidae	*Punctoribates punctum* (Koch, 1839)	Punpun	20	4	28
Scheloribatidae	*Scheloribates (Hemileius) initialis* (Berlese, 1908)	Schini	-	20	-
	Scheloribates laevigatus (Koch, 1835)	Schlae	9	12	74
Tectocepheidae	*Tectocepheus minor* (Berlese, 1903)	Tecmin	2	-	4
	Tectocepheus velatus sarekensis (Trägårdh, 1910)	Tecsar	64	30	61
	Tectocepheus velatus velatus (Michael, 1880)	Tecala	1	-	-
