# Supplementary material for: Response of Collembola and Acari communities to summer flooding in a grassland plant diversity experiment
Source: PLoS One. 2018 Aug 30;13(8):e0202862. doi: 10.1371/journal.pone.0202862 (PMC6117009; doi:10.1371/journal.pone.0202862)
Supplement: S3 Table — (PDF) [file pone.0202862.s004.pdf]

Dataset

Collembola species

Type raw

Unit individuals in soil cores of 5 cm diameter and 5 cm depth

Date	Plot	*Brachystomella parvula*	*Ceratophysella succinea*	*Ceratophysella denticulata*	*Ceratophysella engadinensis*	*Hypogastrura manubrialis*	*Lepidocyrtus cyaneus*	*Lepidocyrtus lanuginosus*	*Entomobrya lanuginosa*
November 2010	B1A01	0	0	0	0	0	10	0	0
November 2010	B1A02	0	0	1	0	0	0	0	0
November 2010	B1A03	0	0	0	0	0	6	1	0
November 2010	B1A04	0	0	0	0	0	9	0	0
November 2010	B1A05	0	0	1	0	0	5	0	0
November 2010	B1A06	4	0	0	0	0	5	0	0
November 2010	B1A07	0	0	7	0	0	1	0	0
November 2010	B1A08	0	0	1	0	0	3	0	3
November 2010	B1A11	0	0	0	0	0	24	0	0
November 2010	B1A12	0	0	0	0	0	5	0	0
November 2010	B1A13	0	0	0	0	0	3	0	0
November 2010	B1A14	0	0	1	0	0	0	0	0
November 2010	B1A15	0	0	0	0	0	0	0	0
November 2010	B1A16	0	0	11	0	0	8	0	0
November 2010	B1A17	0	0	18	0	0	6	0	0
November 2010	B1A18	0	0	0	0	0	0	0	0
November 2010	B1A19	0	0	0	0	0	2	9	0
November 2010	B1A20	0	0	26	0	0	15	0	0
November 2010	B1A21	0	0	0	0	0	10	0	0
November 2010	B1A22	0	0	0	0	0	13	1	0
November 2010	B2A01	0	0	0	0	0	4	0	0
November 2010	B2A02	0	0	0	0	0	0	0	0
November 2010	B2A03	0	0	3	0	0	3	0	0
November 2010	B2A04	0	0	0	0	0	3	0	0
November 2010	B2A05	0	0	4	0	0	8	0	0
November 2010	B2A06	0	0	5	0	0	7	0	0
November 2010	B2A08	0	0	6	0	0	2	0	0
November 2010	B2A09	0	0	7	0	0	4	0	0
November 2010	B2A10	0	0	1	0	0	3	0	0
November 2010	B2A12	0	0	0	0	0	5	0	0
November 2010	B2A13	0	0	0	0	0	7	0	0

Date	Plot	*Brachystomella parvula*	*Ceratophysella succinea*	*Ceratophysella denticulata*	*Ceratophysella engadinensis*	*Hypogastrura manubrialis*	*Lepidocyrtus cyaneus*	*Lepidocyrtus lanuginosus*	*Entomobrya lanuginosa*
November 2010	B2A14	0	0	7	0	0	0	0	0
November 2010	B2A15	0	0	0	0	0	9	0	0
November 2010	B2A16	0	0	5	0	0	6	0	0
November 2010	B2A17	0	0	0	0	0	15	0	0
November 2010	B2A18	0	0	47	0	0	18	0	0
November 2010	B2A19	0	0	1	0	0	0	0	0
November 2010	B2A20	0	0	61	0	0	11	0	0
November 2010	B2A21	0	0	0	0	0	4	0	0
November 2010	B2A22	0	0	0	0	0	0	0	0
November 2010	B3A01	0	0	0	0	0	9	0	0
November 2010	B3A02	0	0	0	0	0	4	0	0
November 2010	B3A03	0	0	0	0	0	0	0	0
November 2010	B3A04	0	0	0	0	0	23	0	0
November 2010	B3A05	0	0	7	0	0	7	0	0
November 2010	B3A06	0	0	0	0	0	20	0	0
November 2010	B3A07	0	0	0	0	0	0	0	0
November 2010	B3A08	0	0	26	0	0	4	0	0
November 2010	B3A09	0	0	22	0	0	6	0	0
November 2010	B3A11	0	0	3	0	0	2	0	0
November 2010	B3A12	0	0	0	0	0	8	0	1
November 2010	B3A13	0	0	0	0	0	10	0	0
November 2010	B3A14	0	0	0	0	0	5	2	0
November 2010	B3A16	0	0	0	0	0	3	0	0
November 2010	B3A17	0	0	0	0	0	3	0	0
November 2010	B3A19	0	0	11	0	0	8	0	0
November 2010	B3A20	0	0	0	0	0	22	0	0
November 2010	B3A21	0	0	0	0	0	0	0	0
November 2010	B3A22	0	0	0	0	0	22	0	0
November 2010	B3A23	0	0	0	0	0	15	0	0
November 2010	B3A24	0	0	0	0	0	36	0	0
November 2010	B4A01	0	0	0	0	0	0	0	0
November 2010	B4A02	0	0	0	0	0	2	0	0
November 2010	B4A04	0	0	0	0	0	27	0	0
November 2010	B4A06	0	0	0	0	0	2	0	0
November 2010	B4A07	0	0	0	0	0	5	0	0
November 2010	B4A08	0	0	0	0	0	3	0	0

Date	Plot	*Brachystomella parvula*	*Ceratophysella succinea*	*Ceratophysella denticulata*	*Ceratophysella engadinensis*	*Hypogastrura manubrialis*	*Lepidocyrtus cyaneus*	*Lepidocyrtus lanuginosus*	*Entomobrya lanuginosa*
November 2010	B4A09	0	0	0	0	0	11	0	0
November 2010	B4A10	0	0	0	0	0	4	0	0
November 2010	B4A11	0	0	0	0	0	8	0	0
November 2010	B4A12	0	0	0	0	0	13	0	0
November 2010	B4A13	0	0	0	0	0	2	0	0
November 2010	B4A14	0	0	0	0	0	13	0	1
November 2010	B4A15	0	0	4	0	0	8	0	0
November 2010	B4A16	0	0	0	0	0	0	0	0
November 2010	B4A17	0	0	9	0	0	10	0	0
November 2010	B4A18	0	0	0	0	0	12	0	0
November 2010	B4A20	0	0	0	0	0	2	0	0
November 2010	B4A21	0	0	0	0	0	1	0	0
November 2010	B4A22	0	0	0	0	0	15	0	0
July 2013	B1A01	0	0	0	0	0	0	0	0
July 2013	B1A02	0	0	0	0	0	0	0	0
July 2013	B1A03	0	0	0	0	0	0	0	0
July 2013	B1A04	0	0	0	0	0	0	0	0
July 2013	B1A05	0	0	0	0	0	0	0	0
July 2013	B1A06	0	0	0	0	0	0	0	0
July 2013	B1A07	0	0	0	0	0	0	0	0
July 2013	B1A08	0	0	0	0	0	0	0	0
July 2013	B1A11	0	0	0	0	0	0	0	0
July 2013	B1A12	0	0	0	0	0	0	0	0
July 2013	B1A13	0	0	0	0	0	0	0	0
July 2013	B1A14	0	0	0	0	0	0	0	0
July 2013	B1A15	0	0	0	0	0	0	0	0
July 2013	B1A16	0	0	0	0	0	0	0	0
July 2013	B1A17	0	0	0	0	0	0	0	0
July 2013	B1A18	0	0	0	0	0	0	0	0
July 2013	B1A19	0	0	0	0	0	0	0	0
July 2013	B1A20	0	0	0	0	0	0	0	0
July 2013	B1A21	0	0	0	0	0	0	0	0
July 2013	B1A22	0	0	0	0	0	0	0	0
July 2013	B2A01	0	0	0	0	0	0	0	0
July 2013	B2A02	0	0	0	0	0	0	0	0
July 2013	B2A03	0	0	0	0	0	0	0	0

Date	Plot	*Brachystomella parvula*	*Ceratophysella succinea*	*Ceratophysella denticulata*	*Ceratophysella engadinensis*	*Hypogastrura manubrialis*	*Lepidocyrtus cyaneus*	*Lepidocyrtus lanuginosus*	*Entomobrya lanuginosa*
July 2013	B2A04	0	0	0	0	0	0	0	0
July 2013	B2A05	0	0	0	0	0	0	0	0
July 2013	B2A06	0	0	0	0	0	1	0	0
July 2013	B2A08	0	0	0	0	0	0	0	0
July 2013	B2A09	0	0	0	0	0	0	0	0
July 2013	B2A10	0	0	0	0	0	0	0	0
July 2013	B2A12	0	0	0	0	0	6	2	0
July 2013	B2A13	0	0	0	0	0	0	0	0
July 2013	B2A14	0	0	0	0	0	0	0	0
July 2013	B2A15	0	0	0	0	0	0	0	0
July 2013	B2A16	0	0	0	1	1	5	2	0
July 2013	B2A17	0	0	0	0	0	0	0	0
July 2013	B2A18	0	0	0	0	0	0	0	0
July 2013	B2A19	0	0	0	0	0	0	0	0
July 2013	B2A20	0	0	0	0	0	0	0	0
July 2013	B2A21	0	0	0	0	0	0	0	0
July 2013	B2A22	0	0	0	0	0	0	0	0
July 2013	B3A01	0	0	0	0	0	0	0	0
July 2013	B3A02	0	0	0	0	0	0	0	0
July 2013	B3A03	0	0	0	0	0	0	0	0
July 2013	B3A04	0	0	0	0	0	0	0	0
July 2013	B3A05	0	0	0	0	0	0	0	0
July 2013	B3A06	0	0	0	0	0	0	0	0
July 2013	B3A07	0	0	0	0	0	0	0	0
July 2013	B3A08	0	0	0	0	0	0	0	0
July 2013	B3A09	0	0	0	0	0	0	0	0
July 2013	B3A11	0	0	0	0	0	0	0	0
July 2013	B3A12	0	0	0	0	0	0	0	0
July 2013	B3A13	0	0	0	0	0	2	0	0
July 2013	B3A14	0	0	0	0	0	0	0	0
July 2013	B3A16	0	0	0	0	0	0	0	0
July 2013	B3A17	0	0	0	0	0	0	0	0
July 2013	B3A19	0	0	0	0	0	0	0	0
July 2013	B3A20	0	0	0	0	0	0	0	0
July 2013	B3A21	0	0	0	0	0	0	0	0
July 2013	B3A22	0	0	0	0	0	0	0	0

Date	Plot	*Brachystomella parvula*	*Ceratophysella succinea*	*Ceratophysella denticulata*	*Ceratophysella engadinensis*	*Hypogastrura manubrialis*	*Lepidocyrtus cyaneus*	*Lepidocyrtus lanuginosus*	*Entomobrya lanuginosa*
July 2013	B3A23	0	0	0	0	0	0	0	0
July 2013	B3A24	0	0	0	0	0	0	0	0
July 2013	B4A01	0	0	0	0	0	0	0	0
July 2013	B4A02	0	0	0	0	0	0	0	0
July 2013	B4A04	0	0	0	0	0	0	0	0
July 2013	B4A06	0	0	0	0	0	0	0	0
July 2013	B4A07	0	0	0	0	0	0	0	0
July 2013	B4A08	0	0	0	0	0	0	0	0
July 2013	B4A09	0	0	0	0	0	10	2	0
July 2013	B4A10	0	0	0	0	0	1	0	0
July 2013	B4A11	0	0	0	0	0	0	0	0
July 2013	B4A12	0	0	0	0	0	0	0	0
July 2013	B4A13	0	0	0	0	0	0	0	0
July 2013	B4A14	0	0	0	0	0	0	0	0
July 2013	B4A15	0	0	0	0	0	0	0	0
July 2013	B4A16	0	0	0	0	0	0	0	0
July 2013	B4A17	0	0	0	0	0	0	0	0
July 2013	B4A18	0	0	0	0	0	1	0	0
July 2013	B4A20	0	0	0	0	0	0	0	0
July 2013	B4A21	0	0	0	0	0	1	0	0
July 2013	B4A22	0	0	0	0	0	0	0	0
October 2013	B1A01	1	0	0	0	0	22	0	0
October 2013	B1A02	0	0	0	0	0	4	0	0
October 2013	B1A03	0	0	0	0	0	2	0	0
October 2013	B1A04	0	0	0	0	0	14	0	0
October 2013	B1A05	0	0	0	0	0	0	0	0
October 2013	B1A06	0	0	0	0	0	7	3	0
October 2013	B1A07	2	0	1	0	0	40	7	0
October 2013	B1A08	0	0	0	0	0	7	0	0
October 2013	B1A11	0	0	0	0	0	2	0	0
October 2013	B1A12	0	0	0	0	0	1	0	0
October 2013	B1A13	0	0	0	0	0	0	0	0
October 2013	B1A14	0	0	1	0	0	10	0	0
October 2013	B1A15	0	0	0	0	0	3	0	0
October 2013	B1A16	0	1	0	0	0	5	0	0
October 2013	B1A17	0	0	0	0	0	7	0	0

Date	Plot	*Brachystomella parvula*	*Ceratophysella succinea*	*Ceratophysella denticulata*	*Ceratophysella engadinensis*	*Hypogastrura manubrialis*	*Lepidocyrtus cyaneus*	*Lepidocyrtus lanuginosus*	*Entomobrya lanuginosa*
October 2013	B1A18	0	0	0	0	0	19	0	0
October 2013	B1A19	1	0	0	0	0	7	0	0
October 2013	B1A20	0	3	0	0	0	3	0	0
October 2013	B1A21	0	0	0	0	0	8	1	0
October 2013	B1A22	0	0	0	0	0	6	0	0
October 2013	B2A01	0	2	0	0	0	6	2	0
October 2013	B2A02	7	1	4	0	0	57	0	0
October 2013	B2A03	0	0	0	0	0	7	0	0
October 2013	B2A04	0	0	0	0	0	3	0	0
October 2013	B2A05	0	0	0	0	0	1	0	0
October 2013	B2A06	0	0	0	0	0	1	0	0
October 2013	B2A08	0	0	0	0	0	4	1	0
October 2013	B2A09	0	0	1	0	0	2	0	0
October 2013	B2A10	0	0	0	0	0	8	0	0
October 2013	B2A12	0	0	0	0	0	15	0	0
October 2013	B2A13	0	0	0	0	0	2	0	0
October 2013	B2A14	0	0	0	0	0	4	0	0
October 2013	B2A15	0	0	0	0	0	19	0	0
October 2013	B2A16	0	0	0	0	0	39	1	0
October 2013	B2A17	0	0	0	0	0	12	0	0
October 2013	B2A18	0	0	0	0	0	0	0	0
October 2013	B2A19	0	0	0	0	0	39	0	0
October 2013	B2A20	0	0	0	0	0	1	1	0
October 2013	B2A21	0	0	0	0	0	8	1	0
October 2013	B2A22	0	0	0	0	0	72	18	0
October 2013	B3A01	0	0	0	0	0	62	4	0
October 2013	B3A02	0	0	0	0	0	22	5	0
October 2013	B3A03	0	0	0	0	0	1	2	0
October 2013	B3A04	0	0	0	0	0	1	1	0
October 2013	B3A05	0	0	0	0	0	27	3	0
October 2013	B3A06	0	0	0	0	0	20	4	0
October 2013	B3A07	0	0	0	0	0	101	8	0
October 2013	B3A08	0	0	0	0	0	3	0	0
October 2013	B3A09	0	0	0	0	0	2	0	0
October 2013	B3A11	0	0	0	0	0	9	0	0
October 2013	B3A12	0	0	0	0	0	37	0	0

Date	Plot	*Brachystomella parvula*	*Ceratophysella succinea*	*Ceratophysella denticulata*	*Ceratophysella engadinensis*	*Hypogastrura manubrialis*	*Lepidocyrtus cyaneus*	*Lepidocyrtus lanuginosus*	*Entomobrya lanuginosa*
October 2013	B3A13	0	0	0	0	0	6	0	0
October 2013	B3A14	0	1	0	0	0	28	4	0
October 2013	B3A16	0	0	0	0	0	9	0	0
October 2013	B3A17	0	0	0	0	0	20	5	0
October 2013	B3A19	0	0	0	0	0	70	0	0
October 2013	B3A20	0	0	0	0	0	1	2	0
October 2013	B3A21	0	0	0	0	0	4	0	0
October 2013	B3A22	0	0	0	0	0	50	8	0
October 2013	B3A23	0	0	0	0	0	4	1	0
October 2013	B3A24	0	0	0	0	0	6	4	0
October 2013	B4A01	0	0	0	0	0	16	0	0
October 2013	B4A02	0	0	1	0	0	51	2	0
October 2013	B4A04	0	0	0	0	0	3	0	0
October 2013	B4A06	0	0	0	0	0	12	1	0
October 2013	B4A07	0	0	0	0	0	22	4	0
October 2013	B4A08	1	0	0	0	0	80	4	0
October 2013	B4A09	0	0	13	0	0	5	0	0
October 2013	B4A10	1	0	0	0	0	0	0	0
October 2013	B4A11	0	0	0	0	0	2	0	0
October 2013	B4A12	0	0	0	0	0	3	0	0
October 2013	B4A13	0	0	0	0	0	0	0	0
October 2013	B4A14	0	0	1	0	0	20	2	0
October 2013	B4A15	5	0	0	0	0	25	0	0
October 2013	B4A16	0	0	0	0	0	11	0	0
October 2013	B4A17	0	0	0	0	0	4	0	0
October 2013	B4A18	1	0	0	0	0	4	3	0
October 2013	B4A20	0	0	0	0	0	2	0	0
October 2013	B4A21	0	1	0	0	0	0	0	0
October 2013	B4A22	0	0	0	0	0	6	0	0

Dataset

Collembola species
Type raw
Unit

Date	Plot	*Entomobrya multifusciata*	*Heteromurus nitidus*	*Pseudosinella alba*	*Sinella curviseta*	*Willosia buski*	*Pseudosinella immaculata*	*Frisea mirabilis*	*Micranurida pygmea*	*Folsomides parvulus*	*Isotoma viridis*
November 2010	B1A01	0	0	0	0	0	0	0	0	0	1
November 2010	B1A02	0	0	0	0	0	1	0	3	0	0
November 2010	B1A03	0	0	2	0	0	5	0	0	0	1
November 2010	B1A04	0	0	1	0	0	0	0	0	0	1
November 2010	B1A05	0	0	0	0	0	0	0	1	1	0
November 2010	B1A06	0	0	0	0	0	0	0	0	0	0
November 2010	B1A07	0	0	0	0	0	0	0	0	0	0
November 2010	B1A08	0	0	0	0	0	0	0	0	1	0
November 2010	B1A11	0	0	1	0	0	1	0	1	0	1
November 2010	B1A12	0	0	1	0	0	4	0	0	0	0
November 2010	B1A13	0	0	0	0	0	0	0	0	1	2
November 2010	B1A14	0	0	0	0	0	0	0	0	0	0
November 2010	B1A15	0	0	0	0	0	0	0	0	0	0
November 2010	B1A16	0	0	1	0	0	0	1	0	0	0
November 2010	B1A17	0	0	2	0	0	2	0	0	1	0
November 2010	B1A18	0	0	0	0	0	0	0	0	0	0
November 2010	B1A19	0	0	0	0	0	0	0	0	0	1
November 2010	B1A20	0	0	3	0	0	0	0	0	0	9
November 2010	B1A21	0	0	3	0	0	1	0	0	0	0
November 2010	B1A22	0	0	1	0	0	1	0	0	0	1
November 2010	B2A01	1	0	0	0	0	2	0	0	0	0
November 2010	B2A02	0	0	0	0	0	0	0	0	0	0
November 2010	B2A03	0	0	3	0	0	0	0	0	0	5
November 2010	B2A04	0	0	2	0	0	0	0	0	0	0
November 2010	B2A05	0	0	0	0	0	0	1	0	0	1
November 2010	B2A06	0	0	2	0	0	3	0	2	0	0
November 2010	B2A08	0	0	1	0	0	1	0	0	0	0
November 2010	B2A09	0	0	0	0	0	0	0	0	0	0
November 2010	B2A10	0	0	1	0	0	0	0	7	10	0
November 2010	B2A12	0	0	0	0	0	1	0	0	0	0
November 2010	B2A13	0	0	0	0	0	0	0	0	0	0

Date	Plot	*Entomobrya multifusciata*	*Heteromurus nitidus*	*Pseudosinella alba*	*Sinella curviseta*	*Willosia buski*	*Pseudosinella immaculata*	*Frisea mirabilis*	*Micranurida pygmea*	*Folsomides parvulus*	*Isotoma viridis*
November 2010	B2A14	0	0	0	0	0	0	1	1	0	0
November 2010	B2A15	0	0	2	0	0	0	0	0	0	0
November 2010	B2A16	0	1	0	0	0	1	0	0	0	0
November 2010	B2A17	0	0	0	0	0	0	0	0	0	1
November 2010	B2A18	0	0	2	0	0	2	0	3	0	0
November 2010	B2A19	0	0	0	0	0	0	0	0	0	1
November 2010	B2A20	0	0	0	0	0	0	0	0	2	0
November 2010	B2A21	0	0	2	0	0	1	0	0	2	4
November 2010	B2A22	0	0	0	0	0	0	0	0	0	4
November 2010	B3A01	0	0	0	0	0	0	0	0	0	1
November 2010	B3A02	0	0	0	0	0	0	0	0	1	0
November 2010	B3A03	0	0	0	0	0	0	0	0	0	0
November 2010	B3A04	0	0	1	0	0	2	4	8	5	1
November 2010	B3A05	0	0	2	0	0	7	0	0	0	0
November 2010	B3A06	0	0	3	0	0	0	2	0	0	0
November 2010	B3A07	0	0	0	0	0	0	0	0	0	1
November 2010	B3A08	0	0	0	0	0	0	0	0	0	0
November 2010	B3A09	0	0	1	0	0	1	0	0	0	0
November 2010	B3A11	0	0	0	0	0	1	0	0	0	5
November 2010	B3A12	0	0	0	0	0	0	0	0	0	2
November 2010	B3A13	0	0	4	0	0	0	0	0	0	0
November 2010	B3A14	0	0	2	0	2	0	0	0	0	2
November 2010	B3A16	0	0	2	0	0	0	0	0	0	0
November 2010	B3A17	0	0	0	0	0	0	0	0	0	0
November 2010	B3A19	0	0	0	0	0	0	1	4	2	0
November 2010	B3A20	0	0	4	0	0	2	0	0	0	1
November 2010	B3A21	0	0	0	0	0	0	0	0	0	2
November 2010	B3A22	0	0	1	0	0	0	0	0	0	1
November 2010	B3A23	0	0	4	0	0	6	0	0	0	0
November 2010	B3A24	0	0	2	0	0	5	0	0	0	1
November 2010	B4A01	0	0	13	0	0	0	0	1	1	0
November 2010	B4A02	0	0	0	0	0	0	0	0	0	0
November 2010	B4A04	0	0	1	0	0	0	0	1	0	1
November 2010	B4A06	0	0	0	0	0	0	0	0	0	0
November 2010	B4A07	0	0	1	0	0	3	0	0	1	2
November 2010	B4A08	0	0	0	0	0	0	0	0	6	0

Date	Plot	*Entomobrya multifusciata*	*Heteromurus nitidus*	*Pseudosinella alba*	*Sinella curviseta*	*Willosia buski*	*Pseudosinella immaculata*	*Frisea mirabilis*	*Micranurida pygmea*	*Folsomides parvulus*	*Isotoma viridis*
November 2010	B4A09	0	0	0	0	0	2	0	0	0	1
November 2010	B4A10	0	0	0	0	0	5	0	0	0	0
November 2010	B4A11	0	0	0	0	0	9	0	0	6	0
November 2010	B4A12	0	0	0	0	0	0	0	0	6	0
November 2010	B4A13	0	0	0	0	0	0	0	0	0	1
November 2010	B4A14	0	0	0	0	0	0	0	0	1	1
November 2010	B4A15	0	0	0	0	0	2	0	0	0	0
November 2010	B4A16	0	0	0	0	0	3	0	0	0	0
November 2010	B4A17	0	0	0	0	0	0	0	0	0	0
November 2010	B4A18	1	0	2	0	0	4	0	0	0	0
November 2010	B4A20	0	0	0	0	0	0	0	0	0	1
November 2010	B4A21	0	0	0	0	0	0	0	0	0	0
November 2010	B4A22	0	0	0	0	0	0	0	0	0	0
July 2013	B1A01	0	0	0	0	0	0	0	0	0	0
July 2013	B1A02	0	0	0	0	0	0	0	0	0	0
July 2013	B1A03	0	0	0	0	0	0	0	0	0	0
July 2013	B1A04	0	0	0	0	0	0	0	0	0	0
July 2013	B1A05	0	0	0	0	0	0	0	0	0	0
July 2013	B1A06	0	0	0	0	0	0	0	0	0	0
July 2013	B1A07	0	0	0	0	0	0	0	0	0	0
July 2013	B1A08	0	0	0	0	0	0	0	0	0	0
July 2013	B1A11	0	0	0	0	0	0	0	0	0	1
July 2013	B1A12	0	0	0	0	0	0	0	0	0	1
July 2013	B1A13	0	0	0	0	0	0	0	0	0	0
July 2013	B1A14	0	0	0	0	0	0	0	0	0	0
July 2013	B1A15	0	0	0	0	0	0	0	0	0	0
July 2013	B1A16	0	0	0	0	0	0	0	0	0	0
July 2013	B1A17	0	0	0	0	0	0	0	0	0	0
July 2013	B1A18	0	0	0	0	0	0	0	0	0	0
July 2013	B1A19	0	0	0	0	0	0	0	0	0	0
July 2013	B1A20	0	0	0	0	0	0	0	0	0	0
July 2013	B1A21	0	0	0	0	0	0	0	0	0	0
July 2013	B1A22	0	0	0	0	0	0	0	0	0	0
July 2013	B2A01	0	0	0	0	0	0	0	0	0	0
July 2013	B2A02	0	0	0	0	0	0	0	0	0	0
July 2013	B2A03	0	0	0	0	0	0	0	0	0	0

Date	Plot	*Entomobrya multifusciata*	*Heteromurus nitidus*	*Pseudosinella alba*	*Sinella curviseta*	*Willosia buski*	*Pseudosinella immaculata*	*Frisea mirabilis*	*Micranurida pygmea*	*Folsomides parvulus*	*Isotoma viridis*
July 2013	B2A04	0	0	0	0	0	0	0	0	0	0
July 2013	B2A05	0	0	0	0	0	0	0	0	0	0
July 2013	B2A06	0	0	0	0	0	0	0	0	0	1
July 2013	B2A08	0	0	0	0	0	0	0	0	0	0
July 2013	B2A09	0	0	0	0	0	0	0	0	0	0
July 2013	B2A10	0	0	0	0	0	0	0	0	0	0
July 2013	B2A12	0	0	0	0	0	0	0	0	0	1
July 2013	B2A13	0	0	0	0	0	0	0	0	0	0
July 2013	B2A14	0	0	0	0	0	0	0	0	0	0
July 2013	B2A15	0	0	0	0	0	0	0	0	0	0
July 2013	B2A16	0	0	0	0	0	0	0	0	0	4
July 2013	B2A17	0	0	0	0	0	0	0	0	0	0
July 2013	B2A18	0	0	0	0	0	0	0	0	0	0
July 2013	B2A19	0	0	0	0	0	0	0	0	0	0
July 2013	B2A20	0	0	0	0	0	0	0	0	0	0
July 2013	B2A21	0	0	0	0	1	0	0	0	0	0
July 2013	B2A22	0	0	0	0	0	0	0	0	0	0
July 2013	B3A01	0	0	0	0	0	0	0	0	0	0
July 2013	B3A02	0	0	0	0	0	0	0	0	0	0
July 2013	B3A03	0	0	0	0	0	0	0	0	0	0
July 2013	B3A04	0	0	0	0	0	0	0	0	0	0
July 2013	B3A05	0	0	0	0	0	0	0	0	0	0
July 2013	B3A06	0	0	0	0	0	0	0	0	0	0
July 2013	B3A07	0	0	0	0	0	0	0	0	0	0
July 2013	B3A08	0	0	0	0	0	0	0	0	0	0
July 2013	B3A09	0	0	0	0	2	0	0	0	0	0
July 2013	B3A11	0	0	0	0	0	0	0	0	0	0
July 2013	B3A12	0	0	0	0	0	0	0	0	0	0
July 2013	B3A13	0	0	0	0	0	0	0	0	0	0
July 2013	B3A14	0	0	0	0	0	0	0	0	0	0
July 2013	B3A16	0	0	0	0	0	0	0	0	0	0
July 2013	B3A17	0	0	0	0	0	0	0	0	0	0
July 2013	B3A19	0	0	0	0	0	0	0	0	0	0
July 2013	B3A20	0	0	0	0	0	0	0	0	0	0
July 2013	B3A21	0	0	0	0	0	0	0	0	0	0
July 2013	B3A22	0	0	0	0	0	0	0	0	0	1

Date	Plot	*Entomobrya multifusciata*	*Heteromurus nitidus*	*Pseudosinella alba*	*Sinella curviseta*	*Willosia buski*	*Pseudosinella immaculata*	*Frisea mirabilis*	*Micranurida pygmea*	*Folsomides parvulus*	*Isotoma viridis*
July 2013	B3A23	0	0	0	0	0	0	0	0	0	0
July 2013	B3A24	0	0	0	0	0	0	0	0	0	0
July 2013	B4A01	0	0	0	0	0	0	0	0	0	0
July 2013	B4A02	0	0	0	0	0	0	0	0	0	0
July 2013	B4A04	0	0	0	0	1	0	0	0	0	0
July 2013	B4A06	0	0	0	0	0	0	0	0	0	1
July 2013	B4A07	0	0	0	0	0	0	0	0	0	0
July 2013	B4A08	0	0	0	0	0	0	0	0	0	0
July 2013	B4A09	0	0	0	0	0	0	0	0	0	1
July 2013	B4A10	0	0	0	0	0	0	0	0	0	0
July 2013	B4A11	0	0	0	0	0	0	0	0	0	0
July 2013	B4A12	0	0	0	0	0	0	0	0	0	0
July 2013	B4A13	0	0	0	0	0	0	0	0	0	0
July 2013	B4A14	0	0	0	0	0	0	0	0	0	0
July 2013	B4A15	0	0	0	0	0	0	0	0	0	0
July 2013	B4A16	0	0	0	0	0	0	0	0	0	0
July 2013	B4A17	0	0	0	0	1	0	0	0	0	0
July 2013	B4A18	0	0	0	0	1	0	0	0	0	0
July 2013	B4A20	0	0	0	0	0	0	0	0	0	0
July 2013	B4A21	0	0	0	0	0	0	0	0	0	0
July 2013	B4A22	0	0	0	0	0	0	0	0	0	0
October 2013	B1A01	0	0	2	0	0	0	0	0	0	1
October 2013	B1A02	0	0	2	0	0	0	0	0	0	0
October 2013	B1A03	0	0	0	0	0	0	0	1	0	0
October 2013	B1A04	0	0	0	0	0	0	0	0	0	0
October 2013	B1A05	0	0	0	0	0	0	0	0	0	0
October 2013	B1A06	0	0	0	0	0	0	0	6	0	0
October 2013	B1A07	0	0	1	7	0	0	0	1	0	5
October 2013	B1A08	0	0	0	0	0	0	0	0	0	1
October 2013	B1A11	0	0	0	0	0	0	0	0	0	0
October 2013	B1A12	0	0	0	2	0	0	0	0	0	0
October 2013	B1A13	0	0	0	0	0	0	0	0	0	0
October 2013	B1A14	0	0	0	0	0	0	0	2	0	1
October 2013	B1A15	0	0	0	1	0	0	0	0	0	0
October 2013	B1A16	0	0	0	0	0	0	0	0	0	0
October 2013	B1A17	0	0	0	0	0	0	0	0	0	0

Date	Plot	*Entomobrya multifusciata*	*Heteromurus nitidus*	*Pseudosinella alba*	*Sinella curviseta*	*Willosia buski*	*Pseudosinella immaculata*	*Frisea mirabilis*	*Micranurida pygmea*	*Folsomides parvulus*	*Isotoma viridis*
October 2013	B1A18	0	0	0	0	0	0	0	0	0	1
October 2013	B1A19	0	0	0	1	0	0	0	1	0	0
October 2013	B1A20	0	0	0	1	0	0	0	0	0	0
October 2013	B1A21	0	0	0	0	0	0	0	0	0	1
October 2013	B1A22	0	0	0	0	0	0	0	0	0	0
October 2013	B2A01	0	0	0	1	0	0	0	0	0	1
October 2013	B2A02	0	0	0	0	0	0	0	3	0	2
October 2013	B2A03	0	0	0	0	0	0	0	0	0	0
October 2013	B2A04	0	0	0	1	0	0	0	0	0	0
October 2013	B2A05	0	0	0	0	0	0	1	0	0	12
October 2013	B2A06	0	0	0	3	0	0	0	0	0	0
October 2013	B2A08	0	0	0	1	0	0	0	0	0	4
October 2013	B2A09	0	0	0	0	0	0	0	0	0	0
October 2013	B2A10	0	0	0	0	0	0	0	0	0	0
October 2013	B2A12	0	0	0	1	0	0	0	0	0	0
October 2013	B2A13	0	0	0	2	0	0	0	0	0	1
October 2013	B2A14	0	0	0	1	0	0	0	0	0	4
October 2013	B2A15	0	0	0	2	2	0	0	0	0	0
October 2013	B2A16	0	0	0	3	0	0	0	0	0	32
October 2013	B2A17	0	0	0	0	1	0	0	0	0	3
October 2013	B2A18	0	0	0	0	1	0	0	0	0	21
October 2013	B2A19	0	0	0	0	0	0	0	0	0	30
October 2013	B2A20	0	0	0	0	0	0	0	0	0	1
October 2013	B2A21	0	0	0	3	0	0	0	0	0	2
October 2013	B2A22	0	0	0	0	0	0	0	0	0	20
October 2013	B3A01	0	0	0	0	0	0	0	0	0	2
October 2013	B3A02	0	0	0	3	0	0	0	0	0	0
October 2013	B3A03	0	0	0	0	0	0	0	0	0	0
October 2013	B3A04	0	0	0	0	0	0	0	0	0	0
October 2013	B3A05	0	0	0	0	0	0	0	0	0	0
October 2013	B3A06	0	0	0	0	0	0	0	0	0	0
October 2013	B3A07	0	0	0	0	0	0	0	0	0	6
October 2013	B3A08	0	0	0	0	0	0	0	0	0	5
October 2013	B3A09	0	0	0	2	0	0	0	0	0	0
October 2013	B3A11	0	0	0	0	0	0	0	0	0	4
October 2013	B3A12	0	0	0	0	5	0	0	0	0	16

Date	Plot	*Entomobrya multifusciata*	*Heteromurus nitidus*	*Pseudosinella alba*	*Sinella curviseta*	*Willosia buski*	*Pseudosinella immaculata*	*Frisea mirabilis*	*Micranurida pygmea*	*Folsomides parvulus*	*Isotoma viridis*
October 2013	B3A13	0	0	0	0	1	0	0	0	0	0
October 2013	B3A14	0	0	0	0	0	0	0	0	0	12
October 2013	B3A16	0	0	0	0	0	0	0	0	0	0
October 2013	B3A17	0	0	0	0	0	0	0	0	0	8
October 2013	B3A19	0	0	0	0	1	0	0	0	0	0
October 2013	B3A20	0	0	0	0	0	0	0	0	0	1
October 2013	B3A21	0	0	0	0	0	0	0	0	0	0
October 2013	B3A22	0	0	0	0	0	0	0	0	0	0
October 2013	B3A23	0	0	0	3	0	0	0	0	0	1
October 2013	B3A24	0	0	0	0	0	0	0	0	0	3
October 2013	B4A01	0	0	0	0	0	0	0	0	0	12
October 2013	B4A02	0	0	0	0	0	0	0	0	0	2
October 2013	B4A04	0	0	0	0	0	0	0	0	0	4
October 2013	B4A06	0	0	0	2	0	0	0	0	0	9
October 2013	B4A07	0	0	0	5	0	0	0	0	0	0
October 2013	B4A08	0	0	0	0	1	0	0	0	0	17
October 2013	B4A09	0	0	0	0	0	0	0	0	0	1
October 2013	B4A10	0	0	0	6	0	0	0	2	0	0
October 2013	B4A11	0	0	0	0	0	0	0	0	0	0
October 2013	B4A12	0	0	0	3	0	0	0	0	0	0
October 2013	B4A13	0	0	0	0	0	0	0	0	0	2
October 2013	B4A14	0	0	0	0	0	0	0	0	0	2
October 2013	B4A15	0	0	0	5	0	0	0	0	0	4
October 2013	B4A16	0	0	0	0	0	0	0	0	0	1
October 2013	B4A17	0	0	0	0	0	0	0	0	0	3
October 2013	B4A18	0	0	0	0	0	0	0	0	0	2
October 2013	B4A20	0	0	0	0	0	0	0	0	0	1
October 2013	B4A21	0	1	0	0	0	0	0	0	0	0
October 2013	B4A22	0	0	0	0	0	0	0	0	0	1

Dataset

Collembola species
Type raw
Unit

Date	Plot	*Cryptopygus thermophilus*	*Desoria violacea*	*Isotomiella minor*	*Isotomodes productus*	*Folsomia quadrioculata*	*Isotumurus palustris*	*Parisotoma notabilis*	*Isotomurus fucicolus*	*Proisotoma minuta*
November 2010	B1A01	0	0	0	0	0	0	0	0	0
November 2010	B1A02	0	0	17	0	0	0	3	0	0
November 2010	B1A03	0	0	3	0	0	0	3	0	0
November 2010	B1A04	0	0	0	0	0	1	32	0	0
November 2010	B1A05	0	0	0	0	0	5	0	0	0
November 2010	B1A06	0	0	1	0	0	0	21	0	0
November 2010	B1A07	0	0	0	0	0	0	0	0	0
November 2010	B1A08	0	0	0	0	0	0	7	0	0
November 2010	B1A11	0	0	0	0	0	1	8	0	0
November 2010	B1A12	0	0	0	0	0	0	1	0	0
November 2010	B1A13	0	0	0	0	0	15	1	0	0
November 2010	B1A14	0	0	0	0	0	0	2	0	0
November 2010	B1A15	0	0	0	0	0	0	0	0	0
November 2010	B1A16	0	0	0	0	0	0	1	0	0
November 2010	B1A17	0	0	0	0	0	0	3	0	0
November 2010	B1A18	0	0	0	0	0	0	0	0	0
November 2010	B1A19	0	0	0	0	0	0	8	0	0
November 2010	B1A20	0	0	0	0	0	0	3	0	0
November 2010	B1A21	0	0	0	0	0	5	1	0	0
November 2010	B1A22	0	0	0	0	0	0	0	0	0
November 2010	B2A01	0	0	1	0	0	0	0	0	0
November 2010	B2A02	0	0	0	0	0	0	0	0	0
November 2010	B2A03	0	0	0	0	0	0	0	0	0
November 2010	B2A04	0	0	0	0	0	0	0	0	0
November 2010	B2A05	0	0	0	0	0	0	0	0	0
November 2010	B2A06	0	0	1	0	0	10	12	0	0
November 2010	B2A08	0	0	2	0	0	0	10	0	0
November 2010	B2A09	0	0	0	0	0	0	6	0	0
November 2010	B2A10	0	0	0	0	0	0	82	0	0
November 2010	B2A12	0	0	0	0	0	0	1	0	0
November 2010	B2A13	0	0	0	0	0	0	0	0	0

Date	Plot	*Cryptopygus thermophilus*	*Desoria violacea*	*Isotomiella minor*	*Isotomodes productus*	*Folsomia quadrioculata*	*Isotumurus palustris*	*Parisotoma notabilis*	*Isotomurus fucicolus*	*Proisotoma minuta*
November 2010	B2A14	0	0	2	0	0	0	0	0	0
November 2010	B2A15	0	0	0	0	0	0	0	0	0
November 2010	B2A16	0	0	0	0	0	0	1	0	0
November 2010	B2A17	0	0	0	0	0	0	0	0	0
November 2010	B2A18	0	0	0	0	0	0	11	0	0
November 2010	B2A19	0	0	0	0	0	0	0	0	0
November 2010	B2A20	0	0	0	0	0	0	7	0	0
November 2010	B2A21	0	0	0	0	0	0	6	0	0
November 2010	B2A22	0	0	0	0	0	0	0	0	0
November 2010	B3A01	0	0	0	0	0	0	0	0	0
November 2010	B3A02	0	0	0	0	0	0	2	0	0
November 2010	B3A03	0	0	0	0	0	0	0	0	0
November 2010	B3A04	0	0	0	0	0	0	20	0	0
November 2010	B3A05	0	0	0	0	0	0	5	0	0
November 2010	B3A06	0	0	0	0	0	0	4	0	0
November 2010	B3A07	0	0	0	0	0	0	0	0	0
November 2010	B3A08	0	0	2	0	0	0	7	0	0
November 2010	B3A09	0	0	0	0	0	2	16	0	0
November 2010	B3A11	0	0	0	0	0	0	10	0	0
November 2010	B3A12	0	0	4	0	0	1	1	0	0
November 2010	B3A13	0	0	0	0	0	0	11	0	0
November 2010	B3A14	0	0	1	0	0	3	10	0	0
November 2010	B3A16	0	0	6	0	0	0	3	0	0
November 2010	B3A17	0	0	0	0	0	0	5	0	0
November 2010	B3A19	0	0	1	0	0	0	22	0	0
November 2010	B3A20	0	0	0	0	0	0	0	0	0
November 2010	B3A21	0	0	0	0	0	0	0	0	0
November 2010	B3A22	0	0	1	0	0	1	2	0	0
November 2010	B3A23	0	0	7	0	0	4	13	0	0
November 2010	B3A24	0	0	0	0	0	4	1	0	0
November 2010	B4A01	0	0	0	0	0	6	7	0	0
November 2010	B4A02	0	0	0	0	0	0	0	0	0
November 2010	B4A04	0	0	0	0	0	5	8	0	0
November 2010	B4A06	0	0	0	0	0	0	1	0	0
November 2010	B4A07	0	0	0	0	0	0	0	0	0
November 2010	B4A08	0	0	0	0	0	4	1	0	0

Date	Plot	*Cryptopygus thermophilus*	*Desoria violacea*	*Isotomiella minor*	*Isotomodes productus*	*Folsomia quadrioculata*	*Isotumurus palustris*	*Parisotoma notabilis*	*Isotomurus fucicolus*	*Proisotoma minuta*
November 2010	B4A09	0	0	0	0	0	0	2	0	0
November 2010	B4A10	0	0	0	0	0	0	4	0	0
November 2010	B4A11	0	0	0	0	0	0	10	0	0
November 2010	B4A12	0	0	0	0	0	0	0	0	0
November 2010	B4A13	0	0	0	0	0	0	0	0	0
November 2010	B4A14	0	0	0	0	0	0	0	0	0
November 2010	B4A15	0	0	0	0	0	0	2	0	0
November 2010	B4A16	0	0	0	0	0	0	0	0	0
November 2010	B4A17	0	0	0	0	0	0	1	0	0
November 2010	B4A18	0	0	0	0	0	0	2	0	0
November 2010	B4A20	0	0	0	0	0	0	0	0	0
November 2010	B4A21	0	0	0	0	0	0	0	0	0
November 2010	B4A22	0	0	0	0	0	0	0	0	0
July 2013	B1A01	0	0	0	0	0	0	0	0	0
July 2013	B1A02	0	0	0	0	0	0	0	0	0
July 2013	B1A03	0	0	0	0	0	0	0	0	0
July 2013	B1A04	0	0	0	0	0	0	0	0	0
July 2013	B1A05	0	0	0	0	0	0	0	0	0
July 2013	B1A06	0	0	0	0	0	0	0	0	0
July 2013	B1A07	0	0	0	0	0	0	0	0	0
July 2013	B1A08	0	0	0	0	0	0	0	0	0
July 2013	B1A11	0	0	0	0	0	0	0	0	0
July 2013	B1A12	0	0	0	0	0	0	0	0	0
July 2013	B1A13	0	0	0	0	0	0	0	0	0
July 2013	B1A14	0	0	0	0	0	0	0	0	0
July 2013	B1A15	0	0	**0**	0	0	0	0	0	0
July 2013	B1A16	0	0	0	0	0	0	0	0	0
July 2013	B1A17	0	0	0	0	0	0	0	0	0
July 2013	B1A18	0	0	0	0	0	0	0	0	0
July 2013	B1A19	0	0	0	0	0	0	0	0	0
July 2013	B1A20	0	0	**0**	0	0	0	0	0	0
July 2013	B1A21	0	0	0	0	0	0	0	0	0
July 2013	B1A22	0	0	**0**	0	0	0	0	0	0
July 2013	B2A01	0	0	**0**	0	1	0	0	0	0
July 2013	B2A02	0	0	0	0	0	0	0	0	0
July 2013	B2A03	0	0	0	0	0	0	0	0	0

Date	Plot	*Cryptopygus thermophilus*	*Desoria violacea*	*Isotomiella minor*	*Isotomodes productus*	*Folsomia quadrioculata*	*Isotumurus palustris*	*Parisotoma notabilis*	*Isotomurus fucicolus*	*Proisotoma minuta*
July 2013	B2A04	0	0	0	0	0	0	0	0	0
July 2013	B2A05	0	0	0	0	0	0	0	0	0
July 2013	B2A06	0	0	0	0	0	0	0	0	0
July 2013	B2A08	0	0	0	0	0	0	0	0	0
July 2013	B2A09	0	0	0	0	0	0	0	0	0
July 2013	B2A10	0	0	0	0	0	0	0	0	0
July 2013	B2A12	0	0	1	0	0	0	0	0	0
July 2013	B2A13	0	0	**0**	0	0	0	0	0	0
July 2013	B2A14	0	0	**0**	0	0	0	1	0	0
July 2013	B2A15	0	0	0	0	0	0	0	0	0
July 2013	B2A16	0	0	0	0	0	0	0	0	0
July 2013	B2A17	0	0	0	0	0	0	0	0	0
July 2013	B2A18	0	0	0	0	0	0	0	0	0
July 2013	B2A19	0	0	0	0	0	0	0	0	0
July 2013	B2A20	0	0	0	0	0	0	0	0	0
July 2013	B2A21	0	0	0	0	0	0	0	0	0
July 2013	B2A22	0	0	0	0	0	0	0	0	0
July 2013	B3A01	0	0	0	0	0	0	0	0	0
July 2013	B3A02	0	0	0	0	0	0	0	0	0
July 2013	B3A03	0	0	0	0	0	0	0	0	0
July 2013	B3A04	0	0	0	0	0	0	0	0	0
July 2013	B3A05	0	0	0	0	0	0	0	0	0
July 2013	B3A06	0	0	0	0	0	0	0	0	0
July 2013	B3A07	0	0	0	0	0	0	0	0	0
July 2013	B3A08	0	0	0	0	0	0	0	0	0
July 2013	B3A09	0	0	**0**	0	0	0	0	0	0
July 2013	B3A11	0	0	0	0	0	0	0	0	0
July 2013	B3A12	0	0	0	0	0	0	0	0	0
July 2013	B3A13	0	0	**0**	0	0	0	0	0	0
July 2013	B3A14	0	0	0	0	0	0	0	0	0
July 2013	B3A16	0	0	0	0	0	0	1	0	0
July 2013	B3A17	0	0	0	0	0	0	0	0	0
July 2013	B3A19	0	0	0	0	0	0	0	0	0
July 2013	B3A20	0	0	0	0	0	0	0	0	0
July 2013	B3A21	0	0	0	0	0	0	0	0	0
July 2013	B3A22	0	0	0	0	0	0	0	0	0

Date	Plot	*Cryptopygus thermophilus*	*Desoria violacea*	*Isotomiella minor*	*Isotomodes productus*	*Folsomia quadrioculata*	*Isotumurus palustris*	*Parisotoma notabilis*	*Isotomurus fucicolus*	*Proisotoma minuta*
July 2013	B3A23	0	0	0	0	0	0	0	1	0
July 2013	B3A24	0	0	0	0	0	0	0	0	0
July 2013	B4A01	0	0	0	0	0	0	0	0	0
July 2013	B4A02	0	0	0	0	1	0	0	0	0
July 2013	B4A04	0	0	0	0	0	0	0	0	1
July 2013	B4A06	0	0	0	0	0	0	1	0	0
July 2013	B4A07	0	0	0	0	0	0	0	0	0
July 2013	B4A08	0	0	0	0	0	0	0	0	0
July 2013	B4A09	0	0	**0**	0	0	0	0	0	0
July 2013	B4A10	0	0	**0**	0	0	0	0	0	0
July 2013	B4A11	0	0	0	0	0	0	0	0	0
July 2013	B4A12	0	0	0	0	0	0	0	0	0
July 2013	B4A13	0	0	0	0	0	0	0	0	0
July 2013	B4A14	0	0	0	0	0	0	0	0	0
July 2013	B4A15	0	0	0	0	0	0	0	0	0
July 2013	B4A16	0	0	0	0	0	0	0	0	0
July 2013	B4A17	0	0	**0**	0	0	0	0	0	0
July 2013	B4A18	0	0	0	0	0	0	0	0	0
July 2013	B4A20	0	0	0	0	0	0	0	0	0
July 2013	B4A21	0	0	**0**	0	0	0	0	0	0
July 2013	B4A22	0	0	0	0	0	0	0	0	0
October 2013	B1A01	16	0	0	0	0	0	5	0	0
October 2013	B1A02	0	0	0	0	0	0	0	0	0
October 2013	B1A03	19	0	16	0	0	0	0	0	0
October 2013	B1A04	0	0	12	0	0	0	0	0	0
October 2013	B1A05	0	0	0	0	0	0	0	0	0
October 2013	B1A06	16	0	15	18	0	0	0	0	0
October 2013	B1A07	23	0	3	0	0	0	0	0	0
October 2013	B1A08	0	1	0	0	0	0	0	0	0
October 2013	B1A11	1	0	0	0	0	0	0	0	0
October 2013	B1A12	5	0	0	0	0	0	0	0	0
October 2013	B1A13	0	0	0	0	0	0	0	0	0
October 2013	B1A14	0	0	0	0	0	0	0	0	0
October 2013	B1A15	0	0	1	0	1	0	0	0	0
October 2013	B1A16	0	0	0	0	0	0	0	0	0
October 2013	B1A17	13	0	3	0	0	0	0	0	0

Date	Plot	*Cryptopygus thermophilus*	*Desoria violacea*	*Isotomiella minor*	*Isotomodes productus*	*Folsomia quadrioculata*	*Isotumurus palustris*	*Parisotoma notabilis*	*Isotomurus fucicolus*	*Proisotoma minuta*
October 2013	B1A18	0	0	0	0	0	0	0	0	0
October 2013	B1A19	0	0	0	0	0	0	0	0	0
October 2013	B1A20	1	0	0	0	0	0	0	0	0
October 2013	B1A21	14	0	0	0	0	0	0	0	0
October 2013	B1A22	10	1	0	0	0	0	0	0	0
October 2013	B2A01	0	0	0	0	0	0	0	0	0
October 2013	B2A02	19	0	0	0	0	0	0	0	0
October 2013	B2A03	4	0	0	0	0	0	0	0	0
October 2013	B2A04	0	0	0	0	0	0	0	0	0
October 2013	B2A05	0	0	0	0	0	0	0	0	0
October 2013	B2A06	0	0	0	0	0	0	0	0	0
October 2013	B2A08	2	0	0	0	0	0	0	0	0
October 2013	B2A09	0	0	0	0	0	0	0	0	0
October 2013	B2A10	14	1	0	0	0	0	0	0	0
October 2013	B2A12	0	0	0	0	0	0	0	0	0
October 2013	B2A13	1	0	0	0	0	0	0	0	0
October 2013	B2A14	10	0	1	0	0	0	0	0	0
October 2013	B2A15	3	0	0	0	0	0	0	0	0
October 2013	B2A16	2	0	0	0	0	0	0	0	0
October 2013	B2A17	7	0	0	0	0	0	0	0	0
October 2013	B2A18	12	0	1	0	0	0	0	0	0
October 2013	B2A19	3	0	0	0	0	0	0	0	0
October 2013	B2A20	0	0	0	0	0	0	0	0	0
October 2013	B2A21	9	0	0	0	0	0	0	0	0
October 2013	B2A22	0	0	0	0	0	0	0	0	0
October 2013	B3A01	12	0	0	0	0	0	0	0	0
October 2013	B3A02	4	0	0	0	0	0	0	0	0
October 2013	B3A03	0	0	0	0	0	0	0	0	0
October 2013	B3A04	0	0	0	0	0	0	0	0	0
October 2013	B3A05	10	0	5	0	0	0	0	0	0
October 2013	B3A06	6	0	0	0	0	0	0	0	0
October 2013	B3A07	8	0	0	0	0	0	0	0	0
October 2013	B3A08	0	0	0	0	0	0	0	0	0
October 2013	B3A09	2	0	0	0	0	0	0	0	0
October 2013	B3A11	7	0	0	0	0	0	0	0	0
October 2013	B3A12	0	0	0	0	0	0	0	0	0

Date	Plot	*Cryptopygus thermophilus*	*Desoria violacea*	*Isotomiella minor*	*Isotomodes productus*	*Folsomia quadrioculata*	*Isotumurus palustris*	*Parisotoma notabilis*	*Isotomurus fucicolus*	*Proisotoma minuta*
October 2013	B3A13	1	0	0	0	0	0	0	0	0
October 2013	B3A14	0	0	0	0	0	0	0	0	0
October 2013	B3A16	0	0	0	0	0	0	0	0	0
October 2013	B3A17	6	0	0	0	0	0	0	0	0
October 2013	B3A19	7	0	0	0	0	0	0	0	0
October 2013	B3A20	0	0	1	0	0	0	0	0	0
October 2013	B3A21	1	0	5	0	0	0	0	0	0
October 2013	B3A22	0	0	5	0	0	0	0	0	0
October 2013	B3A23	3	0	2	0	0	0	0	0	0
October 2013	B3A24	12	0	0	0	0	0	0	0	0
October 2013	B4A01	0	0	3	0	0	0	2	0	0
October 2013	B4A02	0	0	0	0	0	0	0	0	0
October 2013	B4A04	0	0	0	0	0	0	0	0	0
October 2013	B4A06	2	0	0	0	0	0	0	0	0
October 2013	B4A07	0	0	4	0	0	0	0	0	0
October 2013	B4A08	0	0	0	0	0	0	0	0	0
October 2013	B4A09	10	0	0	0	0	0	2	0	0
October 2013	B4A10	26	0	0	0	0	0	0	0	0
October 2013	B4A11	8	0	0	0	0	0	0	0	0
October 2013	B4A12	2	0	0	0	0	0	0	0	0
October 2013	B4A13	0	0	0	0	0	0	0	0	0
October 2013	B4A14	0	0	0	0	0	0	1	0	0
October 2013	B4A15	4	0	0	0	0	0	0	0	0
October 2013	B4A16	1	0	0	0	0	0	0	0	0
October 2013	B4A17	1	0	0	0	0	0	0	0	0
October 2013	B4A18	0	0	0	0	0	0	0	0	0
October 2013	B4A20	14	0	0	0	0	0	0	0	0
October 2013	B4A21	3	0	0	0	0	0	0	0	0
October 2013	B4A22	10	0	0	0	0	0	0	0	0

Dataset

Collembola species
Type raw
Unit

Date	Plot	*Megalothorax minimus*	*Mesaphorura macrochaeta*	*Metaphorura affinis*	*Stenaphorurella denisi*	*Paratullbergia macdougalli*	*Onychiurus jubilarius*	*Supraphorura furcifera*	*Protaphorura armata*
November 2010	B1A01	0	0	0	0	0	0	0	0
November 2010	B1A02	0	11	0	0	0	1	0	0
November 2010	B1A03	6	5	0	0	0	0	0	0
November 2010	B1A04	0	11	0	0	0	4	0	0
November 2010	B1A05	0	3	0	0	0	6	0	0
November 2010	B1A06	0	20	0	2	0	0	0	0
November 2010	B1A07	0	18	0	1	0	0	0	0
November 2010	B1A08	0	4	0	0	0	0	0	0
November 2010	B1A11	0	1	0	0	0	0	0	0
November 2010	B1A12	0	0	0	0	0	0	0	0
November 2010	B1A13	0	3	0	0	0	1	0	0
November 2010	B1A14	0	0	0	0	0	0	0	0
November 2010	B1A15	0	0	0	0	0	0	0	0
November 2010	B1A16	0	2	0	0	0	0	0	0
November 2010	B1A17	0	33	0	0	0	0	0	0
November 2010	B1A18	0	0	0	0	0	0	0	0
November 2010	B1A19	0	0	0	16	0	1	0	0
November 2010	B1A20	1	18	0	8	0	0	0	0
November 2010	B1A21	5	1	0	13	0	8	0	0
November 2010	B1A22	0	0	0	6	0	0	0	0
November 2010	B2A01	3	4	0	6	0	0	0	0
November 2010	B2A02	0	0	0	0	0	0	0	0
November 2010	B2A03	1	3	0	0	0	0	0	0
November 2010	B2A04	0	3	0	5	0	0	0	0
November 2010	B2A05	0	9	0	6	0	6	0	0
November 2010	B2A06	0	7	0	2	0	7	0	0
November 2010	B2A08	1	20	0	9	0	35	0	0
November 2010	B2A09	0	0	0	32	0	8	0	0
November 2010	B2A10	1	68	0	0	0	9	0	0
November 2010	B2A12	0	10	0	7	0	0	0	0
November 2010	B2A13	0	3	0	1	0	0	0	0

Date	Plot	*Megalothorax minimus*	*Mesaphorura macrochaeta*	*Metaphorura affinis*	*Stenaphorurella denisi*	*Paratullbergia macdougalli*	*Onychiurus jubilarius*	*Supraphorura furcifera*	*Protaphorura armata*
November 2010	B2A14	0	5	0	0	0	0	0	0
November 2010	B2A15	0	14	0	0	0	0	0	0
November 2010	B2A16	3	34	0	0	0	0	0	0
November 2010	B2A17	0	3	0	0	0	0	0	0
November 2010	B2A18	2	7	0	0	0	0	0	0
November 2010	B2A19	0	0	0	2	0	0	0	0
November 2010	B2A20	0	0	0	5	0	2	0	0
November 2010	B2A21	6	15	47	18	0	4	0	0
November 2010	B2A22	0	0	0	0	0	0	0	0
November 2010	B3A01	0	0	0	5	0	2	0	0
November 2010	B3A02	0	15	0	9	0	5	3	0
November 2010	B3A03	0	1	0	0	0	0	0	0
November 2010	B3A04	4	0	0	14	0	0	0	0
November 2010	B3A05	4	5	0	0	0	1	0	0
November 2010	B3A06	1	11	0	3	0	0	0	0
November 2010	B3A07	1	0	0	1	0	0	0	0
November 2010	B3A08	1	5	0	4	0	0	0	0
November 2010	B3A09	0	0	0	1	0	0	0	0
November 2010	B3A11	9	0	0	0	0	1	0	0
November 2010	B3A12	0	0	0	10	0	1	0	0
November 2010	B3A13	1	1	0	9	0	0	0	0
November 2010	B3A14	1	2	0	0	0	0	0	0
November 2010	B3A16	4	4	0	8	0	0	0	0
November 2010	B3A17	0	1	0	0	0	4	0	0
November 2010	B3A19	2	38	0	0	0	0	0	0
November 2010	B3A20	1	4	0	5	0	0	0	0
November 2010	B3A21	0	0	0	0	0	0	0	0
November 2010	B3A22	9	0	0	3	0	0	0	0
November 2010	B3A23	1	3	0	0	0	1	0	0
November 2010	B3A24	1	0	0	2	0	0	0	0
November 2010	B4A01	15	6	0	7	0	1	0	0
November 2010	B4A02	0	0	0	4	0	0	0	0
November 2010	B4A04	3	5	0	0	0	0	0	0
November 2010	B4A06	0	4	0	2	0	0	0	0
November 2010	B4A07	0	3	0	1	0	6	0	0
November 2010	B4A08	1	11	0	2	0	3	0	0

Date	Plot	*Megalothorax minimus*	*Mesaphorura macrochaeta*	*Metaphorura affinis*	*Stenaphorurella denisi*	*Paratullbergia macdougalli*	*Onychiurus jubilarius*	*Supraphorura furcifera*	*Protaphorura armata*
November 2010	B4A09	1	4	0	14	0	22	0	0
November 2010	B4A10	1	3	0	1	0	2	0	0
November 2010	B4A11	0	2	0	0	0	5	0	0
November 2010	B4A12	0	5	0	0	0	0	0	0
November 2010	B4A13	0	6	0	0	0	0	0	0
November 2010	B4A14	0	4	0	16	0	0	0	0
November 2010	B4A15	0	2	0	8	0	14	0	0
November 2010	B4A16	0	2	0	3	0	0	0	0
November 2010	B4A17	0	6	0	1	0	0	0	0
November 2010	B4A18	2	1	0	6	0	0	0	0
November 2010	B4A20	0	3	0	3	0	0	0	0
November 2010	B4A21	0	4	0	6	0	2	0	0
November 2010	B4A22	0	2	0	1	0	0	0	0
July 2013	B1A01	0	0	0	0	0	0	0	1
July 2013	B1A02	0	0	0	0	0	0	0	0
July 2013	B1A03	0	0	0	0	0	0	0	0
July 2013	B1A04	0	0	0	0	0	0	0	0
July 2013	B1A05	0	0	0	0	0	0	0	0
July 2013	B1A06	0	0	0	0	0	0	0	0
July 2013	B1A07	0	0	0	0	0	0	0	0
July 2013	B1A08	0	0	0	0	0	0	0	0
July 2013	B1A11	0	0	0	0	0	0	0	0
July 2013	B1A12	0	0	0	0	0	0	0	0
July 2013	B1A13	0	0	0	0	0	0	0	0
July 2013	B1A14	0	0	0	0	0	0	0	1
July 2013	B1A15	0	0	0	0	0	0	0	0
July 2013	B1A16	0	0	0	0	0	0	0	0
July 2013	B1A17	0	0	0	0	0	0	0	0
July 2013	B1A18	0	0	0	0	0	0	0	0
July 2013	B1A19	0	0	0	0	0	0	0	0
July 2013	B1A20	0	0	0	0	1	0	0	0
July 2013	B1A21	0	0	0	0	0	0	0	0
July 2013	B1A22	0	0	0	0	1	0	0	0
July 2013	B2A01	0	0	0	0	0	0	0	0
July 2013	B2A02	0	0	0	0	0	0	0	0
July 2013	B2A03	0	0	0	0	0	0	0	0

Date	Plot	*Megalothorax minimus*	*Mesaphorura macrochaeta*	*Metaphorura affinis*	*Stenaphorurella denisi*	*Paratullbergia macdougalli*	*Onychiurus jubilarius*	*Supraphorura furcifera*	*Protaphorura armata*
July 2013	B2A04	0	0	0	0	0	0	0	0
July 2013	B2A05	0	0	0	0	0	0	0	0
July 2013	B2A06	0	0	0	0	0	0	0	0
July 2013	B2A08	0	0	0	0	0	0	0	0
July 2013	B2A09	0	0	0	0	0	0	0	0
July 2013	B2A10	0	0	0	0	0	0	0	0
July 2013	B2A12	0	0	0	0	0	0	0	0
July 2013	B2A13	0	0	0	0	0	0	0	1
July 2013	B2A14	0	0	0	0	1	0	0	0
July 2013	B2A15	0	0	0	0	0	0	0	0
July 2013	B2A16	0	0	0	0	0	0	0	0
July 2013	B2A17	0	0	0	0	0	0	0	0
July 2013	B2A18	0	0	0	0	0	0	0	0
July 2013	B2A19	0	0	0	0	0	0	0	0
July 2013	B2A20	0	0	0	0	0	0	0	0
July 2013	B2A21	0	0	0	0	0	0	0	0
July 2013	B2A22	0	0	0	0	0	0	0	0
July 2013	B3A01	0	0	0	0	0	0	0	0
July 2013	B3A02	0	0	0	0	0	0	0	0
July 2013	B3A03	0	0	0	0	0	0	0	0
July 2013	B3A04	0	0	0	0	0	0	0	0
July 2013	B3A05	0	0	0	0	0	0	0	0
July 2013	B3A06	0	0	0	0	0	0	0	0
July 2013	B3A07	0	0	0	0	0	0	0	0
July 2013	B3A08	0	0	0	0	0	0	0	0
July 2013	B3A09	0	0	0	0	0	0	0	0
July 2013	B3A11	0	0	0	0	0	0	0	0
July 2013	B3A12	0	0	0	0	0	0	0	0
July 2013	B3A13	0	0	0	0	0	0	0	0
July 2013	B3A14	0	0	0	0	0	0	0	0
July 2013	B3A16	0	0	0	0	0	0	0	0
July 2013	B3A17	0	0	0	0	0	0	0	0
July 2013	B3A19	0	0	0	0	0	0	0	0
July 2013	B3A20	0	0	0	0	0	0	0	0
July 2013	B3A21	0	0	0	0	0	0	0	0
July 2013	B3A22	0	0	0	0	0	0	0	0

Date	Plot	*Megalothorax minimus*	*Mesaphorura macrochaeta*	*Metaphorura affinis*	*Stenaphorurella denisi*	*Paratullbergia macdougalli*	*Onychiurus jubilarius*	*Supraphorura furcifera*	*Protaphorura armata*
July 2013	B3A23	0	0	0	0	0	0	0	0
July 2013	B3A24	0	0	0	0	0	0	0	0
July 2013	B4A01	0	0	0	0	0	0	0	0
July 2013	B4A02	0	0	0	0	0	0	0	0
July 2013	B4A04	0	0	0	0	0	0	0	0
July 2013	B4A06	0	0	0	0	0	0	0	0
July 2013	B4A07	0	0	0	0	0	0	0	0
July 2013	B4A08	0	0	0	0	0	0	0	0
July 2013	B4A09	0	0	0	0	0	0	0	0
July 2013	B4A10	0	0	0	0	0	0	0	0
July 2013	B4A11	0	0	0	0	0	0	0	0
July 2013	B4A12	0	0	0	0	0	0	0	0
July 2013	B4A13	0	0	0	0	0	0	0	0
July 2013	B4A14	0	0	0	0	0	0	0	1
July 2013	B4A15	0	0	0	0	0	0	0	0
July 2013	B4A16	0	0	0	0	0	0	0	0
July 2013	B4A17	0	0	0	0	0	0	0	0
July 2013	B4A18	0	0	0	0	0	0	0	0
July 2013	B4A20	0	0	0	0	0	0	0	0
July 2013	B4A21	0	0	0	0	0	0	0	0
July 2013	B4A22	0	0	0	0	0	0	0	0
October 2013	B1A01	0	0	0	0	0	0	0	0
October 2013	B1A02	0	0	0	0	0	0	0	0
October 2013	B1A03	0	0	0	0	0	0	0	0
October 2013	B1A04	0	0	0	0	0	0	0	0
October 2013	B1A05	0	0	0	0	0	0	0	0
October 2013	B1A06	0	4	0	4	0	0	0	0
October 2013	B1A07	0	0	0	0	0	0	0	0
October 2013	B1A08	0	0	0	1	0	0	0	0
October 2013	B1A11	0	0	0	9	0	0	0	0
October 2013	B1A12	0	0	0	1	0	0	0	0
October 2013	B1A13	0	0	0	0	0	0	0	0
October 2013	B1A14	0	0	0	1	0	0	0	0
October 2013	B1A15	2	1	0	0	0	0	0	0
October 2013	B1A16	0	1	0	8	0	0	0	0
October 2013	B1A17	0	0	0	0	0	0	0	0

Date	Plot	*Megalothorax minimus*	*Mesaphorura macrochaeta*	*Metaphorura affinis*	*Stenaphorurella denisi*	*Paratullbergia macdougalli*	*Onychiurus jubilarius*	*Supraphorura furcifera*	*Protaphorura armata*
October 2013	B1A18	0	1	0	0	0	0	0	0
October 2013	B1A19	0	0	0	5	0	0	0	0
October 2013	B1A20	0	0	0	57	0	0	0	0
October 2013	B1A21	0	0	0	0	0	0	0	0
October 2013	B1A22	0	0	0	48	0	0	0	0
October 2013	B2A01	0	0	0	13	0	0	0	0
October 2013	B2A02	0	0	0	7	0	0	0	0
October 2013	B2A03	0	0	0	20	0	0	0	0
October 2013	B2A04	0	0	0	0	0	0	0	0
October 2013	B2A05	0	0	0	0	0	0	0	0
October 2013	B2A06	0	0	0	0	0	0	0	0
October 2013	B2A08	0	0	0	0	0	0	0	0
October 2013	B2A09	0	0	0	59	0	0	0	0
October 2013	B2A10	0	0	0	16	0	0	0	0
October 2013	B2A12	0	0	0	11	0	0	0	0
October 2013	B2A13	0	0	0	0	0	0	0	0
October 2013	B2A14	0	0	0	13	0	0	0	0
October 2013	B2A15	0	0	0	0	0	0	0	0
October 2013	B2A16	0	0	0	10	0	0	0	0
October 2013	B2A17	0	0	0	55	0	0	0	0
October 2013	B2A18	0	0	0	55	0	0	0	0
October 2013	B2A19	0	0	0	62	0	0	0	0
October 2013	B2A20	0	0	0	1	0	0	0	0
October 2013	B2A21	0	0	0	8	0	0	0	0
October 2013	B2A22	0	0	0	14	0	0	0	0
October 2013	B3A01	0	0	0	10	0	0	0	0
October 2013	B3A02	0	0	0	0	0	0	0	0
October 2013	B3A03	0	0	0	0	0	0	0	0
October 2013	B3A04	0	0	0	6	0	0	0	0
October 2013	B3A05	0	0	0	3	0	0	0	0
October 2013	B3A06	0	0	0	8	0	0	0	0
October 2013	B3A07	0	0	0	8	0	0	0	0
October 2013	B3A08	0	0	0	5	0	0	0	0
October 2013	B3A09	0	0	0	1	0	0	0	0
October 2013	B3A11	0	0	0	8	0	0	0	0
October 2013	B3A12	0	0	0	3	0	0	0	0

Date	Plot	*Megalothorax minimus*	*Mesaphorura macrochaeta*	*Metaphorura affinis*	*Stenaphorurella denisi*	*Paratullbergia macdougalli*	*Onychiurus jubilarius*	*Supraphorura furcifera*	*Protaphorura armata*
October 2013	B3A13	0	0	0	0	0	0	0	0
October 2013	B3A14	0	0	0	71	0	0	0	0
October 2013	B3A16	0	0	0	110	0	0	0	0
October 2013	B3A17	0	0	0	0	0	0	0	0
October 2013	B3A19	0	0	0	0	0	0	0	0
October 2013	B3A20	0	1	0	7	0	0	0	0
October 2013	B3A21	0	1	0	1	0	0	0	0
October 2013	B3A22	0	0	0	0	0	0	0	0
October 2013	B3A23	0	0	0	0	0	0	0	0
October 2013	B3A24	0	0	0	20	0	0	0	0
October 2013	B4A01	0	0	0	22	0	0	0	0
October 2013	B4A02	0	0	0	3	0	0	0	0
October 2013	B4A04	0	0	0	11	0	0	0	0
October 2013	B4A06	0	0	0	0	0	0	0	0
October 2013	B4A07	0	0	0	16	0	0	0	0
October 2013	B4A08	0	0	0	5	0	0	0	0
October 2013	B4A09	0	4	0	0	0	0	0	0
October 2013	B4A10	0	0	0	10	0	0	0	0
October 2013	B4A11	0	0	0	6	0	0	0	0
October 2013	B4A12	0	0	0	2	0	0	0	0
October 2013	B4A13	0	0	0	16	0	0	0	0
October 2013	B4A14	0	0	0	36	0	0	0	0
October 2013	B4A15	0	0	0	0	0	0	0	0
October 2013	B4A16	0	0	0	0	0	0	0	0
October 2013	B4A17	0	0	0	0	0	0	0	0
October 2013	B4A18	0	0	0	4	0	0	0	0
October 2013	B4A20	0	0	0	27	0	0	0	0
October 2013	B4A21	0	0	0	28	0	0	0	0
October 2013	B4A22	0	0	0	18	0	0	0	0

Dataset

Collembola species
Type raw
Unit

Date	Plot	*Sphaeridia pumilis*	*Sminthurinus aureus*	*Sminthurinus elegans*	*Sminthurinus niger*	*Sminthurus viridis*
November 2010	B1A01	0	0	0	0	0
November 2010	B1A02	0	0	0	0	0
November 2010	B1A03	1	0	0	0	0
November 2010	B1A04	0	0	0	0	0
November 2010	B1A05	0	0	0	0	0
November 2010	B1A06	0	0	0	1	0
November 2010	B1A07	0	0	0	0	0
November 2010	B1A08	0	0	0	4	0
November 2010	B1A11	0	0	0	1	0
November 2010	B1A12	0	0	0	0	0
November 2010	B1A13	0	0	0	5	0
November 2010	B1A14	0	1	0	0	0
November 2010	B1A15	0	0	0	0	0
November 2010	B1A16	0	0	0	0	0
November 2010	B1A17	0	0	0	0	0
November 2010	B1A18	0	0	0	0	0
November 2010	B1A19	0	0	0	3	0
November 2010	B1A20	0	0	0	0	0
November 2010	B1A21	0	1	0	0	0
November 2010	B1A22	0	0	0	4	0
November 2010	B2A01	0	0	0	2	0
November 2010	B2A02	0	0	0	0	0
November 2010	B2A03	0	0	0	0	0
November 2010	B2A04	0	0	0	0	0
November 2010	B2A05	0	0	0	1	0
November 2010	B2A06	0	0	0	2	0
November 2010	B2A08	0	0	0	0	0
November 2010	B2A09	0	0	0	0	0
November 2010	B2A10	0	0	0	0	0
November 2010	B2A12	0	0	0	0	0
November 2010	B2A13	0	0	0	0	0

Date	Plot	*Sphaeridia pumilis*	*Sminthurinus aureus*	*Sminthurinus elegans*	*Sminthurinus niger*	*Sminthurus viridis*
November 2010	B2A14	0	0	0	0	0
November 2010	B2A15	0	0	0	0	0
November 2010	B2A16	0	0	0	0	0
November 2010	B2A17	0	0	0	0	0
November 2010	B2A18	0	0	0	0	0
November 2010	B2A19	0	0	0	0	0
November 2010	B2A20	0	0	0	1	0
November 2010	B2A21	0	0	0	2	0
November 2010	B2A22	0	0	0	0	0
November 2010	B3A01	0	0	0	0	0
November 2010	B3A02	0	0	0	0	0
November 2010	B3A03	0	0	1	0	0
November 2010	B3A04	0	0	0	2	0
November 2010	B3A05	0	0	0	2	0
November 2010	B3A06	0	0	0	2	0
November 2010	B3A07	0	0	0	0	0
November 2010	B3A08	0	0	0	0	0
November 2010	B3A09	0	0	0	0	0
November 2010	B3A11	0	5	0	0	0
November 2010	B3A12	0	0	0	0	0
November 2010	B3A13	0	0	0	1	0
November 2010	B3A14	0	0	0	2	0
November 2010	B3A16	0	0	0	1	0
November 2010	B3A17	0	0	0	0	0
November 2010	B3A19	0	0	0	0	0
November 2010	B3A20	0	0	0	1	0
November 2010	B3A21	0	0	0	0	0
November 2010	B3A22	0	0	0	3	0
November 2010	B3A23	0	0	0	3	0
November 2010	B3A24	0	0	0	0	0
November 2010	B4A01	0	0	0	1	0
November 2010	B4A02	0	0	0	0	0
November 2010	B4A04	0	0	0	1	0
November 2010	B4A06	0	0	0	0	0
November 2010	B4A07	0	0	0	0	0
November 2010	B4A08	0	0	0	0	0

Date	Plot	*Sphaeridia pumilis*	*Sminthurinus aureus*	*Sminthurinus elegans*	*Sminthurinus niger*	*Sminthurus viridis*
November 2010	B4A09	0	0	0	0	0
November 2010	B4A10	0	0	0	0	0
November 2010	B4A11	0	0	0	0	0
November 2010	B4A12	0	0	0	0	0
November 2010	B4A13	0	0	0	0	0
November 2010	B4A14	0	0	0	0	0
November 2010	B4A15	0	0	0	0	0
November 2010	B4A16	0	0	0	0	0
November 2010	B4A17	0	0	0	0	0
November 2010	B4A18	0	0	0	0	0
November 2010	B4A20	0	0	0	0	0
November 2010	B4A21	0	0	0	0	0
November 2010	B4A22	0	0	0	0	0
July 2013	B1A01	0	0	0	0	0
July 2013	B1A02	0	0	0	0	0
July 2013	B1A03	0	0	0	0	0
July 2013	B1A04	0	0	0	0	0
July 2013	B1A05	0	0	0	0	0
July 2013	B1A06	0	0	0	0	0
July 2013	B1A07	0	0	0	0	0
July 2013	B1A08	0	0	0	0	0
July 2013	B1A11	0	0	0	**0**	0
July 2013	B1A12	0	0	0	**0**	0
July 2013	B1A13	0	0	0	0	0
July 2013	B1A14	1	0	0	0	0
July 2013	B1A15	0	0	0	0	0
July 2013	B1A16	0	0	0	0	0
July 2013	B1A17	0	0	0	0	0
July 2013	B1A18	0	0	0	0	0
July 2013	B1A19	0	0	0	0	0
July 2013	B1A20	0	0	0	**0**	0
July 2013	B1A21	0	0	0	0	0
July 2013	B1A22	0	0	0	**0**	0
July 2013	B2A01	0	0	0	**0**	0
July 2013	B2A02	0	0	0	0	0
July 2013	B2A03	0	0	0	0	0

Date	Plot	*Sphaeridia pumilis*	*Sminthurinus aureus*	*Sminthurinus elegans*	*Sminthurinus niger*	*Sminthurus viridis*
July 2013	B2A04	0	0	0	0	0
July 2013	B2A05	0	0	0	0	0
July 2013	B2A06	0	0	0	**0**	0
July 2013	B2A08	0	0	0	0	0
July 2013	B2A09	0	0	0	0	0
July 2013	B2A10	0	0	0	0	0
July 2013	B2A12	0	0	0	**0**	0
July 2013	B2A13	0	0	0	**0**	0
July 2013	B2A14	0	0	0	**0**	0
July 2013	B2A15	0	0	0	0	0
July 2013	B2A16	0	0	0	**0**	0
July 2013	B2A17	0	0	0	**1**	0
July 2013	B2A18	0	0	0	0	0
July 2013	B2A19	0	0	0	0	0
July 2013	B2A20	0	0	0	0	0
July 2013	B2A21	0	0	0	0	0
July 2013	B2A22	0	0	0	0	0
July 2013	B3A01	0	0	0	0	0
July 2013	B3A02	0	0	0	0	0
July 2013	B3A03	0	0	0	0	0
July 2013	B3A04	0	0	0	0	0
July 2013	B3A05	0	0	0	0	0
July 2013	B3A06	0	0	0	0	0
July 2013	B3A07	0	0	0	0	0
July 2013	B3A08	0	0	0	0	0
July 2013	B3A09	0	0	0	0	0
July 2013	B3A11	0	0	0	**0**	0
July 2013	B3A12	0	0	0	0	0
July 2013	B3A13	0	0	0	0	0
July 2013	B3A14	0	0	0	0	0
July 2013	B3A16	0	0	0	0	0
July 2013	B3A17	0	0	0	0	0
July 2013	B3A19	0	0	0	0	0
July 2013	B3A20	0	0	0	0	0
July 2013	B3A21	0	0	0	0	0
July 2013	B3A22	0	0	0	0	1

Date	Plot	*Sphaeridia pumilis*	*Sminthurinus aureus*	*Sminthurinus elegans*	*Sminthurinus niger*	*Sminthurus viridis*
July 2013	B3A23	0	0	0	0	0
July 2013	B3A24	0	0	0	0	0
July 2013	B4A01	0	0	0	0	0
July 2013	B4A02	0	0	0	0	0
July 2013	B4A04	0	0	0	0	0
July 2013	B4A06	0	0	0	0	0
July 2013	B4A07	0	0	0	0	0
July 2013	B4A08	0	0	0	0	0
July 2013	B4A09	0	0	0	0	0
July 2013	B4A10	0	0	0	0	0
July 2013	B4A11	0	0	0	0	0
July 2013	B4A12	0	0	0	0	0
July 2013	B4A13	0	0	0	0	0
July 2013	B4A14	1	0	0	0	0
July 2013	B4A15	0	0	0	0	0
July 2013	B4A16	0	0	0	0	0
July 2013	B4A17	0	0	0	0	0
July 2013	B4A18	0	0	0	0	0
July 2013	B4A20	0	0	0	0	0
July 2013	B4A21	0	0	0	0	0
July 2013	B4A22	0	0	0	0	0
October 2013	B1A01	0	0	0	0	0
October 2013	B1A02	0	0	0	0	0
October 2013	B1A03	0	0	0	0	0
October 2013	B1A04	0	0	0	0	0
October 2013	B1A05	0	0	0	0	0
October 2013	B1A06	0	12	0	0	0
October 2013	B1A07	0	0	0	0	0
October 2013	B1A08	0	0	0	0	0
October 2013	B1A11	0	0	0	0	0
October 2013	B1A12	0	0	0	0	0
October 2013	B1A13	0	0	0	0	0
October 2013	B1A14	0	0	0	0	0
October 2013	B1A15	0	0	0	0	0
October 2013	B1A16	0	0	0	0	0
October 2013	B1A17	0	0	0	0	0

Date	Plot	*Sphaeridia pumilis*	*Sminthurinus aureus*	*Sminthurinus elegans*	*Sminthurinus niger*	*Sminthurus viridis*
October 2013	B1A18	0	0	0	0	0
October 2013	B1A19	0	0	0	0	0
October 2013	B1A20	0	0	0	0	0
October 2013	B1A21	0	0	0	0	0
October 2013	B1A22	0	0	0	0	0
October 2013	B2A01	0	1	0	0	0
October 2013	B2A02	0	0	0	0	0
October 2013	B2A03	0	0	0	0	0
October 2013	B2A04	0	1	0	0	0
October 2013	B2A05	0	0	0	0	0
October 2013	B2A06	0	0	0	0	0
October 2013	B2A08	0	0	0	0	0
October 2013	B2A09	0	0	0	0	0
October 2013	B2A10	0	0	0	0	0
October 2013	B2A12	0	0	0	0	0
October 2013	B2A13	0	0	0	0	0
October 2013	B2A14	0	1	0	0	0
October 2013	B2A15	0	0	0	0	0
October 2013	B2A16	0	0	0	0	0
October 2013	B2A17	0	0	0	0	0
October 2013	B2A18	0	0	0	0	0
October 2013	B2A19	0	0	0	0	0
October 2013	B2A20	0	0	0	0	0
October 2013	B2A21	0	1	0	0	0
October 2013	B2A22	0	0	0	0	0
October 2013	B3A01	0	0	0	0	0
October 2013	B3A02	0	0	0	0	0
October 2013	B3A03	0	0	0	0	0
October 2013	B3A04	0	0	0	0	0
October 2013	B3A05	0	0	0	0	0
October 2013	B3A06	0	0	0	0	0
October 2013	B3A07	0	0	0	0	0
October 2013	B3A08	0	0	0	0	0
October 2013	B3A09	0	0	0	0	0
October 2013	B3A11	0	0	0	0	0
October 2013	B3A12	0	0	0	0	0

Date	Plot	*Sphaeridia pumilis*	*Sminthurinus aureus*	*Sminthurinus elegans*	*Sminthurinus niger*	*Sminthurus viridis*
October 2013	B3A13	0	0	0	0	0
October 2013	B3A14	0	0	0	0	0
October 2013	B3A16	0	0	0	0	0
October 2013	B3A17	0	0	0	0	0
October 2013	B3A19	0	0	0	0	0
October 2013	B3A20	0	0	0	0	0
October 2013	B3A21	0	0	0	0	0
October 2013	B3A22	0	0	0	0	0
October 2013	B3A23	0	1	0	0	0
October 2013	B3A24	0	0	0	0	0
October 2013	B4A01	0	0	0	0	0
October 2013	B4A02	0	0	0	0	0
October 2013	B4A04	0	0	0	0	0
October 2013	B4A06	0	0	0	0	0
October 2013	B4A07	0	0	0	0	0
October 2013	B4A08	0	0	0	0	0
October 2013	B4A09	0	1	0	0	0
October 2013	B4A10	0	0	0	0	0
October 2013	B4A11	0	0	0	0	0
October 2013	B4A12	0	0	0	0	0
October 2013	B4A13	0	0	0	0	0
October 2013	B4A14	0	0	0	0	0
October 2013	B4A15	0	0	0	0	0
October 2013	B4A16	0	0	0	0	0
October 2013	B4A17	0	0	0	0	0
October 2013	B4A18	0	0	0	0	0
October 2013	B4A20	0	0	0	0	0
October 2013	B4A21	0	0	0	0	0
October 2013	B4A22	0	0	0	0	0
