# Supplementary material for: Response of Collembola and Acari communities to summer flooding in a grassland plant diversity experiment
Source: PLoS One. 2018 Aug 30;13(8):e0202862. doi: 10.1371/journal.pone.0202862 (PMC6117009; doi:10.1371/journal.pone.0202862)
Supplement: S4 Table — (PDF) [file pone.0202862.s005.pdf]

Dataset

Oribatida species

Type raw

Unit individuals in soil cores of 5 cm diameter and 5 cm depth

Date	Plot	*Liebstadia similis*	*Oppiella nova*	*Punctoribates punctum*	*Rhysotritia ardua ardua*	*Scheloribates laevigatus*	*Tectocepheus velatus velatus*	*Tectocepheus velatus sarekensis*	*Oribatula excavata*	*Nothrus anauniensis*	*Nothrus pratensis*
November 2010	B1A01	0	8	0	0	0	0	5	1	0	0
November 2010	B1A02	0	0	0	0	0	0	1	0	0	0
November 2010	B1A03	21	31	0	0	0	0	1	0	0	0
November 2010	B1A04	0	5	0	0	0	1	1	245	0	0
November 2010	B1A05	0	3	0	1	0	0	0	0	0	0
November 2010	B1A06	0	11	0	2	0	0	0	0	0	0
November 2010	B1A07	0	0	0	2	0	0	0	0	0	0
November 2010	B1A08	0	0	0	0	0	0	0	0	0	0
November 2010	B1A11	0	31	0	2	0	0	8	0	1	3
November 2010	B1A12	0	0	0	2	0	0	0	0	0	0
November 2010	B1A14	0	0	1	0	8	0	4	0	0	0
November 2010	B1A16	0	4	0	5	0	0	1	0	0	0
November 2010	B1A17	0	0	0	0	0	0	0	0	0	0
November 2010	B1A19	0	4	0	0	0	0	1	0	0	0
November 2010	B1A20	0	0	0	1	0	0	1	0	1	0
November 2010	B1A21	0	1	0	0	0	0	0	0	0	0
November 2010	B1A22	0	0	0	3	0	0	3	0	0	0
November 2010	B2A01	0	0	0	0	0	0	1	0	0	0
November 2010	B2A03	0	1	0	0	0	0	0	0	0	0
November 2010	B2A04	0	0	0	0	0	0	0	0	0	0
November 2010	B2A05	0	7	0	0	0	0	0	0	0	0
November 2010	B2A06	0	3	14	0	0	0	0	0	0	0
November 2010	B2A08	0	8	0	4	0	0	1	0	0	0
November 2010	B2A09	0	1	1	10	0	0	1	0	0	0
November 2010	B2A10	0	1	3	0	0	0	1	0	0	0
November 2010	B2A12	0	0	0	0	1	0	0	0	0	0
November 2010	B2A13	0	0	0	0	0	0	0	0	0	0
November 2010	B2A14	0	11	0	3	0	0	2	0	0	0
November 2010	B2A15	0	0	0	0	0	0	0	0	0	0
November 2010	B2A16	0	0	0	0	0	0	0	0	0	0
November 2010	B2A17	0	2	0	0	0	0	0	0	0	0
November 2010	B2A18	0	0	0	0	0	0	3	0	0	0
November 2010	B2A19	0	3	0	0	0	0	1	0	0	0

Date	Plot	*Liebstadia similis*	*Oppiella nova*	*Punctoribates punctum*	*Rhysotritia ardua ardua*	*Scheloribates laevigatus*	*Tectocepheus velatus velatus*	*Tectocepheus velatus sarekensis*	*Oribatula excavata*	*Nothrus anauniensis*	*Nothrus pratensis*
November 2010	B2A20	0	2	0	0	0	0	1	0	0	0
November 2010	B2A21	0	0	0	0	0	0	0	0	0	3
November 2010	B2A22	0	0	0	1	0	0	0	0	0	0
November 2010	B3A01	0	0	0	0	0	0	0	0	0	0
November 2010	B3A02	0	0	0	0	0	0	2	0	0	0
November 2010	B3A03	0	0	0	0	0	0	0	0	0	0
November 2010	B3A04	0	0	0	0	0	0	2	0	0	0
November 2010	B3A05	0	0	0	0	0	0	0	0	0	0
November 2010	B3A06	0	0	0	0	0	0	6	0	0	0
November 2010	B3A07	0	0	0	1	0	0	0	0	0	0
November 2010	B3A08	0	3	0	0	0	0	0	0	0	0
November 2010	B3A09	0	4	0	0	0	0	3	0	0	0
November 2010	B3A11	0	10	0	0	0	0	1	0	0	0
November 2010	B3A12	0	0	0	0	0	0	0	0	0	0
November 2010	B3A13	0	0	0	0	0	0	1	0	0	0
November 2010	B3A14	0	0	0	0	0	0	0	0	0	0
November 2010	B3A16	0	0	0	0	0	0	0	0	0	0
November 2010	B3A17	0	0	0	0	0	0	0	0	0	0
November 2010	B3A19	0	0	0	0	0	0	1	0	0	0
November 2010	B3A20	0	0	0	0	0	0	1	0	0	0
November 2010	B3A21	0	0	0	0	0	0	0	0	0	0
November 2010	B3A22	0	0	1	0	0	0	0	0	0	0
November 2010	B3A23	0	0	0	2	0	0	1	0	0	0
November 2010	B3A24	0	0	0	0	0	0	0	0	0	0
November 2010	B4A01	0	0	0	0	0	0	0	0	0	0
November 2010	B4A02	0	0	0	0	0	0	0	0	0	0
November 2010	B4A04	0	1	0	0	0	0	5	0	0	0
November 2010	B4A06	0	0	0	0	0	0	0	0	0	0
November 2010	B4A07	0	0	0	0	0	0	0	0	0	0
November 2010	B4A08	0	0	0	0	0	0	0	0	0	0
November 2010	B4A09	0	0	0	0	0	0	0	0	0	0
November 2010	B4A10	0	10	0	0	0	1	3	0	0	0
November 2010	B4A11	0	0	0	0	0	0	0	0	0	0
November 2010	B4A12	0	0	0	0	0	0	0	0	0	0
November 2010	B4A13	0	0	0	0	0	1	0	0	0	0
November 2010	B4A14	0	0	0	0	0	0	0	0	0	0
November 2010	B4A15	0	0	0	0	0	0	0	0	0	0
November 2010	B4A17	0	0	0	0	0	0	0	0	0	0

Date	Plot	*Liebstadia similis*	*Oppiella nova*	*Punctoribates punctum*	*Rhysotritia ardua ardua*	*Scheloribates laevigatus*	*Tectocepheus velatus velatus*	*Tectocepheus velatus sarekensis*	*Oribatula excavata*	*Nothrus anauniensis*	*Nothrus pratensis*
November 2010	B4A18	0	0	0	0	0	0	0	0	0	0
November 2010	B4A20	0	0	0	0	0	0	0	0	0	0
November 2010	B4A21	0	0	0	0	0	1	0	0	0	0
November 2010	B4A22	0	0	0	0	0	0	0	0	0	0
July 2013	B1A01	0	0	0	0	0	0	3	0	0	0
July 2013	B1A02	0	0	0	0	0	0	0	0	0	0
July 2013	B1A03	0	0	0	0	0	0	0	0	0	0
July 2013	B1A04	0	0	0	0	0	0	0	0	0	0
July 2013	B1A05	0	0	0	0	0	0	0	0	0	0
July 2013	B1A06	0	0	0	0	0	0	0	0	0	0
July 2013	B1A07	0	0	0	0	0	0	0	0	0	0
July 2013	B1A08	0	0	0	0	0	0	0	0	0	0
July 2013	B1A11	0	0	0	0	2	0	0	0	0	0
July 2013	B1A12	0	0	0	0	0	0	1	0	0	0
July 2013	B1A13	0	0	0	0	0	0	0	0	0	0
July 2013	B1A14	0	0	0	0	0	0	0	0	0	0
July 2013	B1A15	0	0	0	0	0	0	0	0	0	0
July 2013	B1A16	0	1	0	0	0	0	0	0	0	0
July 2013	B1A17	0	0	0	0	0	0	0	0	0	0
July 2013	B1A18	0	0	0	0	0	0	0	0	0	0
July 2013	B1A19	0	0	0	0	0	0	0	0	0	0
July 2013	B1A20	0	0	0	1	0	0	3	0	0	0
July 2013	B1A21	0	0	0	0	0	0	0	0	0	0
July 2013	B1A22	0	0	0	0	0	0	0	0	0	0
July 2013	B4A01	0	0	0	0	0	0	0	0	0	0
July 2013	B4A02	0	0	0	0	0	0	1	0	0	0
July 2013	B4A04	0	0	0	0	0	0	0	0	0	0
July 2013	B4A06	0	0	0	0	0	0	0	0	0	0
July 2013	B4A07	0	0	0	0	0	0	0	0	0	0
July 2013	B4A08	0	0	0	0	5	0	0	0	0	0
July 2013	B4A09	0	0	0	0	0	0	8	0	0	0
July 2013	B4A10	0	0	0	0	0	0	1	0	0	0
July 2013	B4A11	0	0	0	0	0	0	0	0	0	0
July 2013	B4A12	0	0	0	0	0	0	0	0	0	0
July 2013	B4A13	0	0	0	0	0	0	0	0	0	0
July 2013	B4A14	0	0	0	0	0	0	0	0	0	0
July 2013	B4A15	0	0	0	0	0	0	0	0	0	0
July 2013	B4A16	0	0	0	0	0	0	0	0	0	0

Date	Plot	*Liebstadia similis*	*Oppiella nova*	*Punctoribates punctum*	*Rhysotritia ardua ardua*	*Scheloribates laevigatus*	*Tectocepheus velatus velatus*	*Tectocepheus velatus sarekensis*	*Oribatula excavata*	*Nothrus anauniensis*	*Nothrus pratensis*
July 2013	B4A17	0	0	0	0	0	0	0	0	0	0
July 2013	B4A18	0	0	0	0	0	0	3	0	0	0
July 2013	B4A20	0	0	0	0	0	0	0	0	0	0
July 2013	B4A21	0	0	0	0	0	0	0	0	0	0
July 2013	B4A22	0	0	0	0	0	0	0	0	0	0
July 2013	B2A01	0	0	0	0	0	0	0	0	0	0
July 2013	B2A02	0	0	0	0	0	0	0	0	0	0
July 2013	B2A03	0	0	0	0	0	0	0	0	0	0
July 2013	B2A04	0	0	0	0	0	0	0	0	0	0
July 2013	B2A05	0	0	0	0	0	0	0	0	0	0
July 2013	B2A06	0	0	0	0	0	0	0	0	0	0
July 2013	B2A08	0	0	0	0	0	0	3	0	0	0
July 2013	B2A09	0	0	2	0	0	0	2	0	0	0
July 2013	B2A10	0	0	0	0	0	0	0	0	0	0
July 2013	B2A12	0	0	0	0	1	0	0	0	0	0
July 2013	B2A13	0	0	0	0	0	0	0	0	0	0
July 2013	B2A14	0	0	0	0	0	0	0	0	0	0
July 2013	B2A15	0	0	0	0	0	0	0	0	0	0
July 2013	B2A16	0	0	0	0	0	0	0	0	0	0
July 2013	B2A17	0	0	0	0	0	0	0	0	0	0
July 2013	B2A18	0	0	0	0	0	0	0	0	0	0
July 2013	B2A19	0	0	0	0	0	0	0	0	0	0
July 2013	B2A20	0	0	0	0	0	0	0	0	0	0
July 2013	B2A21	0	0	1	0	0	0	0	0	0	0
July 2013	B2A22	0	0	0	0	0	0	3	0	0	0
July 2013	B3A01	0	0	0	0	0	0	0	0	0	0
July 2013	B3A02	0	0	0	0	0	0	0	0	0	0
July 2013	B3A03	0	0	0	0	0	0	0	0	0	0
July 2013	B3A04	0	0	0	0	0	0	0	0	0	0
July 2013	B3A05	0	0	0	0	0	0	0	0	0	0
July 2013	B3A06	0	0	0	0	0	0	0	0	0	0
July 2013	B3A07	0	0	0	0	0	0	0	0	0	0
July 2013	B3A08	0	0	0	0	0	0	0	0	0	0
July 2013	B3A09	0	0	0	0	0	0	0	0	0	0
July 2013	B3A11	0	0	1	0	3	0	1	0	0	0
July 2013	B3A12	0	0	0	0	0	0	0	0	0	0
July 2013	B3A13	0	0	0	0	0	0	0	0	0	0
July 2013	B3A14	0	0	0	0	0	0	0	0	0	0

Date	Plot	*Liebstadia similis*	*Oppiella nova*	*Punctoribates punctum*	*Rhysotritia ardua ardua*	*Scheloribates laevigatus*	*Tectocepheus velatus velatus*	*Tectocepheus velatus sarekensis*	*Oribatula excavata*	*Nothrus anauniensis*	*Nothrus pratensis*
July 2013	B3A16	0	0	0	0	0	0	0	0	0	0
July 2013	B3A17	0	0	0	0	0	0	0	0	0	0
July 2013	B3A19	0	0	0	0	0	0	0	0	0	0
July 2013	B3A20	0	0	0	0	0	0	0	0	0	0
July 2013	B3A21	0	0	0	0	1	0	1	0	0	0
July 2013	B3A22	0	0	0	0	0	0	0	0	0	0
July 2013	B3A23	0	0	0	0	0	0	0	0	0	0
July 2013	B3A24	0	0	0	0	0	0	0	0	0	0
October 2013	B1A01	0	11	0	4	0	0	0	1	0	0
October 2013	B1A02	0	0	0	0	7	0	5	0	0	0
October 2013	B1A03	0	4	0	3	2	0	1	0	0	0
October 2013	B1A04	0	3	0	2	0	0	2	0	0	0
October 2013	B1A05	0	0	0	0	0	0	0	0	0	0
October 2013	B1A06	0	53	0	0	0	0	1	0	0	0
October 2013	B1A07	0	19	0	1	0	0	0	0	0	0
October 2013	B1A08	0	0	0	0	0	0	0	0	0	0
October 2013	B1A11	0	29	0	3	0	0	1	0	0	0
October 2013	B1A12	0	1	0	0	0	0	0	0	0	0
October 2013	B1A13	0	0	0	0	0	0	0	0	0	0
October 2013	B1A14	0	68	8	0	22	0	13	0	0	0
October 2013	B1A15	0	0	0	0	0	0	0	0	0	0
October 2013	B1A16	0	16	0	0	0	0	0	0	0	0
October 2013	B1A17	0	3	0	0	0	0	0	0	0	0
October 2013	B1A18	0	0	0	0	0	0	0	0	0	0
October 2013	B1A19	0	0	0	0	0	0	0	0	0	0
October 2013	B1A20	0	0	0	0	0	0	1	0	0	0
October 2013	B1A21	0	0	0	0	0	0	1	0	0	0
October 2013	B1A22	0	0	0	0	0	0	1	0	0	0
October 2013	B4A01	0	0	0	0	0	0	0	0	0	0
October 2013	B4A02	0	5	0	0	0	0	0	0	0	0
October 2013	B4A04	0	0	0	0	0	0	0	1	0	0
October 2013	B4A06	0	0	0	0	0	0	0	0	0	0
October 2013	B4A07	0	7	0	0	0	0	0	0	0	0
October 2013	B4A08	0	0	0	0	21	0	0	8	0	0
October 2013	B4A09	0	0	0	0	0	0	2	0	0	0
October 2013	B4A10	0	5	0	0	0	0	1	0	0	0
October 2013	B4A11	0	0	0	0	0	0	0	0	0	0
October 2013	B4A12	0	5	0	0	1	0	0	0	0	0

Date	Plot	*Liebstadia similis*	*Oppiella nova*	*Punctoribates punctum*	*Rhysotritia ardua ardua*	*Scheloribates laevigatus*	*Tectocepheus velatus velatus*	*Tectocepheus velatus sarekensis*	*Oribatula excavata*	*Nothrus anauniensis*	*Nothrus pratensis*
October 2013	B4A13	0	0	0	0	0	0	0	0	0	0
October 2013	B4A14	0	2	0	0	0	0	1	0	0	0
October 2013	B4A15	0	14	0	0	0	0	1	0	0	0
October 2013	B4A16	0	0	0	0	0	0	0	0	0	0
October 2013	B4A17	0	1	0	0	0	0	0	0	0	0
October 2013	B4A18	0	0	0	0	0	0	1	0	0	0
October 2013	B4A20	0	0	0	0	0	0	0	0	0	0
October 2013	B4A21	0	1	0	0	0	0	4	0	0	0
October 2013	B4A22	0	4	0	0	0	0	1	0	0	0
October 2013	B2A01	0	4	0	0	0	0	1	0	0	0
October 2013	B2A02	0	5	0	0	2	0	10	0	0	0
October 2013	B2A03	0	3	0	0	0	0	2	0	0	0
October 2013	B2A04	0	1	0	0	0	0	0	0	0	0
October 2013	B2A05	0	2	0	0	0	0	0	0	0	0
October 2013	B2A06	0	5	2	0	0	0	0	0	0	0
October 2013	B2A08	0	5	1	0	0	0	0	0	0	0
October 2013	B2A09	0	3	5	0	0	0	0	0	0	0
October 2013	B2A10	0	0	0	0	0	0	0	0	0	0
October 2013	B2A12	0	2	0	0	1	0	0	0	0	0
October 2013	B2A13	0	0	0	0	0	0	0	0	0	0
October 2013	B2A14	0	2	0	0	0	0	0	0	0	0
October 2013	B2A15	0	0	0	0	0	0	0	0	0	0
October 2013	B2A16	0	0	0	0	0	0	0	0	0	0
October 2013	B2A17	0	0	0	0	0	0	0	0	0	0
October 2013	B2A18	0	0	0	0	0	0	0	0	0	0
October 2013	B2A19	0	1	0	2	0	0	0	0	0	0
October 2013	B2A20	0	0	0	0	0	0	0	0	0	0
October 2013	B2A21	0	7	5	0	0	0	1	0	0	0
October 2013	B2A22	0	0	5	0	0	0	2	0	0	0
October 2013	B3A01	0	0	0	0	5	0	0	0	0	0
October 2013	B3A02	0	0	0	0	0	0	0	0	0	0
October 2013	B3A03	0	0	0	0	0	0	0	0	0	0
October 2013	B3A04	0	0	0	0	0	0	0	0	0	0
October 2013	B3A05	0	0	0	0	0	0	1	0	0	0
October 2013	B3A06	0	1	0	0	0	0	0	0	0	0
October 2013	B3A07	0	0	0	0	0	0	2	0	0	0
October 2013	B3A08	0	1	0	0	0	0	0	0	0	0
October 2013	B3A09	0	0	0	0	0	0	1	0	0	0

Date	Plot	*Liebstadia similis*	*Oppiella nova*	*Punctoribates punctum*	*Rhysotritia ardua ardua*	*Scheloribates laevigatus*	*Tectocepheus velatus velatus*	*Tectocepheus velatus sarekensis*	*Oribatula excavata*	*Nothrus anauniensis*	*Nothrus pratensis*
October 2013	B3A11	0	22	2	0	13	0	2	0	0	0
October 2013	B3A12	0	0	0	0	0	0	1	0	0	0
October 2013	B3A13	0	0	0	0	0	0	0	0	0	0
October 2013	B3A14	0	0	0	0	0	0	0	0	0	0
October 2013	B3A16	0	0	0	0	0	0	0	0	0	0
October 2013	B3A17	0	0	0	0	0	0	0	0	0	0
October 2013	B3A19	0	12	0	0	0	0	0	0	0	0
October 2013	B3A20	0	0	0	0	0	0	0	0	0	0
October 2013	B3A21	0	1	0	0	0	0	0	0	0	0
October 2013	B3A22	0	0	0	0	0	0	0	0	0	0
October 2013	B3A23	0	1	0	0	0	0	0	0	0	0
October 2013	B3A24	0	0	0	0	0	0	1	0	0	0

Dataset

Oribatida species
Type raw
Unit

Date	Plot	*Ceratozetes psammophilus*	*Scheloribates initialis*	*Microppia minus*	*Zygoribatula frisiae*	*Protoribates capucinus*	*Tectocepheus minor*	*Hypochthonius rufulus*	*Steganacarus striculus*
November 2010	B1A01	0	0	0	0	0	0	0	0
November 2010	B1A02	0	0	0	0	0	0	0	0
November 2010	B1A03	0	0	0	0	0	0	0	0
November 2010	B1A04	0	0	0	0	0	0	0	0
November 2010	B1A05	0	0	0	0	0	0	0	0
November 2010	B1A06	0	0	0	0	0	0	0	0
November 2010	B1A07	0	0	0	0	0	0	0	0
November 2010	B1A08	0	0	0	0	0	0	0	0
November 2010	B1A11	0	0	0	0	0	0	0	0
November 2010	B1A12	0	0	0	0	0	0	0	0
November 2010	B1A14	0	0	0	0	0	0	0	0
November 2010	B1A16	0	0	0	0	0	0	0	0
November 2010	B1A17	0	0	0	0	0	0	0	0
November 2010	B1A19	0	0	0	0	0	0	0	0
November 2010	B1A20	1	0	0	0	0	0	0	0
November 2010	B1A21	0	0	0	0	0	0	0	0
November 2010	B1A22	0	0	0	0	0	0	0	0
November 2010	B2A01	0	0	0	0	0	0	0	0
November 2010	B2A03	0	0	0	0	0	0	0	0
November 2010	B2A04	0	0	0	0	0	0	0	0
November 2010	B2A05	0	0	0	0	0	0	0	0
November 2010	B2A06	1	0	0	0	0	0	0	0
November 2010	B2A08	0	0	0	0	0	0	0	0
November 2010	B2A09	0	0	0	0	0	0	0	0
November 2010	B2A10	1	0	0	0	0	0	0	0
November 2010	B2A12	0	0	0	0	0	0	0	0
November 2010	B2A13	0	0	0	0	0	0	0	0
November 2010	B2A14	0	0	0	0	0	0	0	0
November 2010	B2A15	0	0	0	0	0	0	0	0
November 2010	B2A16	0	0	0	0	0	0	0	0
November 2010	B2A17	0	0	0	0	0	0	0	0
November 2010	B2A18	0	0	0	0	0	0	0	0
November 2010	B2A19	0	0	0	0	0	0	0	0

Date	Plot	*Ceratozetes psammophilus*	*Scheloribates initialis*	*Microppia minus*	*Zygoribatula frisiae*	*Protoribates capucinus*	*Tectocepheus minor*	*Hypochthonius rufulus*	*Steganacarus striculus*
November 2010	B2A20	0	0	0	0	0	0	0	0
November 2010	B2A21	0	0	0	0	0	0	0	0
November 2010	B2A22	0	0	0	0	0	0	0	0
November 2010	B3A01	0	0	0	0	0	0	0	0
November 2010	B3A02	0	0	0	0	0	0	0	0
November 2010	B3A03	0	0	0	0	0	0	0	0
November 2010	B3A04	0	0	0	0	0	0	0	0
November 2010	B3A05	0	0	0	0	0	0	0	0
November 2010	B3A06	0	0	0	0	0	0	0	0
November 2010	B3A07	0	0	0	0	0	0	0	0
November 2010	B3A08	0	0	0	0	0	0	0	0
November 2010	B3A09	0	0	0	0	0	0	0	0
November 2010	B3A11	0	0	0	0	0	0	0	0
November 2010	B3A12	0	0	0	0	0	0	0	0
November 2010	B3A13	0	0	0	0	0	0	0	0
November 2010	B3A14	0	0	0	0	0	0	0	0
November 2010	B3A16	0	0	0	0	0	0	0	0
November 2010	B3A17	0	0	0	0	0	0	0	0
November 2010	B3A19	0	0	0	0	0	0	0	0
November 2010	B3A20	0	0	0	0	0	0	0	0
November 2010	B3A21	0	0	0	0	0	0	0	0
November 2010	B3A22	0	0	0	0	0	0	0	0
November 2010	B3A23	0	0	0	0	0	0	0	0
November 2010	B3A24	0	0	0	0	0	0	0	0
November 2010	B4A01	0	0	0	0	0	0	0	0
November 2010	B4A02	0	0	0	0	0	0	0	0
November 2010	B4A04	0	0	0	0	0	0	0	0
November 2010	B4A06	0	0	0	0	0	0	0	0
November 2010	B4A07	0	0	0	0	0	0	0	0
November 2010	B4A08	0	0	0	0	0	0	0	0
November 2010	B4A09	0	0	0	0	0	0	0	0
November 2010	B4A10	0	0	0	0	0	0	0	0
November 2010	B4A11	0	0	0	0	0	0	0	0
November 2010	B4A12	0	0	0	0	0	0	0	0
November 2010	B4A13	0	0	0	0	0	0	0	0
November 2010	B4A14	0	0	0	0	0	0	0	0
November 2010	B4A15	0	0	0	0	0	0	0	0
November 2010	B4A17	0	0	0	0	0	0	0	0

Date	Plot	*Ceratozetes psammophilus*	*Scheloribates initialis*	*Microppia minus*	*Zygoribatula frisiae*	*Protoribates capucinus*	*Tectocepheus minor*	*Hypochthonius rufulus*	*Steganacarus stricuIus*
November 2010	B4A18	0	0	0	0	0	0	0	0
November 2010	B4A20	0	0	0	0	0	0	0	0
November 2010	B4A21	0	0	0	0	0	0	0	0
November 2010	B4A22	0	0	0	0	0	0	0	0
July 2013	B1A01	0	0	0	0	0	0	0	0
July 2013	B1A02	0	0	0	0	0	0	0	0
July 2013	B1A03	0	0	0	0	0	0	0	0
July 2013	B1A04	0	0	0	0	0	0	0	0
July 2013	B1A05	0	0	0	0	0	0	0	0
July 2013	B1A06	0	0	0	0	0	0	0	0
July 2013	B1A07	0	0	0	0	0	0	0	0
July 2013	B1A08	0	0	0	0	0	0	0	0
July 2013	B1A11	0	0	0	0	0	0	0	0
July 2013	B1A12	0	0	0	0	0	0	0	0
July 2013	B1A13	0	0	0	0	0	0	0	0
July 2013	B1A14	0	0	0	0	0	0	0	0
July 2013	B1A15	0	0	0	0	0	0	0	0
July 2013	B1A16	0	0	0	0	0	0	0	0
July 2013	B1A17	0	0	0	0	0	0	0	0
July 2013	B1A18	0	0	0	0	0	0	0	0
July 2013	B1A19	0	0	0	0	0	0	0	0
July 2013	B1A20	0	0	0	0	0	0	0	0
July 2013	B1A21	0	0	0	0	0	0	0	0
July 2013	B1A22	0	0	0	0	0	0	0	0
July 2013	B4A01	0	0	0	0	0	0	0	0
July 2013	B4A02	0	0	0	0	0	0	0	0
July 2013	B4A04	0	0	0	0	0	0	0	0
July 2013	B4A06	0	0	0	0	0	0	0	0
July 2013	B4A07	0	0	0	0	0	0	0	0
July 2013	B4A08	0	18	0	0	0	0	0	0
July 2013	B4A09	0	0	0	0	0	0	0	0
July 2013	B4A10	0	0	0	0	0	0	0	0
July 2013	B4A11	0	0	0	0	0	0	0	0
July 2013	B4A12	0	0	0	0	0	0	0	0
July 2013	B4A13	0	0	0	0	0	0	0	0
July 2013	B4A14	0	2	0	0	0	0	0	0
July 2013	B4A15	0	0	0	0	0	0	0	0
July 2013	B4A16	0	0	0	0	0	0	0	0

Date	Plot	*Ceratozetes psammophilus*	*Scheloribates initialis*	*Microppia minus*	*Zygoribatula frisiae*	*Protoribates capucinus*	*Tectocepheus minor*	*Hypochthonius rufulus*	*Steganacarus striculus*
July 2013	B4A17	0	0	0	0	0	0	0	0
July 2013	B4A18	0	0	0	0	0	0	0	0
July 2013	B4A20	0	0	0	0	0	0	0	0
July 2013	B4A21	0	0	0	0	0	0	0	0
July 2013	B4A22	0	0	0	0	0	0	0	0
July 2013	B2A01	0	0	0	4	0	0	0	0
July 2013	B2A02	0	0	0	0	0	0	0	0
July 2013	B2A03	0	0	0	0	0	0	0	0
July 2013	B2A04	0	0	0	0	0	0	0	0
July 2013	B2A05	0	0	0	0	0	0	0	0
July 2013	B2A06	0	0	0	0	0	0	0	0
July 2013	B2A08	0	0	0	1	0	0	0	0
July 2013	B2A09	0	0	0	0	0	0	0	0
July 2013	B2A10	0	0	0	0	0	0	0	0
July 2013	B2A12	0	0	0	0	0	0	0	0
July 2013	B2A13	0	0	0	0	0	0	0	0
July 2013	B2A14	0	0	0	0	0	0	0	0
July 2013	B2A15	0	0	0	0	0	0	0	0
July 2013	B2A16	0	0	0	0	0	0	0	0
July 2013	B2A17	0	0	0	0	0	0	0	0
July 2013	B2A18	0	0	0	0	0	0	0	0
July 2013	B2A19	0	0	0	0	0	0	0	0
July 2013	B2A20	0	0	0	0	0	0	0	0
July 2013	B2A21	0	0	0	0	0	0	0	0
July 2013	B2A22	0	0	0	0	0	0	0	0
July 2013	B3A01	0	0	0	0	0	0	0	0
July 2013	B3A02	0	0	0	1	0	0	0	0
July 2013	B3A03	0	0	0	0	0	0	0	0
July 2013	B3A04	0	0	0	0	0	0	0	0
July 2013	B3A05	0	0	0	0	0	0	0	0
July 2013	B3A06	0	0	0	0	0	0	0	0
July 2013	B3A07	0	0	0	0	0	0	0	0
July 2013	B3A08	0	0	0	0	0	0	0	0
July 2013	B3A09	0	0	0	0	0	0	0	0
July 2013	B3A11	0	0	0	0	0	0	0	0
July 2013	B3A12	0	0	0	0	0	0	0	0
July 2013	B3A13	0	0	0	1	0	0	0	0
July 2013	B3A14	0	0	0	0	0	0	0	0

Date	Plot	*Ceratozetes psammophilus*	*Scheloribates initialis*	*Microppia minus*	*Zygoribatula frisiae*	*Protoribates capucinus*	*Tectocepheus minor*	*Hypochthonius rufulus*	*Steganacarus striculus*
July 2013	B3A16	0	0	0	0	0	0	0	0
July 2013	B3A17	0	0	0	0	0	0	0	0
July 2013	B3A19	0	0	0	0	0	0	0	0
July 2013	B3A20	0	0	0	0	0	0	0	0
July 2013	B3A21	0	0	0	0	0	0	0	0
July 2013	B3A22	0	0	0	0	0	0	0	0
July 2013	B3A23	0	0	0	0	0	0	0	0
July 2013	B3A24	0	0	1	0	0	0	0	0
October 2013	B1A01	0	0	0	0	0	0	0	0
October 2013	B1A02	0	0	0	0	0	0	0	0
October 2013	B1A03	0	0	0	0	0	0	0	0
October 2013	B1A04	0	0	0	0	0	0	0	0
October 2013	B1A05	0	0	0	0	0	0	0	0
October 2013	B1A06	0	0	0	0	1	0	0	0
October 2013	B1A07	0	0	0	0	0	0	0	0
October 2013	B1A08	0	0	0	0	0	0	0	0
October 2013	B1A11	0	0	0	0	0	0	0	0
October 2013	B1A12	0	0	0	0	0	0	0	0
October 2013	B1A13	0	0	0	0	0	0	0	0
October 2013	B1A14	1	0	0	0	0	0	0	0
October 2013	B1A15	0	0	0	0	0	0	0	0
October 2013	B1A16	0	0	0	0	0	0	0	0
October 2013	B1A17	0	0	0	0	0	0	0	0
October 2013	B1A18	0	0	0	0	0	0	0	0
October 2013	B1A19	0	0	0	0	0	0	0	0
October 2013	B1A20	0	0	0	0	0	0	0	0
October 2013	B1A21	1	0	0	0	0	0	0	0
October 2013	B1A22	0	0	0	0	0	0	0	0
October 2013	B4A01	0	0	0	0	0	0	0	0
October 2013	B4A02	0	0	0	0	0	0	0	0
October 2013	B4A04	0	0	0	0	0	0	0	0
October 2013	B4A06	0	0	0	0	0	0	0	0
October 2013	B4A07	0	0	0	0	0	0	0	0
October 2013	B4A08	0	0	0	0	0	0	0	0
October 2013	B4A09	0	0	0	0	0	0	0	0
October 2013	B4A10	0	0	0	0	0	0	0	0
October 2013	B4A11	0	0	0	0	0	0	0	0
October 2013	B4A12	0	0	0	0	0	0	0	0

Date	Plot	*Ceratozetes psammophilus*	*Scheloribates initialis*	*Microppia minus*	*Zygoribatula frisiae*	*Protoribates capucinus*	*Tectocepheus minor*	*Hypochthonius rufulus*	*Steganacarus striculus*
October 2013	B4A13	0	0	0	0	0	0	0	0
October 2013	B4A14	0	0	0	0	0	0	0	0
October 2013	B4A15	0	0	0	0	0	0	0	0
October 2013	B4A16	0	0	0	0	0	0	0	0
October 2013	B4A17	0	0	1	0	0	0	0	0
October 2013	B4A18	0	0	0	0	0	0	0	0
October 2013	B4A20	0	0	0	0	0	0	0	0
October 2013	B4A21	0	0	0	0	0	0	0	0
October 2013	B4A22	0	0	0	0	0	0	0	0
October 2013	B2A01	0	0	0	0	0	0	0	0
October 2013	B2A02	0	0	0	30	0	2	0	0
October 2013	B2A03	0	0	0	0	0	1	0	0
October 2013	B2A04	0	0	0	1	0	0	0	0
October 2013	B2A05	0	0	0	0	0	0	0	0
October 2013	B2A06	0	0	0	0	0	0	1	0
October 2013	B2A08	6	0	0	0	0	0	0	0
October 2013	B2A09	0	0	0	0	0	3	0	0
October 2013	B2A10	0	0	0	0	0	0	0	0
October 2013	B2A12	0	0	0	0	0	0	0	0
October 2013	B2A13	0	0	0	0	0	0	0	0
October 2013	B2A14	0	0	0	0	0	0	0	0
October 2013	B2A15	0	0	0	0	0	0	0	0
October 2013	B2A16	0	0	0	0	0	0	0	0
October 2013	B2A17	0	0	0	0	0	0	0	0
October 2013	B2A18	0	0	0	0	0	0	0	0
October 2013	B2A19	0	0	0	1	0	0	0	0
October 2013	B2A20	0	0	0	0	0	0	0	0
October 2013	B2A21	0	0	0	0	0	0	0	1
October 2013	B2A22	0	0	0	0	0	0	0	0
October 2013	B3A01	0	0	0	0	0	0	0	0
October 2013	B3A02	1	0	0	0	0	0	0	0
October 2013	B3A03	0	0	0	0	0	0	0	0
October 2013	B3A04	0	0	0	0	0	0	0	0
October 2013	B3A05	0	0	0	0	0	0	0	0
October 2013	B3A06	0	0	0	0	0	0	0	0
October 2013	B3A07	0	0	0	0	0	0	0	0
October 2013	B3A08	0	0	0	0	0	0	0	0
October 2013	B3A09	1	0	0	0	0	0	0	0

Date	Plot	*Ceratozetes psammophilus*	*Scheloribates initialis*	*Microppia minus*	*Zygoribatula frisiae*	*Protoribates capucinus*	*Tectocepheus minor*	*Hypochthonius rufulus*	*Steganacarus stricul us*
October 2013	B3A11	0	0	0	0	0	0	0	0
October 2013	B3A12	0	0	0	0	0	0	0	0
October 2013	B3A13	0	0	0	0	0	0	0	0
October 2013	B3A14	0	0	0	0	0	0	0	0
October 2013	B3A16	0	0	0	0	0	0	0	0
October 2013	B3A17	0	0	0	0	0	0	0	0
October 2013	B3A19	0	0	0	0	0	1	0	0
October 2013	B3A20	0	0	0	0	0	0	0	0
October 2013	B3A21	0	0	0	0	0	0	0	0
October 2013	B3A22	0	0	0	0	0	0	0	0
October 2013	B3A23	0	0	0	0	0	0	0	0
October 2013	B3A24	0	0	0	0	0	0	0	0
