# Supplementary material for: Response of Collembola and Acari communities to summer flooding in a grassland plant diversity experiment
Source: PLoS One. 2018 Aug 30;13(8):e0202862. doi: 10.1371/journal.pone.0202862 (PMC6117009; doi:10.1371/journal.pone.0202862)
Supplement: S5 Table — (PDF) [file pone.0202862.s006.pdf]

**Dataset**

Abundance Families of Collembola
Type raw
Unit individuals in soil cores of 5 cm diameter and 5 cm depth

| Date | Plot | Total Collembola | Entomobryidae | Isotomidae | Tullbergiidae |
|---|---|---|---|---|---|
| November 2010 | B1A01 | 11 | 10 | 1 | 0 |
| November 2010 | B1A02 | 37 | 1 | 20 | 11 |
| November 2010 | B1A03 | 33 | 14 | 7 | 5 |
| November 2010 | B1A04 | 59 | 10 | 34 | 11 |
| November 2010 | B1A05 | 22 | 5 | 6 | 3 |
| November 2010 | B1A06 | 54 | 5 | 22 | 22 |
| November 2010 | B1A07 | 27 | 1 | 0 | 19 |
| November 2010 | B1A08 | 23 | 6 | 8 | 4 |
| November 2010 | B1A11 | 39 | 26 | 10 | 1 |
| November 2010 | B1A12 | 11 | 10 | 1 | 0 |
| November 2010 | B1A13 | 31 | 3 | 19 | 3 |
| November 2010 | B1A14 | 4 | 0 | 2 | 0 |
| November 2010 | B1A15 | 0 | 0 | 0 | 0 |
| November 2010 | B1A16 | 24 | 9 | 1 | 2 |
| November 2010 | B1A17 | 65 | 10 | 4 | 33 |
| November 2010 | B1A18 | 0 | 0 | 0 | 0 |
| November 2010 | B1A19 | 40 | 11 | 9 | 16 |
| November 2010 | B1A20 | 83 | 18 | 12 | 26 |
| November 2010 | B1A21 | 48 | 14 | 6 | 14 |
| November 2010 | B1A22 | 27 | 16 | 1 | 6 |
| November 2010 | B2A01 | 23 | 7 | 1 | 10 |
| November 2010 | B2A02 | 0 | 0 | 0 | 0 |
| November 2010 | B2A03 | 18 | 6 | 5 | 3 |
| November 2010 | B2A04 | 13 | 5 | 0 | 8 |
| November 2010 | B2A05 | 36 | 8 | 1 | 15 |
| November 2010 | B2A06 | 60 | 12 | 23 | 9 |
| November 2010 | B2A08 | 87 | 4 | 12 | 29 |
| November 2010 | B2A09 | 57 | 4 | 6 | 32 |
| November 2010 | B2A10 | 182 | 4 | 92 | 68 |
| November 2010 | B2A12 | 24 | 6 | 1 | 17 |
| November 2010 | B2A13 | 11 | 7 | 0 | 4 |
| November 2010 | B2A14 | 16 | 0 | 2 | 5 |
| November 2010 | B2A15 | 25 | 11 | 0 | 14 |
| November 2010 | B2A16 | 51 | 8 | 1 | 34 |
| November 2010 | B2A17 | 19 | 15 | 1 | 3 |
| November 2010 | B2A18 | 92 | 22 | 11 | 7 |
| November 2010 | B2A19 | 4 | 0 | 1 | 2 |
| November 2010 | B2A20 | 89 | 11 | 9 | 5 |
| November 2010 | B2A21 | 111 | 7 | 12 | 80 |
| November 2010 | B2A22 | 4 | 0 | 4 | 0 |
| November 2010 | B3A01 | 17 | 9 | 1 | 5 |
| November 2010 | B3A02 | 39 | 4 | 3 | 24 |
| November 2010 | B3A03 | 2 | 0 | 0 | 1 |
| November 2010 | B3A04 | 84 | 26 | 26 | 14 |

| Date | Plot | Total Collembola | Entomobryidae | Isotomidae | Tullbergiidae |
|---|---|---|---|---|---|
| November 2010 | B3A05 | 40 | 16 | 5 | 5 |
| November 2010 | B3A06 | 46 | 23 | 4 | 14 |
| November 2010 | B3A07 | 3 | 0 | 1 | 1 |
| November 2010 | B3A08 | 49 | 4 | 9 | 9 |
| November 2010 | B3A09 | 49 | 8 | 18 | 1 |
| November 2010 | B3A11 | 36 | 3 | 15 | 0 |
| November 2010 | B3A12 | 28 | 9 | 8 | 10 |
| November 2010 | B3A13 | 37 | 14 | 11 | 10 |
| November 2010 | B3A14 | 32 | 11 | 16 | 2 |
| November 2010 | B3A16 | 31 | 5 | 9 | 12 |
| November 2010 | B3A17 | 13 | 3 | 5 | 1 |
| November 2010 | B3A19 | 89 | 8 | 25 | 38 |
| November 2010 | B3A20 | 40 | 28 | 1 | 9 |
| November 2010 | B3A21 | 2 | 0 | 2 | 0 |
| November 2010 | B3A22 | 43 | 23 | 5 | 3 |
| November 2010 | B3A23 | 57 | 25 | 24 | 3 |
| November 2010 | B3A24 | 52 | 43 | 6 | 2 |
| November 2010 | B4A01 | 58 | 13 | 14 | 13 |
| November 2010 | B4A02 | 6 | 2 | 0 | 4 |
| November 2010 | B4A04 | 52 | 28 | 14 | 5 |
| November 2010 | B4A06 | 9 | 2 | 1 | 6 |
| November 2010 | B4A07 | 22 | 9 | 3 | 4 |
| November 2010 | B4A08 | 31 | 3 | 11 | 13 |
| November 2010 | B4A09 | 57 | 13 | 3 | 18 |
| November 2010 | B4A10 | 20 | 9 | 4 | 4 |
| November 2010 | B4A11 | 40 | 17 | 16 | 2 |
| November 2010 | B4A12 | 24 | 13 | 6 | 5 |
| November 2010 | B4A13 | 9 | 2 | 1 | 6 |
| November 2010 | B4A14 | 36 | 14 | 2 | 20 |
| November 2010 | B4A15 | 40 | 10 | 2 | 10 |
| November 2010 | B4A16 | 8 | 3 | 0 | 5 |
| November 2010 | B4A17 | 27 | 10 | 1 | 7 |
| November 2010 | B4A18 | 30 | 19 | 2 | 7 |
| November 2010 | B4A20 | 9 | 2 | 1 | 6 |
| November 2010 | B4A21 | 13 | 1 | 0 | 10 |
| November 2010 | B4A22 | 18 | 15 | 0 | 3 |
| July 2013 | B1A01 | 1 | 0 | 0 | 0 |
| July 2013 | B1A02 | 0 | 0 | 0 | 0 |
| July 2013 | B1A03 | 0 | 0 | 0 | 0 |
| July 2013 | B1A04 | 0 | 0 | 0 | 0 |
| July 2013 | B1A05 | 0 | 0 | 0 | 0 |
| July 2013 | B1A06 | 0 | 0 | 0 | 0 |
| July 2013 | B1A07 | 0 | 0 | 0 | 0 |
| July 2013 | B1A08 | 0 | 0 | 0 | 0 |
| July 2013 | B1A11 | 1 | 0 | 1 | 0 |
| July 2013 | B1A12 | 1 | 0 | 1 | 0 |
| July 2013 | B1A13 | 0 | 0 | 0 | 0 |
| July 2013 | B1A14 | 2 | 0 | 0 | 0 |
| July 2013 | B1A15 | 0 | 0 | 0 | 0 |

| Date | Plot | Total Collembola | Entomobryidae | Isotomidae | Tullbergiidae |
|---|---|---|---|---|---|
| July 2013 | B1A16 | 0 | 0 | 0 | 0 |
| July 2013 | B1A17 | 0 | 0 | 0 | 0 |
| July 2013 | B1A18 | 0 | 0 | 0 | 0 |
| July 2013 | B1A19 | 0 | 0 | 0 | 0 |
| July 2013 | B1A20 | 1 | 0 | 0 | 1 |
| July 2013 | B1A21 | 0 | 0 | 0 | 0 |
| July 2013 | B1A22 | 1 | 0 | 0 | 1 |
| July 2013 | B2A01 | 1 | 0 | 1 | 0 |
| July 2013 | B2A02 | 0 | 0 | 0 | 0 |
| July 2013 | B2A03 | 0 | 0 | 0 | 0 |
| July 2013 | B2A04 | 0 | 0 | 0 | 0 |
| July 2013 | B2A05 | 0 | 0 | 0 | 0 |
| July 2013 | B2A06 | 2 | 1 | 1 | 0 |
| July 2013 | B2A08 | 0 | 0 | 0 | 0 |
| July 2013 | B2A09 | 0 | 0 | 0 | 0 |
| July 2013 | B2A10 | 0 | 0 | 0 | 0 |
| July 2013 | B2A12 | 10 | 8 | 2 | 0 |
| July 2013 | B2A13 | 1 | 0 | 0 | 0 |
| July 2013 | B2A14 | 2 | 0 | 1 | 1 |
| July 2013 | B2A15 | 0 | 0 | 0 | 0 |
| July 2013 | B2A16 | 13 | 7 | 4 | 0 |
| July 2013 | B2A17 | 1 | 0 | 0 | 0 |
| July 2013 | B2A18 | 0 | 0 | 0 | 0 |
| July 2013 | B2A19 | 0 | 0 | 0 | 0 |
| July 2013 | B2A20 | 0 | 0 | 0 | 0 |
| July 2013 | B2A21 | 1 | 1 | 0 | 0 |
| July 2013 | B2A22 | 0 | 0 | 0 | 0 |
| July 2013 | B3A01 | 0 | 0 | 0 | 0 |
| July 2013 | B3A02 | 0 | 0 | 0 | 0 |
| July 2013 | B3A03 | 0 | 0 | 0 | 0 |
| July 2013 | B3A04 | 0 | 0 | 0 | 0 |
| July 2013 | B3A05 | 0 | 0 | 0 | 0 |
| July 2013 | B3A06 | 0 | 0 | 0 | 0 |
| July 2013 | B3A07 | 0 | 0 | 0 | 0 |
| July 2013 | B3A08 | 0 | 0 | 0 | 0 |
| July 2013 | B3A09 | 2 | 2 | 0 | 0 |
| July 2013 | B3A11 | 0 | 0 | 0 | 0 |
| July 2013 | B3A12 | 0 | 0 | 0 | 0 |
| July 2013 | B3A13 | 2 | 2 | 0 | 0 |
| July 2013 | B3A14 | 0 | 0 | 0 | 0 |
| July 2013 | B3A16 | 1 | 0 | 1 | 0 |
| July 2013 | B3A17 | 0 | 0 | 0 | 0 |
| July 2013 | B3A19 | 0 | 0 | 0 | 0 |
| July 2013 | B3A20 | 0 | 0 | 0 | 0 |
| July 2013 | B3A21 | 0 | 0 | 0 | 0 |
| July 2013 | B3A22 | 2 | 0 | 1 | 0 |
| July 2013 | B3A23 | 1 | 0 | 1 | 0 |
| July 2013 | B3A24 | 0 | 0 | 0 | 0 |
| July 2013 | B4A01 | 0 | 0 | 0 | 0 |

| Date | Plot | Total Collembola | Entomobryidae | Isotomidae | Tullbergiidae |
|---|---|---|---|---|---|
| July 2013 | B4A02 | 1 | 0 | 1 | 0 |
| July 2013 | B4A04 | 2 | 1 | 1 | 0 |
| July 2013 | B4A06 | 2 | 0 | 2 | 0 |
| July 2013 | B4A07 | 0 | 0 | 0 | 0 |
| July 2013 | B4A08 | 0 | 0 | 0 | 0 |
| July 2013 | B4A09 | 13 | 12 | 1 | 0 |
| July 2013 | B4A10 | 1 | 1 | 0 | 0 |
| July 2013 | B4A11 | 0 | 0 | 0 | 0 |
| July 2013 | B4A12 | 0 | 0 | 0 | 0 |
| July 2013 | B4A13 | 0 | 0 | 0 | 0 |
| July 2013 | B4A14 | 2 | 0 | 0 | 0 |
| July 2013 | B4A15 | 0 | 0 | 0 | 0 |
| July 2013 | B4A16 | 0 | 0 | 0 | 0 |
| July 2013 | B4A17 | 1 | 1 | 0 | 0 |
| July 2013 | B4A18 | 2 | 2 | 0 | 0 |
| July 2013 | B4A20 | 0 | 0 | 0 | 0 |
| July 2013 | B4A21 | 1 | 1 | 0 | 0 |
| July 2013 | B4A22 | 0 | 0 | 0 | 0 |
| October 2013 | B1A01 | 47 | 24 | 22 | 0 |
| October 2013 | B1A02 | 6 | 6 | 0 | 0 |
| October 2013 | B1A03 | 38 | 2 | 35 | 0 |
| October 2013 | B1A04 | 26 | 14 | 12 | 0 |
| October 2013 | B1A05 | 0 | 0 | 0 | 0 |
| October 2013 | B1A06 | 85 | 10 | 49 | 8 |
| October 2013 | B1A07 | 90 | 55 | 31 | 0 |
| October 2013 | B1A08 | 10 | 7 | 2 | 1 |
| October 2013 | B1A11 | 12 | 2 | 1 | 9 |
| October 2013 | B1A12 | 9 | 3 | 5 | 1 |
| October 2013 | B1A13 | 0 | 0 | 0 | 0 |
| October 2013 | B1A14 | 15 | 10 | 1 | 1 |
| October 2013 | B1A15 | 9 | 4 | 2 | 1 |
| October 2013 | B1A16 | 15 | 5 | 0 | 9 |
| October 2013 | B1A17 | 23 | 7 | 16 | 0 |
| October 2013 | B1A18 | 21 | 19 | 1 | 1 |
| October 2013 | B1A19 | 15 | 8 | 0 | 5 |
| October 2013 | B1A20 | 65 | 4 | 1 | 57 |
| October 2013 | B1A21 | 24 | 9 | 15 | 0 |
| October 2013 | B1A22 | 65 | 6 | 11 | 48 |
| October 2013 | B2A01 | 26 | 9 | 1 | 13 |
| October 2013 | B2A02 | 100 | 57 | 21 | 7 |
| October 2013 | B2A03 | 31 | 7 | 4 | 20 |
| October 2013 | B2A04 | 5 | 4 | 0 | 0 |
| October 2013 | B2A05 | 14 | 1 | 12 | 0 |
| October 2013 | B2A06 | 4 | 4 | 0 | 0 |
| October 2013 | B2A08 | 12 | 6 | 6 | 0 |
| October 2013 | B2A09 | 62 | 2 | 0 | 59 |
| October 2013 | B2A10 | 39 | 8 | 15 | 16 |
| October 2013 | B2A12 | 27 | 16 | 0 | 11 |
| October 2013 | B2A13 | 6 | 4 | 2 | 0 |

| Date | Plot | Total Collembola | Entomobryidae | Isotomidae | Tullbergiidae |
|---|---|---|---|---|---|
| October 2013 | B2A14 | 34 | 5 | 15 | 13 |
| October 2013 | B2A15 | 26 | 23 | 3 | 0 |
| October 2013 | B2A16 | 87 | 43 | 34 | 10 |
| October 2013 | B2A17 | 78 | 13 | 10 | 55 |
| October 2013 | B2A18 | 90 | 1 | 34 | 55 |
| October 2013 | B2A19 | 134 | 39 | 33 | 62 |
| October 2013 | B2A20 | 4 | 2 | 1 | 1 |
| October 2013 | B2A21 | 32 | 12 | 11 | 8 |
| October 2013 | B2A22 | 124 | 90 | 20 | 14 |
| October 2013 | B3A01 | 90 | 66 | 14 | 10 |
| October 2013 | B3A02 | 34 | 30 | 4 | 0 |
| October 2013 | B3A03 | 3 | 3 | 0 | 0 |
| October 2013 | B3A04 | 8 | 2 | 0 | 6 |
| October 2013 | B3A05 | 48 | 30 | 15 | 3 |
| October 2013 | B3A06 | 38 | 24 | 6 | 8 |
| October 2013 | B3A07 | 131 | 109 | 14 | 8 |
| October 2013 | B3A08 | 13 | 3 | 5 | 5 |
| October 2013 | B3A09 | 7 | 4 | 2 | 1 |
| October 2013 | B3A11 | 28 | 9 | 11 | 8 |
| October 2013 | B3A12 | 61 | 42 | 16 | 3 |
| October 2013 | B3A13 | 8 | 7 | 1 | 0 |
| October 2013 | B3A14 | 116 | 32 | 12 | 71 |
| October 2013 | B3A16 | 119 | 9 | 0 | 110 |
| October 2013 | B3A17 | 39 | 25 | 14 | 0 |
| October 2013 | B3A19 | 78 | 71 | 7 | 0 |
| October 2013 | B3A20 | 13 | 3 | 2 | 8 |
| October 2013 | B3A21 | 12 | 4 | 6 | 2 |
| October 2013 | B3A22 | 63 | 58 | 5 | 0 |
| October 2013 | B3A23 | 15 | 8 | 6 | 0 |
| October 2013 | B3A24 | 45 | 10 | 15 | 20 |
| October 2013 | B4A01 | 55 | 16 | 17 | 22 |
| October 2013 | B4A02 | 59 | 53 | 2 | 3 |
| October 2013 | B4A04 | 18 | 3 | 4 | 11 |
| October 2013 | B4A06 | 26 | 15 | 11 | 0 |
| October 2013 | B4A07 | 51 | 31 | 4 | 16 |
| October 2013 | B4A08 | 108 | 85 | 17 | 5 |
| October 2013 | B4A09 | 36 | 5 | 13 | 4 |
| October 2013 | B4A10 | 45 | 6 | 26 | 10 |
| October 2013 | B4A11 | 16 | 2 | 8 | 6 |
| October 2013 | B4A12 | 10 | 6 | 2 | 2 |
| October 2013 | B4A13 | 18 | 0 | 2 | 16 |
| October 2013 | B4A14 | 62 | 22 | 3 | 36 |
| October 2013 | B4A15 | 43 | 30 | 8 | 0 |
| October 2013 | B4A16 | 13 | 11 | 2 | 0 |
| October 2013 | B4A17 | 8 | 4 | 4 | 0 |
| October 2013 | B4A18 | 14 | 7 | 2 | 4 |
| October 2013 | B4A20 | 44 | 2 | 15 | 27 |
| October 2013 | B4A21 | 33 | 1 | 3 | 28 |
| October 2013 | B4A22 | 35 | 6 | 11 | 18 |
