# Supplementary material for: Response of Collembola and Acari communities to summer flooding in a grassland plant diversity experiment
Source: PLoS One. 2018 Aug 30;13(8):e0202862. doi: 10.1371/journal.pone.0202862 (PMC6117009; doi:10.1371/journal.pone.0202862)
Supplement: S6 Table — (PDF) [file pone.0202862.s007.pdf]

Dataset

Abundace Orders of Acari
Type raw
Unit individuals in soil cores of 5 cm diameter and 5 cm depth

Date	Plot	Total Acari	Oribatida	Gamasina	Prostigmata	Asigmata
November 2010	B1A01	74	24	12	37	1
November 2010	B1A02	46	4	26	14	2
November 2010	B1A03	103	53	31	9	10
November 2010	B1A04	284	252	31	1	0
November 2010	B1A05	19	5	13	1	0
November 2010	B1A06	72	24	25	18	5
November 2010	B1A07	42	2	36	4	0
November 2010	B1A08	44	0	35	6	1
November 2010	B1A11	171	146	6	15	4
November 2010	B1A12	15	2	4	0	9
November 2010	B1A14	27	19	6	2	0
November 2010	B1A16	74	1	59	13	1
November 2010	B1A17	16	5	3	8	0
November 2010	B1A19	36	18	13	4	1
November 2010	B1A20	30	15	13	2	0
November 2010	B1A21	59	32	17	10	0
November 2010	B1A22	41	24	3	14	0
November 2010	B2A01	25	3	9	13	0
November 2010	B2A03	47	10	11	11	15
November 2010	B2A04	14	1	4	8	1
November 2010	B2A05	21	12	7	2	0
November 2010	B2A06	52	21	17	14	0
November 2010	B2A08	95	13	33	20	29
November 2010	B2A09	43	14	23	6	0
November 2010	B2A10	65	14	15	4	32
November 2010	B2A12	14	3	9	1	1
November 2010	B2A13	4	2	1	1	0
November 2010	B2A14	36	19	5	3	9
November 2010	B2A15	20	3	13	3	1
November 2010	B2A16	19	1	12	5	1
November 2010	B2A17	21	2	10	1	8
November 2010	B2A18	63	23	31	7	1
November 2010	B2A19	10	7	2	1	0
November 2010	B2A20	65	10	39	10	6
November 2010	B2A21	31	3	11	12	5
November 2010	B2A22	5	1	3	0	1
November 2010	B3A01	6	3	1	1	1
November 2010	B3A02	16	2	10	4	0
November 2010	B3A03	19	1	15	0	3
November 2010	B3A04	176	11	21	18	126
November 2010	B3A05	37	8	9	3	17
November 2010	B3A06	91	41	19	25	6
November 2010	B3A07	4	1	1	0	2
November 2010	B3A08	32	10	16	3	3
November 2010	B3A09	19	11	6	1	1

Date	Plot	Total Acari	Oribatida	Gamasina	Prostigmata	Asigmata
November 2010	B3A11	56	27	13	9	7
November 2010	B3A12	8	0	7	0	1
November 2010	B3A13	47	15	16	12	4
November 2010	B3A14	24	2	15	7	0
November 2010	B3A16	17	2	11	1	3
November 2010	B3A17	19	0	14	5	0
November 2010	B3A19	41	24	0	12	5
November 2010	B3A20	33	24	7	2	0
November 2010	B3A21	1	0	1	0	0
November 2010	B3A22	120	18	5	89	8
November 2010	B3A23	44	6	6	3	29
November 2010	B3A24	47	12	22	6	7
November 2010	B4A01	108	47	15	35	11
November 2010	B4A02	0	0	0	0	0
November 2010	B4A04	105	64	14	25	2
November 2010	B4A06	18	0	11	0	7
November 2010	B4A07	48	10	14	3	21
November 2010	B4A08	35	10	5	11	9
November 2010	B4A09	12	1	10	1	0
November 2010	B4A10	73	5	18	15	35
November 2010	B4A11	17	0	11	2	4
November 2010	B4A12	9	0	6	0	3
November 2010	B4A13	11	2	7	0	2
November 2010	B4A14	5	0	5	0	0
November 2010	B4A15	7	0	6	0	1
November 2010	B4A17	10	0	5	3	2
November 2010	B4A18	14	1	0	7	6
November 2010	B4A20	2	0	2	0	0
November 2010	B4A21	19	2	13	2	2
November 2010	B4A22	15	0	7	0	8
July 2013	B1A01	10	6	1	0	3
July 2013	B1A02	0	0	0	0	0
July 2013	B1A03	0	0	0	0	0
July 2013	B1A04	0	0	0	0	0
July 2013	B1A05	0	0	0	0	0
July 2013	B1A06	0	0	0	0	0
July 2013	B1A07	0	0	0	0	0
July 2013	B1A08	0	0	0	0	0
July 2013	B1A11	8	3	0	5	0
July 2013	B1A12	2	0	2	0	0
July 2013	B1A13	2	2	0	0	0
July 2013	B1A14	0	0	0	0	0
July 2013	B1A15	6	0	0	5	0
July 2013	B1A16	8	2	0	6	0
July 2013	B1A17	1	0	0	1	0
July 2013	B1A18	3	0	0	3	0
July 2013	B1A19	7	3	0	4	0
July 2013	B1A20	17	4	0	14	0
July 2013	B1A21	16	0	1	14	0
July 2013	B1A22	1	0	1	0	0
July 2013	B4A01	1	0	1	0	0

Date	Plot	Total Acari	Oribatida	Gamasina	Prostigmata	Asigmata
July 2013	B4A02	3	1	2	0	0
July 2013	B4A04	1	1	0	0	0
July 2013	B4A06	6	1	0	5	0
July 2013	B4A07	2	1	0	1	0
July 2013	B4A08	23	23	0	0	0
July 2013	B4A09	26	9	0	17	0
July 2013	B4A10	3	1	2	0	0
July 2013	B4A11	0	0	0	0	0
July 2013	B4A12	1	1	0	0	0
July 2013	B4A13	1	0	1	0	0
July 2013	B4A14	2	2	0	0	0
July 2013	B4A15	3	1	1	1	0
July 2013	B4A16	0	0	0	0	0
July 2013	B4A17	1	0	0	1	0
July 2013	B4A18	4	3	1	0	0
July 2013	B4A20	0	0	0	0	0
July 2013	B4A21	0	0	0	0	0
July 2013	B4A22	3	0	2	0	1
July 2013	B2A01	9	6	3	0	0
July 2013	B2A02	2	2	0	0	0
July 2013	B2A03	0	0	0	0	0
July 2013	B2A04	0	0	0	0	0
July 2013	B2A05	1	0	0	1	0
July 2013	B2A06	2	0	2	0	0
July 2013	B2A08	10	5	5	0	0
July 2013	B2A09	29	12	2	10	3
July 2013	B2A10	2	0	1	1	0
July 2013	B2A12	5	2	2	1	0
July 2013	B2A13	3	0	0	3	0
July 2013	B2A14	0	0	0	0	0
July 2013	B2A15	0	0	0	0	0
July 2013	B2A16	8	0	1	7	0
July 2013	B2A17	1	0	1	0	0
July 2013	B2A18	2	0	0	2	0
July 2013	B2A19	6	0	0	5	1
July 2013	B2A20	27	0	2	25	0
July 2013	B2A21	9	1	1	7	0
July 2013	B2A22	6	3	0	3	0
July 2013	B3A01	0	0	0	0	0
July 2013	B3A02	5	4	0	1	0
July 2013	B3A03	1	0	0	1	0
July 2013	B3A04	0	0	0	0	0
July 2013	B3A05	0	0	0	0	0
July 2013	B3A06	0	0	0	0	0
July 2013	B3A07	6	0	0	4	2
July 2013	B3A08	2	0	0	2	0
July 2013	B3A09	4	0	0	1	3
July 2013	B3A11	6	5	0	1	0
July 2013	B3A12	6	2	0	3	1
July 2013	B3A13	1	1	0	0	0
July 2013	B3A14	0	0	0	0	0

Date	Plot	Total Acari	Oribatida	Gamasina	Prostigmata	Asigmata
July 2013	B3A16	1	0	0	1	0
July 2013	B3A17	0	0	0	0	0
July 2013	B3A19	0	0	0	0	0
July 2013	B3A20	0	0	0	0	0
July 2013	B3A21	3	3	0	0	0
July 2013	B3A22	0	0	0	0	0
July 2013	B3A23	0	0	0	0	0
July 2013	B3A24	11	10	0	1	0
October 2013	B1A01	43	21	11	7	2
October 2013	B1A02	20	18	1	1	0
October 2013	B1A03	58	20	22	9	7
October 2013	B1A04	27	7	14	5	0
October 2013	B1A05	0	0	0	0	0
October 2013	B1A06	128	63	20	42	3
October 2013	B1A07	75	20	10	8	37
October 2013	B1A08	0	0	0	0	0
October 2013	B1A11	57	43	3	11	0
October 2013	B1A12	12	1	2	9	0
October 2013	B1A13	2	0	0	1	1
October 2013	B1A14	199	157	30	12	0
October 2013	B1A15	8	2	0	6	0
October 2013	B1A16	101	18	43	40	0
October 2013	B1A17	19	3	1	14	1
October 2013	B1A18	104	0	2	97	5
October 2013	B1A19	23	2	2	19	0
October 2013	B1A20	52	18	11	23	0
October 2013	B1A21	57	11	16	30	0
October 2013	B1A22	30	14	8	7	1
October 2013	B4A01	57	20	2	26	9
October 2013	B4A02	44	13	12	19	0
October 2013	B4A04	51	24	4	23	0
October 2013	B4A06	6	3	3	0	0
October 2013	B4A07	64	14	27	20	3
October 2013	B4A08	194	51	99	35	9
October 2013	B4A09	66	22	6	38	0
October 2013	B4A10	35	10	5	15	5
October 2013	B4A11	7	4	2	1	0
October 2013	B4A12	30	6	1	23	0
October 2013	B4A13	2	0	1	1	0
October 2013	B4A14	14	4	5	5	0
October 2013	B4A15	334	20	17	7	290
October 2013	B4A16	186	3	10	171	2
October 2013	B4A17	20	3	9	6	2
October 2013	B4A18	99	49	9	32	9
October 2013	B4A20	11	6	3	2	0
October 2013	B4A21	34	18	3	13	0
October 2013	B4A22	120	18	6	12	84
October 2013	B2A01	23	18	5	0	0
October 2013	B2A02	115	103	8	4	0
October 2013	B2A03	28	23	5	0	0
October 2013	B2A04	10	2	4	4	0

Date	Plot	Total Acari	Oribatida	Gamasina	Prostigmata	Asigmata
October 2013	B2A05	9	2	0	7	0
October 2013	B2A06	8	8	0	0	0
October 2013	B2A08	38	19	3	16	0
October 2013	B2A09	57	19	3	35	0
October 2013	B2A10	29	0	29	0	0
October 2013	B2A12	13	8	4	1	0
October 2013	B2A13	37	0	1	36	0
October 2013	B2A14	41	15	12	14	0
October 2013	B2A15	21	0	1	20	0
October 2013	B2A16	55	3	52	0	0
October 2013	B2A17	34	2	32	0	0
October 2013	B2A18	23	13	4	6	0
October 2013	B2A19	36	11	12	12	1
October 2013	B2A20	1	0	0	1	0
October 2013	B2A21	143	102	33	8	0
October 2013	B2A22	69	48	8	1	12
October 2013	B3A01	41	21	11	9	0
October 2013	B3A02	31	1	28	0	2
October 2013	B3A03	1	0	1	0	0
October 2013	B3A04	33	5	0	9	19
October 2013	B3A05	32	15	5	12	0
October 2013	B3A06	140	33	2	84	21
October 2013	B3A07	63	29	20	14	0
October 2013	B3A08	137	5	3	129	0
October 2013	B3A09	8	2	2	4	0
October 2013	B3A11	234	72	7	155	0
October 2013	B3A12	37	7	0	30	0
October 2013	B3A13	4	1	2	1	0
October 2013	B3A14	64	22	7	22	13
October 2013	B3A16	26	6	3	10	7
October 2013	B3A17	6	0	3	3	0
October 2013	B3A19	31	23	7	1	0
October 2013	B3A20	44	1	7	33	3
October 2013	B3A21	30	3	3	24	0
October 2013	B3A22	40	15	3	22	0
October 2013	B3A23	27	16	8	3	0
October 2013	B3A24	91	22	27	21	21
